# Cancer stem cells are hyper-responsive sensors of the tumour microenvironment and regulate metastasis dynamics through YAP/TAZ

**DOI:** 10.1038/s41556-026-02013-8

**Published:** 2026-07-27

**Authors:** Binwu Tang, Jacob Minin, Victoria M. Gonzalez, Zoya Z. Khan, Andrew R. Gaines, Yuval Raviv, Yu-an Yang, Christine P. Carney, Zachary G. Millman, Daniel Grun, Cristiana M. Pineda, Alina Sharma, Dominic Esposito, Hualong Yan, Jing Huang, Andy D. Tran, Michael Kruhlak, Howard H. Yang, Maxwell P. Lee, Lalage M. Wakefield

**Affiliations:** 1 Laboratory of Cancer Biology and Genetics, Center for Cancer Research, National Cancer Institute, National Institutes of Health, Bethesda, MD USA; 2 Protein Expression Laboratory, Frederick National Laboratory for Cancer Research, Frederick, MD USA

**Keywords:** Cancer stem cells, Cancer microenvironment, Metastasis, Breast cancer

## Abstract

Cancer stem cells (CSCs) drive metastasis and therapy resistance, yet their behaviour within the complex tumour microenvironment remains poorly understood. Here we use a fluorescent reporter that marks CSCs to show that CSCs and their more differentiated progeny display strikingly different population dynamics during metastatic lung colonization in breast cancer models. CSC expansion is rapidly curtailed early in colonization, suggesting a strong negative feedback mechanism acting selectively on this subpopulation. We showed that CSCs are exceptionally sensitive to local microenvironmental cues such as cell crowding and nutrient availability. They respond earlier and more extensively than their differentiated progeny, thereby coupling tumour growth to resource and space availability. Microenvironmental signals converge on the transcriptional regulatory complex YAP/TAZ/TEAD, with CSC sensitivity arising from elevated signal reception and greater chromatin accessibility at TEAD-regulated enhancers. Targeting upstream inputs to this pathway reversed chemotherapy-induced CSC enrichment in lung metastases, suggesting a potential therapeutic strategy.

## Abstract (web summary)

Using a dynamic fluorescent reporter, Tang et al. demonstrate that cancer stem cells are hyper-responsive to microenvironmental cues, which they sense and adapt to through YAP/TAZ signalling, thereby playing an essential role in breast cancer lung metastasis.

## Main

Many epithelial cancers are initiated and sustained by a small subpopulation of tumour cells with stem-like properties, the cancer stem cells (CSCs)^1,2^. CSCs are uniquely capable of indefinite self-renewal and give rise to the more differentiated progeny (nonCSCs) that form the bulk of the tumour. By contrast, individual nonCSCs can only undergo a limited number of cell divisions before exhausting their proliferative potential^3^. Metastasis is similarly driven by ‘metastasis-initiating cells’, which probably represent a subset of CSCs with acquired features that allow them to intravasate and survive the stresses of dissemination and distant colonization^4–6^. CSCs are often phenotypically similar to fetal somatic stem cells or facultative stem cells recruited for tissue repair^4,7,8^, suggesting that fetal or regenerative stem programmes are co-opted by oncogenically initiated cells to generate CSCs. The clinical importance of CSCs lies in their relative resistance to nearly all forms of therapy^1,9,10^, resulting from intrinsic properties such as elevated drug efflux pumps and DNA repair mechanisms, and from the existence of protective niches^9,11,12^. Consequently, CSCs drive therapeutic relapse and disease recurrence, and a better understanding of CSC dynamics will be critical for development of more effective therapeutics.

Like somatic stem cells, CSCs exist on a phenotypic continuum with the most ‘stem-like’ cells (CSCs) at one end and the most differentiated cells at the other^13^. However, phenotypic plasticity is higher in tumours than in normal tissues, enabling some bidirectional movement along this continuum^1,13^. The study of CSCs is inherently challenging due to this plasticity, their small numbers and the shortage of consensus markers for their identification in situ. To address these challenges, we developed a lentivirus-based fluorescent CSC reporter that reports on stemness as a dynamic phenotypic state, with good temporal resolution for live-cell imaging in vitro and in vivo^14,15^. Intravital imaging with this reporter in breast cancer models previously revealed dynamic attributes of CSCs in primary tumours, including the ability of macrophage contact to confer stem properties on nonCSCs^15^.

Here, we used this CSC reporter in breast cancer models to demonstrate very different population dynamics of CSCs and nonCSCs during early metastatic colonization of the lung, indicative of a strong form of negative regulation operating selectively on the CSCs. Modelling the observed dynamics in vitro, we uncovered a role for CSCs as hyper-responsive microenvironmental sensors and addressed underlying mechanisms and therapeutic implications.

## Results

### Breast CSCs are enriched in the circulation and on arrival in the lung

**Fig. 1:**
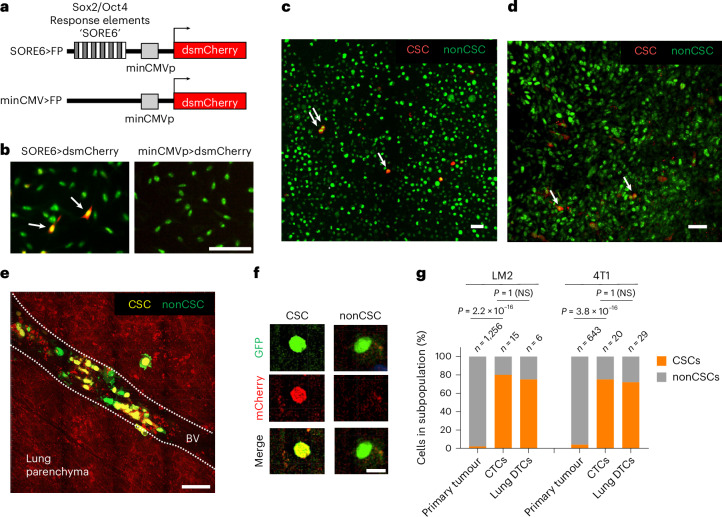
Breast CSCs are enriched in the circulation and on arrival in the lung. **a**, Schematic of SORE6 CSC reporter and matched control reporter. minCMVp is a minimal CMV promoter; FP, fluorescent protein. **b**, Epifluorescent images of LM2 cells transduced with the SORE6>dsmCherry reporter or minCMV>dsmCherry control construct growing in vitro. Representative of >1,000 images from >100 independent experiments. All tumour cells have a constitutive nuclear GFP marker. Arrows indicate CSCs. Scale bar, 100 µm. **c**,**d**, Confocal images of fresh orthotopic primary tumours from LM2 model (**c**) and 4T1 model (**d**), representative of *n* ≥ 3 tumours per model and multiple fields per tumour. Arrows indicate CSCs; double arrow indicates rare CSC doublet. Scale bars, 50 µm. **e**, High-resolution image of LM2 cells arriving in the lung 10 weeks after orthotopic implantation of a primary tumour. BV, blood vessel. Scale bar, 50 µm This event was seen only once. **f**, Image of circulating tumour cells from a mouse with an LM2 primary tumour, representative of 35 images. Scale bar, 10 µm. **g**, Quantitation of the percentage of CSCs in primary tumour, circulating tumour cells (CTCs) and single disseminated tumour cells (DTCs) in lungs, from LM2 or 4T1 orthotopic primary tumours. Two-sided Fisher’s exact test. Number of cells (*n*) assessed is indicated for each condition, pooled from *n* = 3 mice (LM2 primary tumour), *n* = 4 mice (4T1 primary tumour), *n* = 4 mice (LM2 CTCs and DTCs) or *n* = 9 mice (4T1 CTCs and DTCs). NS, not significant. Source data

**Extended Data Fig. 1:**
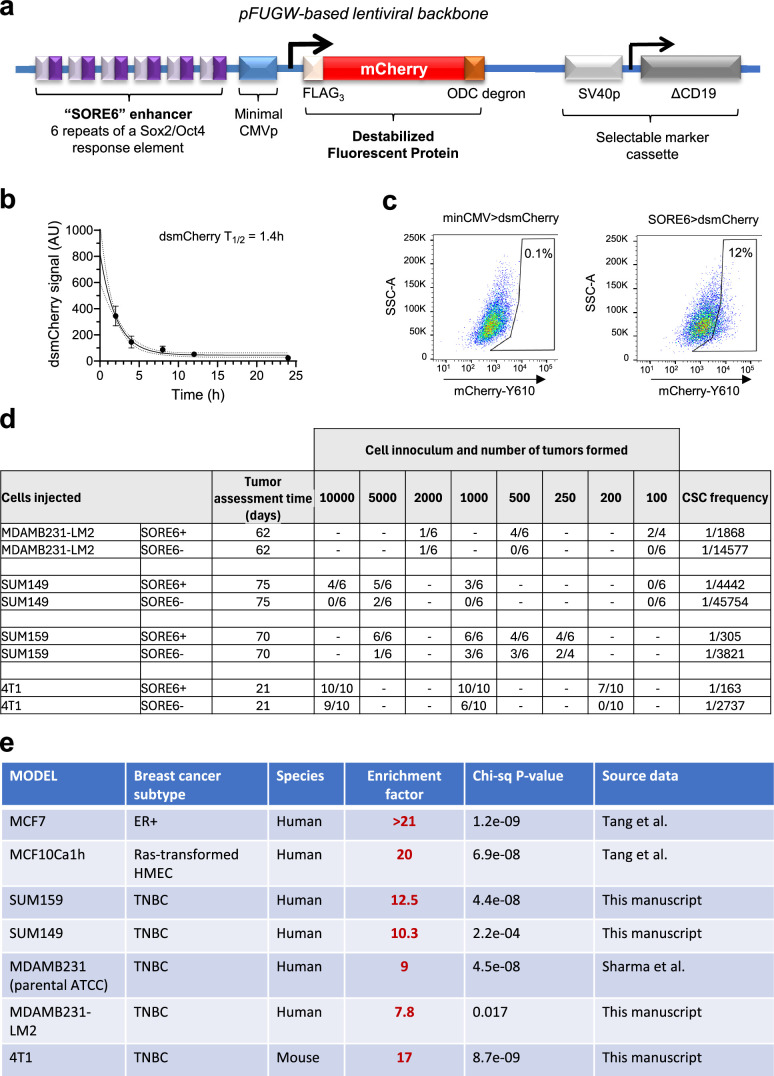
SORE6 reporter design and validation. **a**. Schematic of fluorescent reporter and selectable marker cassette used in this study. The SORE6 enhancer element coupled to a minimal cytomegalovirus (CMV) promoter region responds to the presence of stem cell master transcription factors SOX2 and OCT4 or their paralogs. The mCherry is destabilized with a C-terminal degron from the ornithine decarboxylase (ODC) gene and has an N-terminal 3xFLAG tag for improved detection by immunohistochemistry or immunofluorescence. The selectable marker cassette has a C-terminally truncated version of the human CD19 protein driven by the constitutive SV40 promoter for FACS-based selection of transduced cells. A matched control lentivirus lacks the SORE6 enhancer but is otherwise identical. **b**. The half-life of the dsmCherry in LM2 cells *in vitro* was determined by following the decay in dsmCherry signal after the addition of cycloheximide at t = 0 to block further protein synthesis. Mean +/- SD, n = 12. One phase decay curve fit. Dotted lines show 95% confidence intervals. Numerical source data are provided in the Source Data file. **c**. Flow cytometry profiles of transduced LM2 cells showing gating on minCMV>dsmCherry. Results are representative of >10 experiments. **d**. Results of *in vivo* limiting dilution assays for tumor-initiating cells in sorted SORE6+ and SORE6- cell populations from 3 different TNBC cell lines. **e**. Summary of ELDA results for 6 different breast cancer models showing 8-20x enrichment of tumor initiating ability in SORE6+ vs SORE6- cells across all models. Two-sided Chi-Square test for tumor-initiating frequency of CSCs vs nonCSCs. Source data

Our fluorescent lentiviral CSC reporter (SORE6>FP) dynamically reports on stemness through activation of an artificial enhancer (‘SORE6’) by the stemness master transcription factors OCT4 and SOX2 or their paralogues^14,15^. A control construct lacking the SORE6 enhancer but retaining the minimal CMV promoter (minCMV) is used for gating or thresholding (Fig. 1a and Extended Data Fig. 1a). The destabilized mCherry has a short half-life of ~1.4 h (Extended Data Fig. 1b), which confers high selectivity and temporal resolution to the reporter. For this study, tumour cells were transduced with a constitutive nuclear GFP marker (all tumour cell nuclei green), and either SORE6>dsmCherry (CSCs marked red/yellow) or the matched control construct. Representative images and flow cytometry profiles for transduced tumour cells are given in Fig. 1b and Extended Data Fig. 1c. Fluorescence-activated cell sorting (FACS)-sorted SORE6+ cells were significantly enriched for tumour-initiating ability by in vivo limiting dilution analysis in all models tested (Extended Data Fig. 1d,e), and are referred to as CSCs in our subsequent analyses. In breast cancer models, our reporter enriches both tumour-initiating and metastasis-initiating activity in the OCT4^hi^ SOX2^hi^ SORE6+ population^14^, and we use the umbrella term CSC throughout.

To address CSC population characteristics during the early stages of triple-negative breast cancer (TNBC) metastasis to the lung, we used the human MDAMB231-LM2 (hereafter ‘LM2’)^16^ and the mouse 4T1 models^17^. Due to the immunogenicity of the dsmCherry protein, the 4T1 model was used in immunocompromised mice. The SORE6 reporter marked a minor population of tumour cells in orthotopic primary tumours from LM2 (Fig. 1c) or 4T1 cells (Fig. 1d). CSCs typically appeared as isolated single cells, with only occasional doublets, usually towards the tumour edge. In Fig. 1e, we captured several tumour cells from an LM2 orthotopic primary tumour simultaneously arriving in the vasculature at the lung metastatic site; 25 of 32 tumours (78%) were CSCs, suggesting a strong enrichment of CSCs among intravasated cells. Indeed, circulating tumour cells recovered from the blood of mice with LM2 or 4T1 orthotopic primary tumours were 75–80% CSCs, compared with 1–2% CSCs in the originating primary tumours (Fig. 1f,g), confirming enrichment of CSCs in the circulation as previously observed by us and others^15,18^. Similarly, single disseminated tumour cells detected in the lungs of mice with orthotopic primary tumours were 72–75% CSCs, suggesting that this enrichment persists after extravasation (Fig. 1g). Thus, our imaging tool is performing as expected and enables quantitative insights into CSC and nonCSC populations during tumour progression.

### Different population dynamics for CSCs and nonCSCs in early lung colonization

**Fig. 2:**
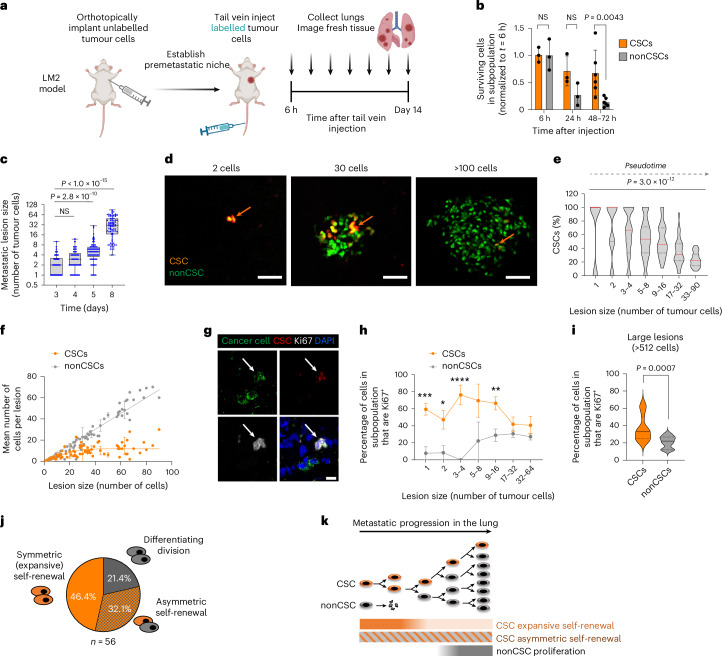
CSCs and nonCSCs have different population dynamics in early lung colonization. **a**, Schematic for lung metastasis study formats for LM2 model. **b**, Number of surviving LM2 tumour cells in each subpopulation (CSC versus nonCSC) in the lung, normalized to time *t* = 6 h. Results are mean ± s.d. for *n* = 3 (6 h and 24 h) or 6 mice (48–72 h) per timepoint. Two-sided Mann–Whitney *U* test. NS, not significant (*P* > 0.99 for 6 h and *P* = 0.10 for 24 h). **c**, Metastatic lesion size (number of tumour cells per lesion) as a function of time after tail-vein injection of LM2 cells. Boxplots show median ± interquartile range, with whiskers extending to maximum and minimum values, for pooled lesions from three mice per timepoint. Two-sided Dunns multiple comparisons test versus day 3. NS, not significant (*P* = 0.86). **d**, Images of LM2 lung metastatic lesions of different sizes, representative of hundreds of images from >50 mice. Arrow indicates representative CSC. Scale bar, 50 µm. **e**, CSCs as the percentage of total tumour cells in LM2 metastatic lesions of different sizes. Data pooled from multiple timepoints for a total of 26–88 lesions analysed per size bin from 12 mice. Red lines, medians; dotted lines, quartiles. Kruskal–Wallis test. **f**, Different kinetics for expansion of LM2 CSC and nonCSC populations in the lung as a function of lesion size following tail-vein injection. *n* = 1–63 depending on lesion size (pooled from 12 mice and 390 lesions over multiple timepoints). Data for lesion sizes with *n* ≥ 3 datapoints are plotted as mean ± s.d. **g**, LM2 lung metastases immunostained for Ki67 (proliferation), GFP (tumour cells) and mCherry (CSCs). Representative of >20 images from 6 mice. Arrow indicates CSC. Scale bar, 10 µm. **h**, Percentage of cells within CSC or nonCSC subpopulations that are Ki67^+^ in LM2 lung lesions of different sizes. Results are mean ± se.m., *n* = 3–54 lesions per size bin, from 6 mice. Two-sided unpaired *t*-test. *P = 0.0143, **P = 0.00187, ***P = 0.000612, ****P = 0.000073. **i**, Percentage of cells within subpopulations that are Ki67^+^, for larger lesions from an independent experiment (*n* = 15 metastases from 2 mice). Solid lines indicate medians, and dotted lines indicate quartiles. Two-sided Mann–Whitney *U* test). **j**, Composition of two-cell lesions in LM2 model (*n* = 56 lesions from 9 mice, days 3–5). Representative of two independent experiments. **k**, Model for CSC and nonCSC kinetics in early metastatic colonization. Schematic in **a** created in BioRender; Wakefield, L. https://biorender.com/1tbn57h (2026). Source data

**Extended Data Fig. 2:**
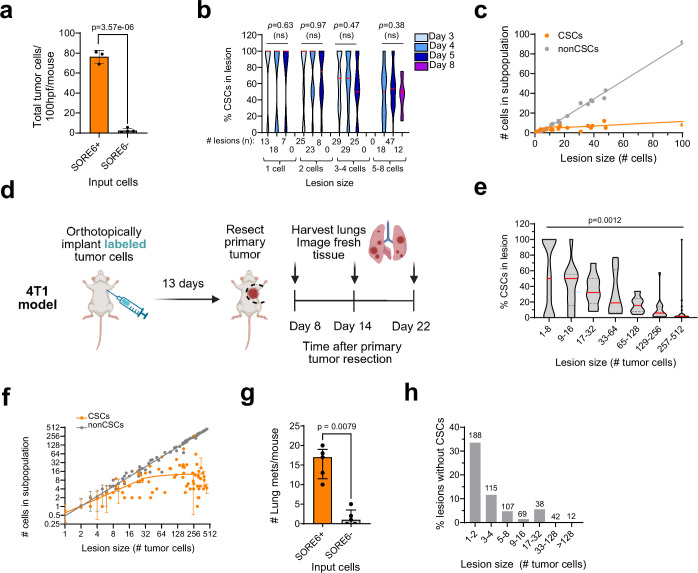
CSC and nonCSC population dynamics in early lung colonization. **a**. Total tumor cells in the lung 5 days after injection of FACS-sorted LM2 SORE6+ CSCs and SORE6- nonCSCs. Results are mean +/- SD for n = 3 (SORE6 + ) or 4 (SORE6-) mice/timepoint. Two-sided unpaired t test. **b**. % CSCs in metastatic lesions of specified sizes as a function of time after tail-vein injection of LM2 cells. Violin plots show medians (red lines) and quartiles (black dotted lines) for n = 7 to 47 lesions/size bin/timepoint as indicated on graph, pooled from 3 mice/timepoint. Two-sided Kruskal-Wallis test. **c**. # CSCs and nonCSCs per lesion as a function of metastatic lesion size in lungs of mice bearing orthotopic LM2 tumors at t = 8 weeks after tumor cell implantation. n = 4 mice **d**. Schematic for metastasis generation and imaging in the 4T1 model. **e**. % CSCs in lesion as a function of increasing metastatic lesion size in lungs from mice bearing 4T1 orthotopic primary tumors at t = 22, 28 and 35 days after tumor cell implantation. Red lines on violin plots indicate medians; dotted lines are quartiles for 7-43 lesions/size bin, pooled from 9 mice. Two-sided Kruskal-Wallis test. **f**. Expansion kinetics for CSCs and nonCSC populations in the lungs of mice with orthotopic 4T1 tumors. Results are mean +/- SD, where n = 3-29 depending on lesion size (pooled from n = 9 mice and n = 149 lesions over multiple timepoints). Where n = 1 or 2 for a given lesion size, individual results are plotted. Note log2 axes. **g**. Number of metastases/lung at day 21 in mice injected with 1000 FACS-sorted SORE6+ or SORE6- 4T1 cells via tailvein on day 0. Results are median +/- IQ range, n = 5 mice/group. Two-sided Mann-Whitney test. **h**. Metastatic lesions without CSCs as a function of lesion size in mice injected with unsorted LM2 cells via tail vein. Results are pooled from 5 mice. The number of lesions evaluated in each size bin is indicated on the graph. Numerical source data are provided in the Source Data file. Schematic in **d** created in BioRender; Wakefield, L. https://biorender.com/1tbn57h (2026). Source data

We next undertook a detailed analysis of CSC population dynamics following tumour cell arrival in the lung. To generate sufficient metastatic events, we used a pseudo-orthotopic format with tail vein injection for the LM2 model (Fig. 2a), and orthotopic implantation with primary tumour resection for the 4T1 model. Unless otherwise noted, unsorted tumour cell cultures containing both CSCs and nonCSCs were injected, and subpopulations in metastatic lesions were quantitated by ex vivo imaging of freshly excised lungs at different times. For the LM2 model, nonCSCs from unsorted cultures died off rapidly within 2 days of arrival in the lung, while CSCs mostly maintained their numbers (Fig. 2b). Injection of FACS-sorted LM2 CSCs and nonCSCs showed that only CSCs seeded metastases efficiently (Extended Data Fig. 2a). Thus CSCs have a selective early survival advantage in the lung and are uniquely capable of initiating progressively growing metastases.

A major advantage of our imaging approach is that the number of CSCs can be assessed for individual metastatic lesions. Median metastatic lesion size increased with time after tumour cell injection, but a wide range of metastasis sizes existed at each timepoint (Fig. 2c). The percentage of CSCs in a lesion of a given size was consistent over time (Extended Data Fig. 2b), allowing us to pool data across timepoints, with increasing lesion size generating a pseudotime trajectory. With this approach, we showed that the percentage of CSCs decreased as metastatic lesion size increased (Fig. 2d,e). Quantitating absolute CSC and nonCSC numbers in individual lesions revealed complex population dynamics. The CSC subpopulation reached an early plateau, while nonCSCs expanded continuously (Fig. 2f). Similar kinetics were observed for LM2 metastasis from the orthotopic primary site, though with fewer evaluable lesions, and for the 4T1 orthotopic model (Extended Data Fig. 2c–g).

To address the basis of these very different subpopulation dynamics, we assessed proliferation by co-immunostaining lungs for Ki67 (Fig. 2g). In the smallest lesions, most CSCs are proliferating, while the few nonCSCs are quiescent and only start to proliferate as lesions enlarge (Fig. 2h). Unexpectedly, CSCs were consistently more proliferative than nonCSCs, even in much larger metastases (Fig. 2i). Looking at the very earliest CSC division event by analysing the composition of two-cell lesions showed that nearly half were expansive self-renewing divisions (Fig. 2j), a third involved asymmetric divisions that jump-start nonCSC expansion, and the rest were fully differentiating divisions generating two nonCSCs. This latter is probably a metastatic dead-end, as we never observed lesions of >32 cells without a CSC (Extended Data Fig. 2h), suggesting that nonCSCs can undergo a maximum of 5 population doublings before exhausting their limited proliferative potential.

Overall, our data lead to a model for early metastasis dynamics (Fig. 2k). After arrival in the lung, most nonCSCs and some CSCs die off. Metastatic lesion initiation is driven solely by CSC proliferation, combining symmetric expansive divisions with asymmetric divisions that generate CSCs and nonCSCs. In larger lesions, the CSC population stops expanding despite ongoing CSC proliferation, so CSC divisions in larger metastases must be asymmetric (either physically or statistically). This strategy maintains CSC numbers while refreshing the nonCSC pool, which has limited proliferative potential. Expansion of larger metastases is driven primarily by proliferation of the more numerous nonCSC progeny. The observation that the CSC population plateaus early despite ongoing CSC proliferation implies a strong negative feedback mechanism in larger metastases that regulates CSC fate to limit CSC population expansion.

### Early inhibition of CSC population expansion is density dependent and driven by increased CSC differentiation

**Fig. 3:**
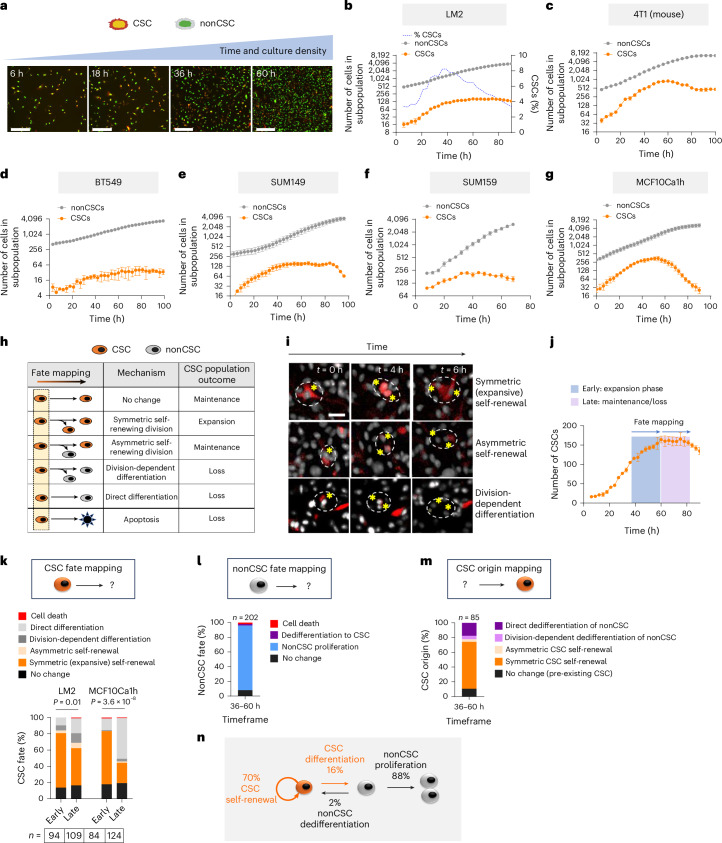
Early inhibition of CSC population expansion is driven by increased CSC differentiation. **a**, CSCs and nonCSCs in unsorted LM2 cultures in vitro with increasing time in culture. Images are representative of >100 similar experiments. Scale bar, 200 µm. **b**, Expansion kinetics for CSCs and nonCSCs in unsorted LM2 cells in vitro as a function of time. The percentage of CSCs is shown on the right *y* axis. Mean ± s.d., *n* = 3. **c**–**g**, Expansion kinetics of CSCs and nonCSCs in unsorted cultures of the additional breast cancer cell lines 4T1 (mouse) (**c**), BT549 (**d**), SUM149 (**e**), SUM159 (**f**) and MCF10Ca1h (**g**). Mean ± s.d., *n* = 3. Data in **b**, **c** and **e**–**g** represent ≥2 independent experiments. **h**, Schematic for all possible CSC cell fates in fate-mapping experiments. **i**, Time-lapse images showing different types of CSC cell division, representative of ≥20 such events. Red: cytoplasmic mCherry (CSCs); white: nuclear GFP (all tumour cells). Yellow asterisks indicate parent CSC and mitotic daughters. Scale bar, 30 µm. **j**, Timeframe for fate mapping of CSCs in relation to their expansion and maintenance/loss phase in unsorted LM2 cultures. Mean ± s.d., *n* = 3. **k**, CSC cell fates during expansion (early) phase and maintenance/loss phase (late) for LM2 and MCF10Ca1h cells. Two-sided Fisher’s exact test for self-renewal versus other fates. **l**, NonCSC cell fates in LM2 cultures in early (expansion) phase. **m**, Origin of CSCs present at *t* = 60 h (end of expansion phase) in LM2 cultures. In **l** and **m**, *n* indicates number of cells mapped. **n**, Schematic for relative frequency of major cell fate changes in LM2 cultures in the 36–60 h timeframe (expansion phase). The graphic shows the percentage of mapped CSCs or nonCSCs undergoing the indicated fate change during the 24-h period. The remaining 10% of nonCSCs and 14% of CSCs are unchanged during the mapping period. Source data

We reasoned that understanding this inhibitory feedback mechanism might help identify targetable pathways to selectively eliminate CSCs. Using Incucyte live cell imaging for simultaneous real-time monitoring of CSCs and nonCSCs (Fig. 3a), we found that the early shutdown of CSC expansion could be replicated in two-dimensional culture in vitro (Fig. 3b). The CSC population reached an early plateau while the nonCSCs in the same culture continued to expand, as seen in the metastatic lung. Consequently the percentage of CSCs in the culture is continually changing. Broadly similar kinetics were seen in other breast cancer models (Fig. 3c–g), although in some the CSC peak was followed by a loss phase.

To address what happens at the CSC plateau, we performed single-cell fate mapping. Possible CSC fates are schematized in Fig. 3h, with representative images in Fig. 3i and mapping intervals in Fig. 3j. CSC differentiation frequency increased significantly in LM2 cultures at the plateau phase, with a concomitant reduction in expansive self-renewal (Fig. 3k). The shift was more pronounced in MCF10Ca1h, which showed a net loss of CSCs in the late phase, due to a bigger increase in direct differentiation (Fig. 3k). Thus at later times in culture, CSC cell fate decisions tip from expansive self-renewal to differentiation, with reduced CSC proliferation in some but not all models.

Tumour cells show more phenotypic plasticity than their normal counterparts^1,13^. To assess whether nonCSC dedifferentiation also contributes to CSC population expansion, we fate-mapped LM2 nonCSCs. The spontaneous nonCSC dedifferentiation frequency was 2 in 100 cells per 24 h (Fig. 3l), compared with a CSC differentiation frequency of 16 in 100 cells (Fig. 3k). Thus, the forward conversion frequency of CSC to nonCSC is ~8× higher than the reverse conversion of nonCSC to CSC. However, because nonCSCs are more numerous, their low dedifferentiation frequency can still contribute substantially to CSC population expansion. Origin mapping of CSCs present at *t* = 60 h showed that 22% were generated by dedifferentiation of nonCSCs (Fig. 3m). Thus, in this aggressive TNBC model, nonCSC plasticity helps drive CSC population expansion. The relative frequency of the major CSC and nonCSC fate changes in the LM2 expansion phase is schematized in Fig. 3n.

### CSCs are hyper-responsive to microenvironmental inputs

**Fig. 4:**
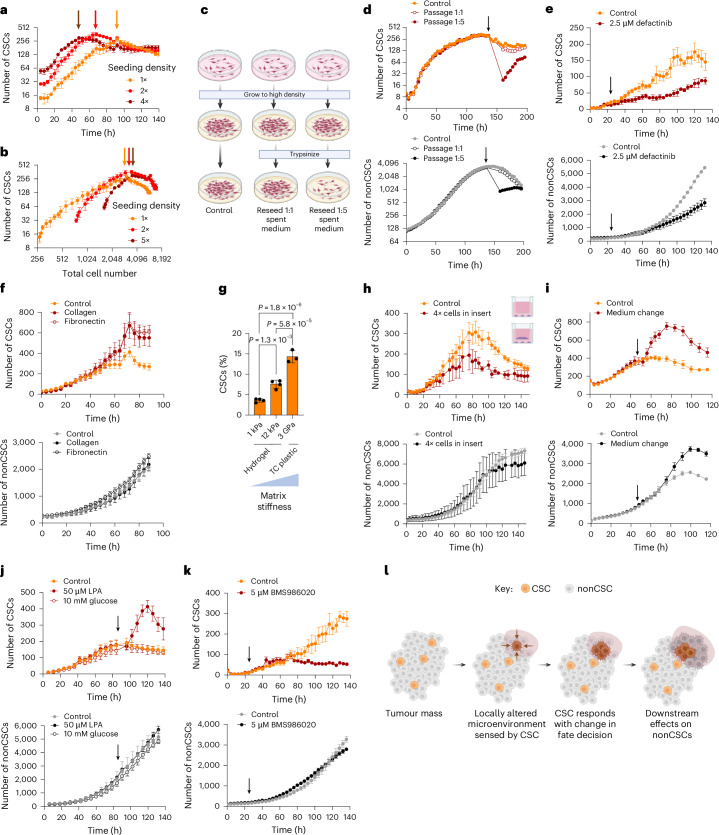
CSCs are hyper-responsive to microenvironmental inputs. **a**, Effect of initial seeding density on LM2 CSC expansion kinetics as a function of time in unsorted cultures containing both CSCs and nonCSCs. Arrows indicate peak CSC numbers. Mean ± s.d., *n* = 3 per timepoint. Data are representative of independent experiments with two different cell lines. **b**, Data from **a** replotted as a function of culture density (total cells = CSCs + nonCSCs). **c**,**d**, Experimental design (**c**) and effect (**d**) of relief of cell crowding on CSC (orange/red) or nonCSC (grey/black) population dynamics in unsorted LM2 cultures. Arrow indicates time of reseeding. **e**, Effect of integrin signalling blockade with FAK inhibitor (defactinib) on LM2 CSC and nonCSC dynamics. Arrow indicates time of inhibitor addition. **f**, Effect of collagen or fibronectin coating of well on LM2 CSC and nonCSC dynamics. **g**, Effect of matrix stiffness on relative CSC numbers by flow cytometry. Mean ± s.d. for *n* = 4 (1 kPa and 12 kPa) or *n* = 3 (3 GPa). Adjusted *P* values from Tukey’s multiple comparisons test. **h**, Contact-independent effects of additional high-density LM2 cells cultured in a well insert on the expansion of LM2 CSCs and non-CSCs. **i**, Effect on LM2 cells of replacing spent medium with fresh medium at 48 h (arrow). **j**, Effect of LPA or glucose addition at 90 h (arrow) on LM2 cultures. **k**, Effect of LPAR inhibitor BMS986020 addition at 24 h (arrow) to LM2 cultures. In **a**–**f** and **h**–**k**, data for CSCs and nonCSCs in these mixed cultures are plotted separately for clarity. Results are mean ± s.d., *n* = 3 replicates. In **a**–**k**, results are representative of at least two independent replicate experiments. **l**, Model for role of CSC as a microenvironmental sensor for the tumour cell mass. Schematics created in BioRender: **c**, Wakefield, L. https://biorender.com/dr7tj94 (2026); **h**, Wakefield, L. https://biorender.com/l56dlm1 (2026); **l**, Wakefield, L. https://biorender.com/sj1jd9c (2026). Source data

**Extended Data Fig. 3:**
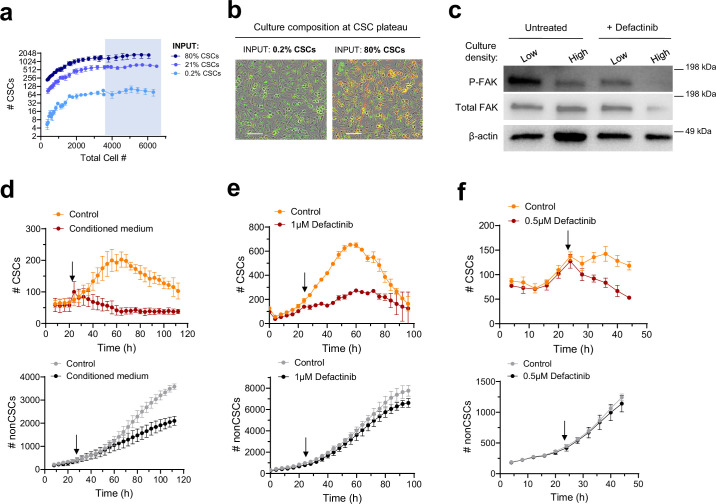
Culture density-dependent inputs differentially affect CSCs and nonCSCs. **a**. CSC expansion kinetics as a function of culture density for FACS-sorted LM2 cell populations with different starting % CSCs. Shaded area indicates CSC plateau. Mean +/- SD, n = 3. **b**. Images of culture composition at time of peak CSC plateau for LM2 cultures with different input % CSCs as indicated (images from experiment in **(a)**). Images are representative of ≥20 for each condition. Scale bar, 100 µm **c**. Western blot showing lower FAK activation at high cell density or on treatment with FAK inhibitor in LM2 cultures. Data from a single experiment. **d**. Effect on LM2 CSCs and nonCSCs of switching medium at 24 h to medium preconditioned by high density LM2 cultures. **e,f**. Effect of FAK inhibitor defactinib on CSC and nonCSCs in 4T1 (**e**) and SUM159 (**f**) cultures. Results in **d-f** are from unsorted cultures but with CSCs and nonCSCs plotted separately for clarity. Arrows indicate time of intervention. Mean +/-SD for n = 3. Results in **d-f** are all representative of at least two independent experiments. Numerical source data are provided in the Source Data file. Source data

We next addressed possible mechanisms driving the negative feedback and fate decision changes at the CSC plateau. With higher initial seeding densities, the CSC population peaked earlier in time, but when plotted against total cell number, it always peaked at the same cell density (Fig. 4a,b), suggesting that some feature of a dense culture governs CSC fate decisions. Although our initial hypothesis was that the expanding nonCSC population might feedback inhibit the CSCs, CSC numbers plateaued at the same total cell density regardless of culture composition (CSC versus nonCSC) (Extended Data Fig. 3a,b).

Increasing culture density entails many microenvironmental changes, including increased cell–cell contact and crowding, reduced cell spreading and matrix interaction, nutrient depletion and build-up of inhibitory factors. To address the contribution of cell crowding, we took LM2 cells in CSC plateau phase, removed the spent medium, trypsinized the cells and reseeded them into the same wells at the original density (1-in-1) or at a 1-in-5 split ratio, in the presence of the spent medium (Fig. 4c). The 1-in-5 split led to a rapid expansion of the CSC population, while reseeding at the original cell density had no effect (Fig. 4d). NonCSCs in the same culture responded far less to low-density reseeding (Fig. 4d), suggesting that relief of cell crowding can selectively promote CSC population expansion.

Cell crowding decreases extracellular matrix (ECM) contact and reduces integrin-driven activation of FAK/SRC signalling^19,20^ (Extended Data Fig. 3c). Consistent with a positive role for integrin signalling in CSC self-renewal, the FAK inhibitor defactinib slowed CSC population expansion earlier and more extensively than nonCSC expansion (Fig. 4e). Conversely, plating cells on collagen or fibronectin to stimulate integrin signalling selectively increased the CSC population (Fig. 4f), and the percentage of CSCs also increased with matrix stiffness (Fig. 4g), consistent with prior observations^21^. Thus CSCs show a heightened sensitivity to biochemical and biomechanical features of their microenvironment.

Culture density effects that were independent of cell contact were also observed. Tumour cells in a Transwell insert decreased peak CSC numbers in the well below, with a much smaller effect on nonCSCs (Fig. 4h), implying a contact-independent inhibitory signal. Medium refreshment at the CSC plateau caused a transient increase in expansive CSC self-renewal that was faster and larger than any effect on nonCSCs (Fig. 4i), while conditioned medium from high-density cultures caused CSC loss (Extended Data Fig. 3d). The stimulatory effect of medium refreshment on LM2 CSCs could be mimicked by lysophosphatidic acid (LPA), a serum mitogen^22^, but not by additional glucose (Fig. 4j), and CSC expansion was blocked by the LPA receptor inhibitor BMS986020 (Fig. 4k).

In every perturbation tested, the response of CSCs was more extensive and/or faster than the nonCSC response (Fig. 4d–k), leading us to suggest that CSCs serve as sensitive sensors of microenvironmental quality for the tumour cell population as a whole (Fig. 4l). In this model, CSCs continuously evaluate their local microenvironment and calibrate their numbers in response to available resources and space by altering the balance between self-renewal and differentiation. Because nonCSCs derive from CSCs, these rapid CSC responses ultimately drive downstream changes in the nonCSC population, but this will occur with a temporal lag, as most nonCSCs have some residual proliferative capacity and nonCSC proliferation is less immediately affected by environmental conditions. Similar results were seen with 4T1 or SUM159 cells (shown for FAK inhibition in Extended Data Fig. 3e,f).

### Multiple CSC sensor inputs converge on YAP/TAZ

**Fig. 5:**
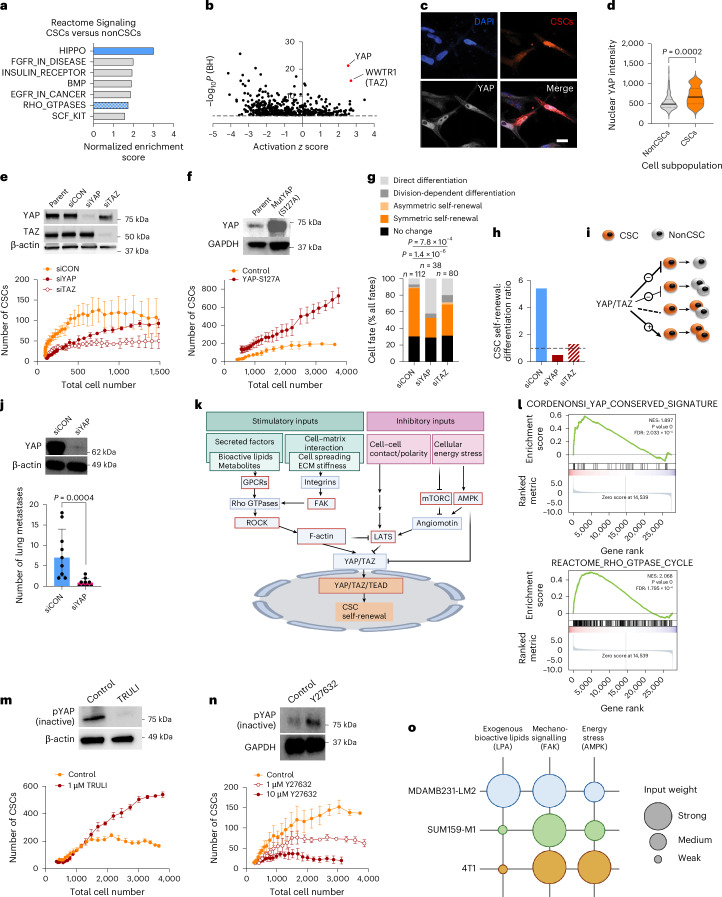
Multiple microenvironmental inputs to the CSC sensor converge on YAP/TAZ. **a**, Top Reactome Signaling pathways enriched in CSC versus nonCSC transcriptomes from sorted LM2 cells in vitro. **b**, Ingenuity Pathway Analysis showing predicted upstream regulators of the LM2 CSC transcriptome in vitro. *P* values for overlap were calculated using a one-sided Fisher’s exact test with Benjamini–Hochberg (BH) correction for multiple hypothesis testing. **c**,**d**, Multiplexed immunofluorescence (**c**) and quantitation (**d**) of nuclear YAP intensity in CSCs and nonCSCs in unsorted LM2 low-density culture. Image representative of ≥20 similar fields. Scale bar, 5 µm. *n* = 24 cells (CSCs) and *n* = 493 cells (nonCSCs); two-sided Mann–Whitney test. **e**, Effect of siRNA knockdown of YAP or TAZ on CSC dynamics in unsorted LM2 cultures. Note that results are plotted on the cell density axis (total number of cells). Mean ± s.d., *n* = 3. Results replicated in an independent experiment using shRNA knockdown. **f**, Effect of overexpression of constitutively active mutant YAP-S127A on CSC dynamics in unsorted LM2 cultures. Mean ± s.d., *n* = 3. **g**, Single-cell fate mapping of LM2 CSCs following YAP or TAZ knockdown. Two-sided Fisher’s exact test versus control for all self-renewal versus all differentiation. *n* indicates the number of cells assessed. **h**, Effect of YAP/TAZ knockdown on CSC self-renewal:differentiation ratio in LM2 cells (data from **g**). The horizontal dashed line indicates balanced self-renewal and differentiation. **i**, Schematic summarizing effect of YAP on CSC fates. Dashed line: event numbers too small to determine effect. **j**, Effect of stable YAP knockdown on metastatic efficiency of LM2 cells. Median ± interquartile range for *n* = 9 mice per group; two-sided Mann–Whitney *U* test. **k**, Schematic of upstream inputs to YAP/TAZ node. Red outlines indicate pathway components targeted by inhibitors in this study. **l**, Gene set enrichment plots for YAP signature and Rho-GTPase signalling in CSCs recovered from LM2 lung metastases in vivo. NES, normalized enrichment score; FDR, false discovery rate. **m**,**n**, Effect of LATS kinase inhibitor TRULI (**m**) or ROCK inhibitor Y27632 (**n**) on CSC dynamics in unsorted LM2 cultures. Mean ± s.d., *n* = 3. Western blots show effect of inhibitors on phospho-YAP-S127 (inactive form of YAP). **o**, Bubble plot summarizing relative weight of select input pathways across three TNBC models, as assessed by response to inhibitors (see Extended Data Fig. 5a for original plots). The plots in **e**, **f**, **m** and **n** are representative of at least two independent experiments. Schematic in **k** created in BioRender; Wakefield, L. https://biorender.com/gg1wwwlv (2026). Source data

**Extended Data Fig. 4:**
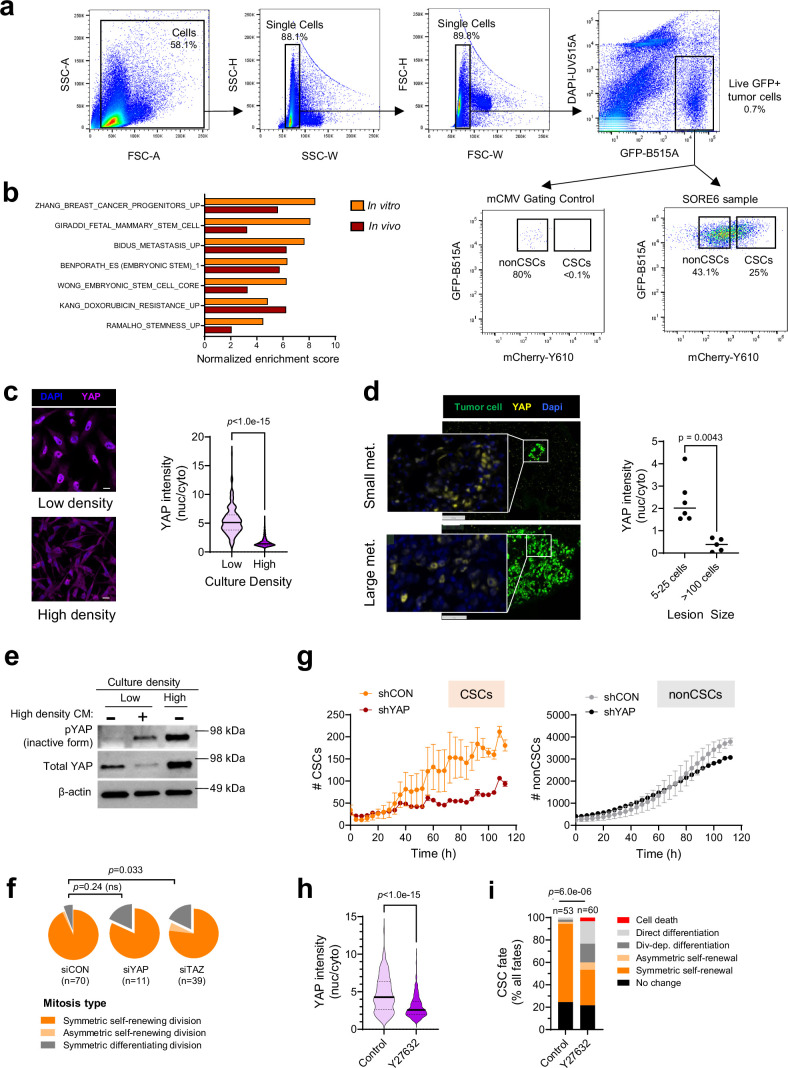
Multiple microenvironmental inputs to the CSC sensor converge on YAP/TAZ. **a**. Digested lungs from mice bearing LM2 metastases were sorted with successive gating on single cells, live GFP+ cells (all tumor cells) and mCherry+ CSCs or mCherry- nonCSCs. The mCherry CSC gate was set using parallel samples from mice injected with LM2 cells expressing the mCMV>dsmCherry control construct. **b**. Enrichment of genesets relating to stemness in the transcriptomes of FACS-sorted LM2 CSCs compared with nonCSCs, from *in vitro cultures* or recovered from metastatic lungs as indicated. **c**. Immunofluorescent staining to assess YAP localization in low and high density LM2 cultures. Images are representative of hundreds of similar fields. Scale bar: 10 µm. Results are from n = 197 cells (low density culture) or n = 1220 cells (high density culture). Solid line shows median and dotted lines interquartile ranges; Mann-Whitney test. **d**. Immunofluorescent images and quantitation of YAP localization in tumor cells in small and large LM2 lung metastases. Scale bar: 100 µm. Images are representative of >5 metastases/size bin pooled from 2 mice. Nuclear localization indicates active YAP. Line on graph shows median for 6 (small) or 5 (large) metastatic lesions per size bin. Two-sided Mann-Whitney test. **e**. Western blot of YAP phosphorylation status in response to indicated culture conditions. CM indicates treatment with conditioned medium from high density culture. Phospho-YAP is the inactive form. **f**. Proportion of different division-dependent events following YAP/TAZ knockdown in LM2 cells. n=total number of proliferation events tracked. Two-sided Fisher Exact test for symmetric self-renewing mitoses. **g**. CSC and nonCSC expansion kinetics following shYAP knockdown in LM2 cells. Results are mean +/- SD for n = 6 in a single experiment. **h**. Quantitation of YAP localization by immunofluorescence in LM2 cultures following treatment with the ROCK inhibitor Y27632. n = 330 (Con) and n = 400 (Y27632); Two-sided Mann-Whitney test **i**. Single cell fate mapping showing effect of ROCK inhibition on CSC fate in LM2 cultures. Two-sided Fisher Exact test for self-renewal vs other fates. n = number of cells assessed. Numerical source data are provided in the Source Data file. Source data

To identify underlying molecular mechanisms, we performed RNA sequencing on CSCs and nonCSCs sorted from LM2 in vitro cultures or metastasis-bearing lungs (Extended Data Fig. 4a). The CSC transcriptome showed strong enrichment of stemness, drug resistance and metastasis gene signatures (Extended Data Fig. 4b). Gene set enrichment analysis identified the Hippo pathway as the most highly enriched Reactome Signaling pathway in the CSCs in vitro (Fig. 5a), and ingenuity pathway analysis identified YAP and TAZ (*WWTR1* gene) as the most significant positive upstream regulators of the CSC transcriptome (Fig. 5b).

YAP and TAZ constitute a key nodal point in the transduction of biomechanical and other extracellular cues that regulate organ size and cell fate decisions^20,23,24^. Previously implicated in regulating stemness in breast and other cancers^20,23^, they are plausible integrators of inputs to the CSC in its role as a microenvironment sensor. Activation of YAP and TAZ involves their stabilization and translocation to the nucleus^24^, where they interact with TEADs to modulate transcription^25^. We showed preferential localization of YAP/TAZ in the nuclei of LM2 cells in low-density versus high-density cultures in vitro (Extended Data Fig. 4c), and in small versus large metastases in vivo (Extended Data Fig. 4d), as expected from known inhibitory effects of cell crowding on YAP/TAZ^23^. Western blots confirmed YAP inactivation in high-density LM2 cultures, and in low-density cultures treated with high-density culture conditioned medium (Extended Data Fig. 4e). Importantly, CSCs had more nuclear YAP than nonCSCs (Fig. 5c,d), confirming preferential YAP activation in CSCs.

Addressing their functional role, small interfering RNA (siRNA) knockdown of YAP or TAZ decreased CSC population expansion (Fig. 5e), while overexpression of constitutively active YAP-S127A increased CSCs and overrode the density inhibition (Fig. 5f). Because YAP and TAZ can affect cell proliferation independently from cell fate decisions^23^, the data are plotted against total cell number to deconvolute direct effects on CSCs from effects secondary to changes in culture density. Fate mapping showed that YAP or TAZ knockdown reduced CSCs mainly by decreasing expansive self-renewal and increasing differentiation (Fig. 5g,h), through more differentiating divisions (Extended Data Fig. 4f), and a big increase in direct differentiation (Fig. 5g). YAP/TAZ effects on CSC fates are summarized in Fig. 5i. Stable short hairpin RNA (shRNA) knockdown of YAP in LM2 cells selectively decreased CSCs two- to threefold in vitro (Extended Data Fig. 4g), and significantly decreased metastatic efficiency (Fig. 5j), confirming a key role for YAP in maintaining and expanding the CSC population during metastasis.

Multiple stimulatory and inhibitory microenvironmental inputs converge on YAP/TAZ^20,23,24^ (Fig. 5k). The Hippo pathway is the canonical negative regulator with LATS kinases as key mediators^26^, suppressing YAP/TAZ at high cell density^24^, and in response to cellular energy stresses^27^. On the stimulatory side, Rho-GTPases are particularly important positive upstream regulators^28^, and we found Rho-GTPase activity to be preferentially enriched along with YAP activity in the transcriptome of LM2 CSCs in vitro (Fig. 5a) and in vivo (Fig. 5l). Key metabolites such as serum lipids, fatty acids, glutamate and purines can activate Rho-GTPases and the effector kinase ROCK to regulate YAP/TAZ^27^. ROCK also plays a key role in mechanotransduction, mediating signalling from integrins to activate YAP/TAZ^26^. Consistent with this regulatory architecture, the LATS inhibitor TRULI blocked YAP inactivation and enabled continued expansion of CSCs at high density (Fig. 5m), while the ROCK inhibitor Y27632 inactivated YAP and reduced CSC expansion, by increasing CSC differentiation and reducing self-renewal (Fig. 5n and Extended Data Fig. 4h,i). Thus, both Hippo and Rho GTPase signalling were confirmed as upstream regulators of CSC dynamics through modulation of YAP/TAZ.

**Extended Data Fig. 5:**
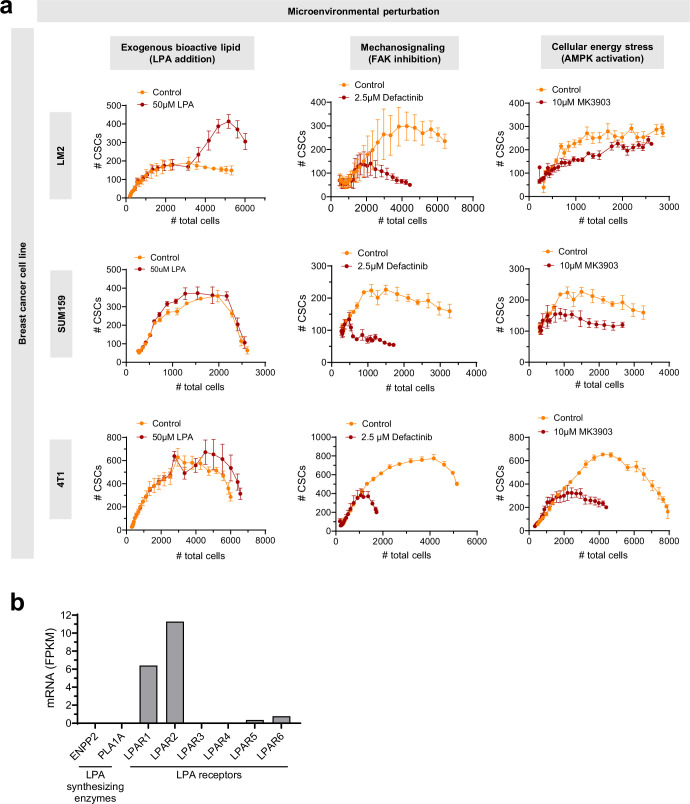
Different inputs to the CSC sensor dominate in different breast cancer models. **a**. Effect of indicated microenvironmental perturbations on CSC dynamics in different breast cancer cell lines. CSC numbers are plotted as a function of culture density (total cells) to disambiguate direct effects of intervention on CSCs from secondary effects due to changes in culture density. Mean +/- SD for n = 3. Results are representative of at least 2 independent experiments. **b**. LPA pathway transcript expression in unsorted LM2 cultures *in vitro* of LPA biosynthesis and response genes. Data from a single experiment. Numerical source data are provided in the Source Data file. Source data

Testing inhibitors of key upstream nodes (Fig. 5k, red boxes) across three models of metastatic TNBC showed that all inputs influenced CSC self-renewal but the dominant input varied between models (Fig. 5o, Extended Data Fig. 5a and Supplementary Table 1). LM2 cells were particularly sensitive to exogenous bioactive lipids, probably because the LPA synthesizing enzymes autotaxin (ENPP2) and phospholipase A1 (PLA1A)^22^ are not expressed (Extended Data Fig. 5b). All three lines were sensitive to modulation of mechanosensing by FAK inhibition, while 4T1s were particularly sensitive to energy stress induced by AMPK activation. Thus, the YAP/TAZ node in CSCs integrates information from multiple microenvironmental inputs, with relative weight varying between models.

### CSC hyper-responsiveness involves amplified input signals and a more TEAD-accessible chromatin organization

**Fig. 6:**
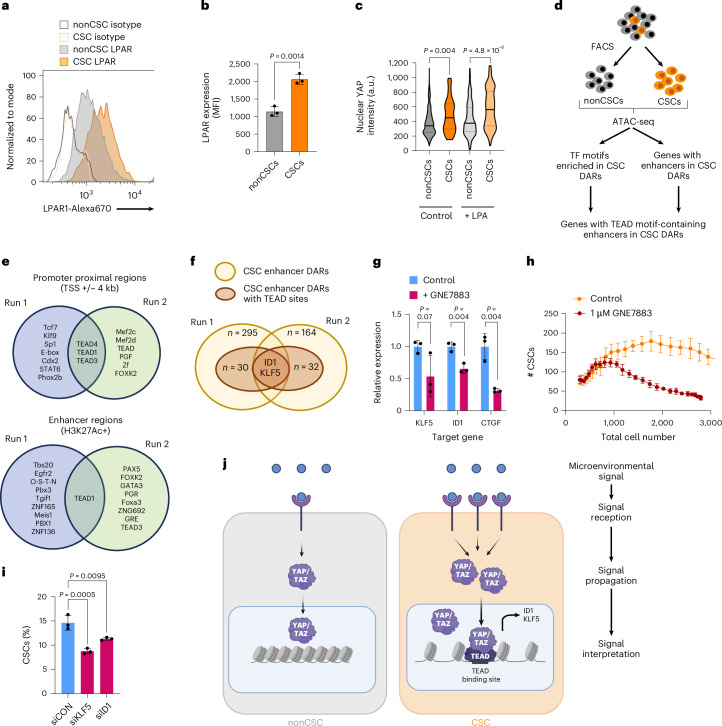
CSC hyper-responsiveness to LPA involves amplified signalling and more TEAD-accessible chromatin. **a**, Flow cytometry for LPAR1 expression in LM2 CSCs (mCherry^pos^) versus nonCSCs (mCherry^neg^). **b**, Quantitation of data in **a**. Mean ± s.d., *n* = 3. Two-sided unpaired *t*-test. Representative of two independent experiments. MFI, median fluorescence intensity. **c**, Immunofluorescent quantitation of YAP localization in LM2 CSCs versus nonCSCs in unsorted cultures with and without treatment with LPA. *n* = 524 and 400 for nonCSCs ± LPA; *n* = 55 and 80 for CSCs ± LPA. Violin plots show medians (solid lines) with interquartile range (dotted lines). Two-sided Kruskal–Wallis test for CSCs versus nonCSCs within treatment group, with Dunn’s multiple comparisons correction. Data pooled from two biological replicates. **d**, Schematic for the ATAC-seq strategy. **e**, Top ten enriched transcription factor (TF) binding motifs in DARs in sorted LM2 CSCs versus nonCSCs around TSSs or enhancer regions for two independent ATAC-seq experiments (run 2 had only nine significantly enriched motifs). The two-tailed *P*-value cut-off for motif enrichment from cumulative binomial distribution test was 0.01. The exact *P* values and Benjamini–Hochberg *q* values are given in the Source Data. O-S-T-N, OCT4–SOX2–TCF–NANOG. **f**, Genes with TEAD binding sites in enhancers that are preferentially accessible in CSCs in both of two independent experiments (see the Source Data for lists of all annotated genes with CSC enhancer DARs with and without TEAD sites and corresponding *P* values). The number of DARs in each major category is indicated. **g**, Effect of TEAD inhibitor GNE7883 treatment on expression of KLF5 and ID1 in LM2 cells. CTGF is a canonical YAP target as positive control. Mean ± s.d. for *n* = 3, normalized to vehicle control. Two-sided Student’s *t*-test. **h**, Effect of GNE7883 on CSC dynamics in unsorted LM2 cultures. Mean ± s.d., *n* = 3, representative of two independent experiments. **i**, Flow cytometry for effect of 1D1 or KLF5 knockdown on the percentage of CSCs in LM2 cultures. Mean ± s.d. for *n* = 3, Dunnett’s multiple comparisons test versus control (CON). **j**, Schematic for mechanisms underlying the hypersensitivity of CSCs to microenvironmental signals. Schematic in **j** created in BioRender; Wakefield, L. https://biorender.com/uv47yib (2026). Source data

The greater responsiveness of CSCs to microenvironmental signals could involve more sensitive signal detection leading to more active YAP/TAZ (input arm), or more efficient conversion of YAP/TAZ activation into phenotypic output (effector arm). Using LPA as a representative stimulus in LM2 cells, we showed that CSCs express more LPA receptor1 (LPAR1) on the cell surface (Fig. 6a,b), and LPA treatment increased nuclear YAP/TAZ to a greater extent in CSCs than nonCSCs (Fig. 6c), indicating that LPA generates an amplified signal in CSCs.

To address the effector arm, we performed assays for transposase-accessible chromatin using sequencing (ATAC-seq) on sorted LM2 CSCs and nonCSCs (Fig. 6d). There were no consistent global chromatin accessibility differences, but each subpopulation had some local differentially accessible regions (DARs). Focusing on CSC DARs in promoter proximal regions (transcriptional start site (TSS) ± 4 kb) and in previously identified MDAMB231 enhancers^29^, we searched for enriched transcription factor binding motifs. TEAD motifs were consistently enriched in CSC promoter and enhancer DARs in each of two independent experiments, while other motifs were more variable (Fig. 6e). Because >90% of YAP/TAZ/TEAD binding occurs at distal active enhancer regions (marked by H3K27Ac)^25,30^, we focused on CSC enhancer DARs with TEAD binding sites. The two genes consistently associated with such enhancers (Fig. 6f) were ID1, a well-characterized inhibitor of differentiation^31,32^, and KLF5, a key transcription factor in the maintenance of the stem state^33^. TEAD inhibition with GNE7883^34^ reduced ID1 and KLF5 mRNAs (Fig. 6g) and decreased CSCs in LM2 cultures (Fig. 6h). Knockdown of ID1 or KLF5 with siRNA also reduced CSCs (Fig. 6i), consistent with TEAD-dependent expression of both proteins maintaining stemness.

**Extended Data Fig. 6:**
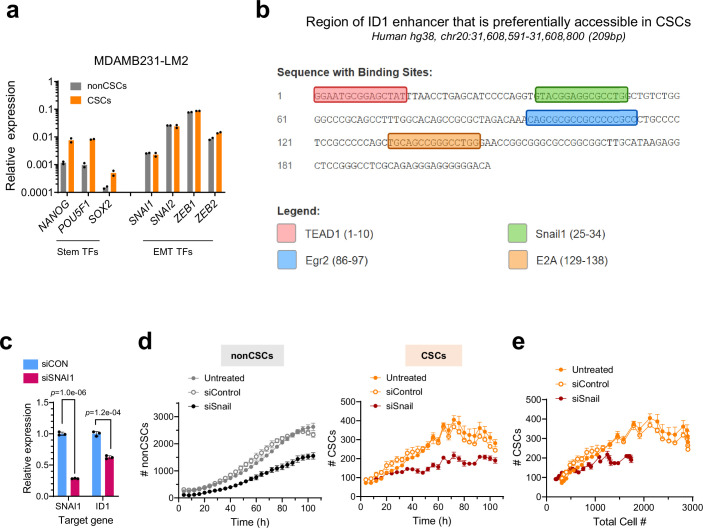
Role of EMT transcription factors in CSC expansion. **a**. RTQ-PCR assessment of transcription factor mRNA expression in FACS-sorted LM2 CSCs and nonCSCs. Bars are mean of n = 2 replicates from a single experiment. **b**. Transcription factor binding sites in the preferentially accessible region in the ID1 gene enhancer in CSCs **c**. Effect of Snai1 knockdown on ID1 mRNA in LM2 cells. Mean +/- SD, n = 3. Two-sided unpaired t-test. **d**. Effect of Snail1 knockdown on LM2 nonCSC and CSC kinetics (from same culture but plotted separately for clarity). Mean +/- SD, n = 3. **e**. Effect of Snail knockdown on CSC expansion in LM2 cells as a function of culture density. Mean +/- SD, n = 3. Numerical source data are provided in the Source Data file. Source data

Important interrelationships exist between YAP/TAZ/TEAD, stemness and the epithelial-to-mesenchymal transition (EMT). YAP/TAZ cooperate with oncogenic KRas to promote EMT^35^, and inducers of EMT or forced overexpression of core EMT transcription factors (EMT-TFs) increase the frequency of stem-like cells in cancer cell populations^36^. Furthermore, cells that have undergone a partial EMT have increased tumour and metastasis initiating ability^37,38^. While the stemness transcription factors SOX2, OCT4 (*POU5F1* gene) and NANOG were strongly enriched in our CSC population, classic EMT transcription factors were not (Extended Data Fig. 6a). However, the CSC enhancer DAR of the ID1 gene has a Snail (SNAI1) binding site near the TEAD site (Extended Data Fig. 6b). SNAI1 knockdown reduced ID1 mRNA by ~30%, and suppressed CSC more than nonCSC expansion (Extended Data Fig. 6c–e), suggesting that EMT-TFs may functionally contribute to TEAD-mediated regulation of stemness.

Our data indicate that CSC hyper-responsiveness to microenvironmental change reflects enhanced reception and propagation of microenvironmental signals, as well as enhanced signal interpretation through an altered chromatin architecture that increases TEAD binding site accessibility at regulatory elements of genes that inhibit differentiation and sustain stemness (Fig. 6j).

### Targeting CSC sensor nodes reduces chemotherapy-driven enrichment of CSCs in early metastasis

**Fig. 7:**
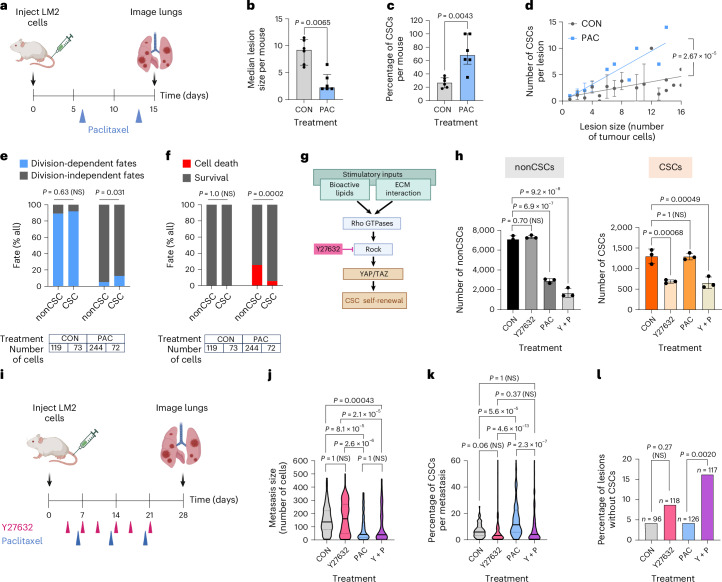
Targeting CSC sensor nodes reduces chemotherapy-driven enrichment of CSCs in metastasis. **a**, Schematic for paclitaxel treatment. **b**,**c**, Effect of paclitaxel on LM2 lung metastasis size (**b**) and percentage of CSCs among tumour cells in the lung (**c**). Median ± interquartile range, *n* = 6–7; two-sided Mann–Whitney test. **d**, Number of CSCs per lesion as a function of LM2 lung lesion size in response to paclitaxel. *n* = 1–10 lesions per size bin. Data for lesion sizes with *n* ≥ 3 datapoints are plotted as mean ± s.d. Two-sided analysis of covariance test for difference in slope. **e**,**f**, Effect of paclitaxel on division-dependent fates (**e**) and cell death (**f**) of LM2 cells in culture from single-cell fate mapping starting after 32 h of paclitaxel treatment. Two-sided Fisher’s exact test. **g**, CSC sensor inputs converging on ROCK. **h**, Effect of combined paclitaxel + ROCK inhibitor treatment for 3 days on CSCs and nonCSCs in unsorted LM2 cultures in vitro assessed by flow cytometry. Mean ± s.d. for *n* = 3, two-sided Dunnett’s multiple comparison test versus CON. **i**, Schematic for paclitaxel + Y27632 treatment in LM2 model. **j**,**k**, Effect of therapeutic interventions on lung metastasis size (**j**) or percentage of CSCs per metastasis (**k**) at endpoint. Median ± interquartile range for 96–118 metastases per group pooled from *n* = 5–6 mice per group; two-sided Dunn’s multiple comparisons test for all pairwise combinations. **l**, Treatment effect on the percentage of lesions with no CSCs. Two-sided Fisher’s exact test for indicated treatment pairs. *n* indicates the total lesion number as indicated. NS, not significant. Schematics created in BioRender: **a**, Wakefield, L. https://biorender.com/2pcq816 (2026); **g**, Wakefield, L. https://biorender.com/urhbrdb (2026); **i**, Wakefield, L. https://biorender.com/z10b5so (2026). Source data

**Extended Data Fig. 7:**
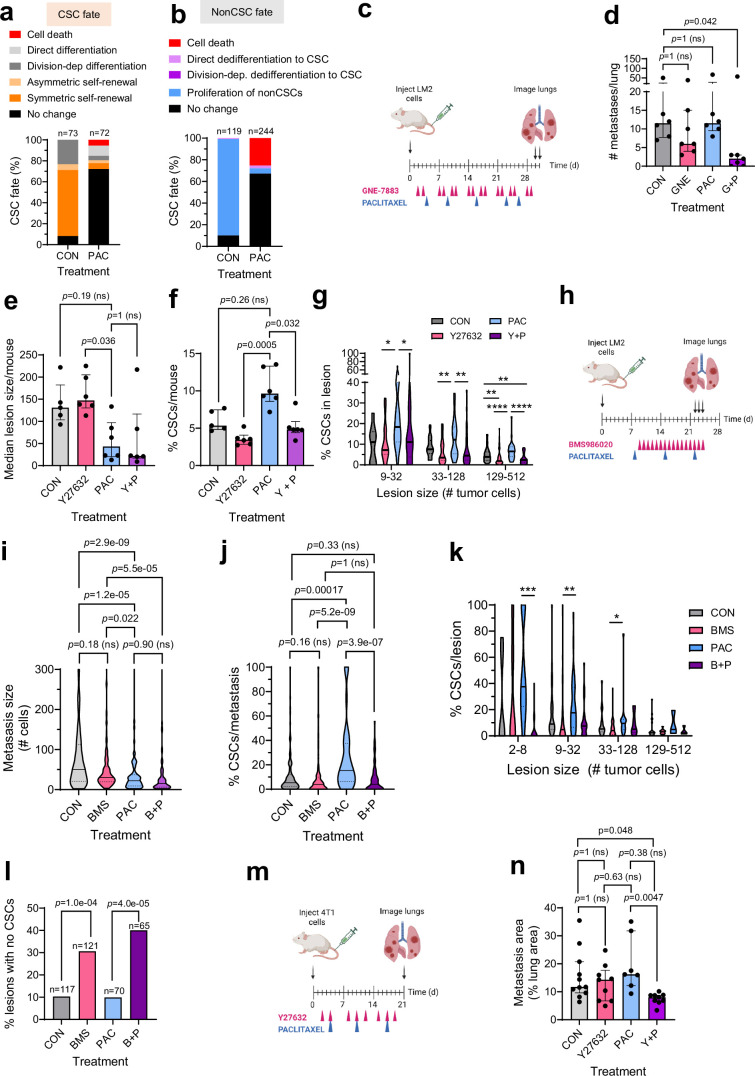
Targeting CSC sensor nodes reduces chemotherapy-driven enrichment of CSCs in metastasis. **a,b** Fate mapping of CSC **(a)** and nonCSC **(b)** responses to paclitaxel in LM2 cells *in vitro*. Detailed statistics are given in statistical source data file. **c**. Schematic for treatment with GNE7883 and paclitaxel in the LM2 model. **d**. Effect of TEAD inhibitor GNE7883 (GNE) and paclitaxel (PAC) on metastasis number in the LM2 model. Median +/- IQ range, for n = 7 mice/group; two-sided Dunn’s multiple comparison test vs CON. **e,f**. Effect of therapeutic interventions on median lung metastasis size/mouse (**e**) or % CSCs/mouse lung (**f**) at endpoint. Results are median +/- IQ range for n = 5-6 mice/group; two-sided Dunn’s multiple comparisons test vs PAC. **g**. Treatment effect on % CSCS as a function of lesion size. Data pooled from 5-6 mice/group, n = 8-70 lesions/size bin; two-sided Dunn’s multiple comparisons test for all pairwise comparisons within given size bins. Only significant differences shown. *, p < 0.05; **, p < 0.01; ***, p < 0.001; ****, p < 0.0001. Exact p-values are given in the Source data file. **h**. Schematic for treatment of LM2 model with LPAR inhibitor BMS986020 (BMS) and Paclitaxel (PAC). **i,j**. Metastasis size (# tumor cells) in lungs from mice treated Effect of treatment with LPAR inhibitor (BMS) and paclitaxel (PAC) on lung metastasis size (**i)**, or % CSCs/metastasis (**j**). Results are median +/- IQ range for 65-121 metastases/group pooled from n = 6 mice/group; two-sided Dunn’s multiple comparisons test for all pairwise combinations. **k**. Treatment effect on % CSCs/lesion as a function of metastatic lesion size. Data pooled across all mice/treatment group, n = 6 mice/group; 3-58 lesions/size bin/treatment. Two-sided Dunn’s multiple comparisons test within each size bin. Only significant differences shown. *, p < 0.05; **, p < 0.01; ***, p < 0.001; ****, p < 0.0001. Exact p-values are given in the Source data file. **l**. Effect of treatment on % metastatic lesions with no CSCs. Data pooled across all mice/treatment group, n = 6 mice/group; total lesion numbers per treatment group indicated on graph. Two-sided Fisher exact test for CON v BMS, and PAC v PAC + BMS. **m**. Schematic for treatment of 4T1 models with ROCK inhibitor Y27632 and Paclitaxel. **n**. Effect of therapeutic intervention on lung metastasis in 4T1 model in immunocompetent BALB/c mice. Results are median +/- interquartile range for n = 7-11 mice/group; two-sided Dunn’s multiple comparisons test for all pairwise comparisons. Numerical source data are provided in the Source Data file. Schematics created in BioRender: **c**, Wakefield, L. https://biorender.com/gm18r2q (2026); **h**, Wakefield, L. https://biorender.com/1vkpx24 (2026); **m**, Wakefield, L. https://biorender.com/1lhxwem (2026). Source data

Finally, we addressed therapeutic implications. CSCs are relatively resistant to therapy^9,39^, and breast tumour tissue remaining after endocrine therapy or chemotherapy is enriched for stem features^40^. Paclitaxel treatment of the LM2 model significantly reduced metastatic lesion size (Fig. 7a,b) but increased the proportion of CSCs among surviving cells across all lesion sizes (Fig. 7c,d). Fate mapping in vitro showed that paclitaxel greatly reduced all division-dependent events in both subpopulations, although CSCs were less affected than nonCSCs (Fig. 7e). Strikingly, paclitaxel induced nearly fivefold more cell death in nonCSCs than CSCs (Fig. 7f). CSC differentiation and nonCSC dedifferentiation frequencies were relatively unaffected, although CSC differentiation became mostly division independent (Extended Data Fig. 7a,b). Overall, CSCs maintain some self-renewal capacity under paclitaxel treatment and show enhanced survival relative to nonCSCs.

We next asked whether blocking CSC self-renewal could reverse chemotherapy-induced CSC enrichment. In principle, the YAP/TAZ/TEAD node would be the optimal target, and promising TEAD inhibitors are being developed^34,41^. The TEAD inhibitor GNE7883^34^ combined with paclitaxel significantly decreased metastasis number in the LM2 model (Extended Data Fig. 7c,d), although the recommended excipient precluded reliable CSC imaging. Instead, for proof of concept, we used Y27632 to inhibit ROCK, an upstream node for both bioactive lipid and mechanosignalling to YAP/TAZ (Fig. 7g). Flow cytometry confirmed that treatment of LM2 cells with paclitaxel alone selectively reduced nonCSCs, while Y27632 alone selectively reduced CSCs, and the combination reduced both in vitro (Fig. 7h). In vivo, ROCK inhibition did not compromise the ability of paclitaxel to reduce metastatic lesion size (Fig. 7i,j and Extended Data Fig. 7e) but did reverse the paclitaxel-induced enrichment of CSCs across all lesion sizes (Fig. 7k and Extended Data Fig. 7f,g) and increased the proportion of metastases that had no CSCs (Fig. 7l), suggesting that some metastases were being ‘sterilized’ of their CSCs. Similar results were seen with the LPAR inhibitor BMS5986020 in the LM2 model (Extended Data Fig. 7h–l). Y27632 with paclitaxel also significantly reduced metastatic burden in immunocompetent mice, using the 4T1 model without imaging markers to avoid immune rejection (Extended Data Fig. 7m,n). Thus targeting regulatory nodes in the CSC sensor mechanism combines with conventional chemotherapy in vivo to improve antimetastatic responses.

## Discussion

Direct observation of CSCs in metastatic breast cancer models using our fluorescent reporter has revealed very different population dynamics for CSCs and nonCSCs in early lung metastatic colonization. In the first few cell divisions, only CSCs divide, undergoing expansive self-renewal while also dividing asymmetrically to generate nonCSC progeny. The CSC population expansion soon ceases, however, and further metastasis growth is driven primarily by nonCSC population expansion. These divergent dynamics revealed that CSCs uniquely experience early negative feedback in growing metastases.

We anticipated that this negative feedback would involve reduced CSC proliferation, but unexpectedly found that CSCs in late-stage metastases were as proliferative as nonCSCs, despite a static CSC population size. Thus, CSC self-renewal must be balanced by CSC differentiation to nonCSCs in larger lesions, with negative feedback primarily affecting CSC fate decisions rather than proliferation. Ongoing CSC proliferation makes intuitive sense because individual nonCSCs can undergo only limited divisions before exhausting their proliferative potential^3^, requiring continual replenishment by CSCs. The high proliferative activity of CSCs in large metastases also importantly suggests that their relative resistance to antimitotic therapies relies on properties other than quiescence, which has previously been proposed as a contributing mechanism^42^.

Modelling these distinctive population dynamics in vitro, we found that CSCs responded to many features of a crowded cell environment, such as reduced matrix attachment or depleted trophic factors, with increased differentiation and reduced expansive self-renewal. Strikingly, the CSC population consistently responded more rapidly and extensively than nonCSCs to a wide variety of stimuli, leading us to the broader concept that CSCs may serve as sensitive sensors and first responders to changing local microenvironmental conditions. Because CSCs are distributed through the tumour (Fig. 1c,d), they are well positioned to serve this role. NonCSCs have limited proliferative capacity, so the CSC population expansion or contraction that occurs in response to local microenvironmental change ultimately reads out in changes in numbers of nonCSC progeny, thereby coupling local tumour expansion dynamics to resource availability and biomechanical cues. This situation has clear analogies with developmental and repair processes, where mechanisms must be in place to integrate signals from the tissue as a whole, calibrating tissue expansion to the functionally optimal size^24^. As an aside, it is worth noting that important experimental consequences of the high sensitivity of CSCs to microenvironmental inputs include (1) a strong dependence of CSC numbers and dynamics on initial conditions (‘butterfly effect’ in chaos theory), and (2) culture density as a potentially confounding variable in CSC assays and drug screens.

We found that microenvironmental inputs affecting breast CSC dynamics activated many interconnected pathways that converged on the regulatory YAP/TAZ/TEAD node to change CSC fate decisions. YAP, TAZ and TEADs respond to biomechanical, metabolic, inflammatory and other cues to modulate cell fate decisions during development and tissue repair^24^, and earlier studies had implicated them in regulating the stem phenotype in tissue regeneration and disease, including in breast cancer^13,20,23,43^. However, our finding that this node is selectively hyper-responsive in CSCs has important conceptual and practical implications for understanding CSC interactions with their changing microenvironment. Underlying mechanisms included enhanced signal reception and propagation in CSCs, and greater accessibility of TEAD binding sites in enhancers of stem cell regulatory genes. Thus, in comparison with nonCSCs, CSCs are primed to amplify responses to incoming microenvironmental signals at every level of the integrated sensor and effector network.

Our discovery of distinct population kinetics for CSCs and nonCSCs was grounded in observations made in tumour-bearing mice in vivo. Surprisingly, we were able to replicate key features in two-dimensional culture and establish the overarching principle that microenvironmental inputs impact CSCs more profoundly than nonCSCs. Mechanistic convergence on YAP/TAZ/TEAD was a consistent feature, but the relative contribution of different inputs varied among three TNBC models, probably reflecting the heterogeneous nature of the disease. Addressing this heterogeneity further, it will be important to determine if the CSC sensor concept extends to other breast cancer subtypes, and indeed to other epithelial tumours. The use of more sophisticated in vitro three-dimensional models with greater microenvironmental complexity, and the incorporation of patient-derived material, will be important in improving the predictive value of in vitro approaches. Such models with their more realistic architecture and biology should also enable delineation of additional microenvironmental inputs beyond the ones we identified here. For example, there is a complex reciprocal interplay between YAP/TAZ and metabolism that should be probed further. YAP/TAZ activity decreases when provision of growth-sustaining nutrients such as glucose and fatty acids is low, and conversely YAP/TAZ can promote glycolysis, lipogenesis and glutaminolysis under nutrient-replete conditions^27^. Similarly, the detailed composition of the ECM, its three-dimensional organization and biophysical properties will probably be important^21^. Finally, TAZ depletion in breast cancer models enhances antitumour immune responses^44^, suggesting potential interactions between CSCs, YAP/TAZ and the adaptive immune system that warrant further exploration.

The utility of our approach was demonstrated by our ability to pharmacologically target the CSC sensor mechanism to improve metastatic outcome in preclinical models. Most current therapeutics are relatively ineffective against CSCs, leading to CSC enrichment after treatment^1^. Conceptually, strategies to differentiate CSCs should prevent this phenomenon and lead to more durable responses. Pathways such as Wnt and Notch have previously been implicated in regulating CSC dynamics, but targeting these has proved challenging due to their involvement in normal tissues^45^. By contrast, the hypersensitivity of CSCs to microenvironmental signals that regulate CSC self-renewal via YAP/TAZ/TEAD presents an exploitable vulnerability, since YAP and TAZ are largely dispensable for normal adult homeostasis^20,23^.

Newly developed TEAD inhibitors are showing early promise against Hippo pathway-mutated tumours, and for circumventing resistance to targeted therapies^34,41^. While the effects of TEAD inhibition on CSCs were not directly assessed in those prior studies, the major role for CSCs in therapy resistance makes CSCs plausibly the critical cellular targets. In our hands, direct TEAD inhibition with GNE7883, or upstream blockade with LPAR or ROCK inhibitors, reduced metastatic burden when combined with paclitaxel, in part by preventing the paclitaxel-driven enrichment of CSCs. Given that the relative weight of upstream inputs to the YAP/TAZ/TEAD node varies among tumour models, TEAD inhibitors should be the most broadly applicable therapeutic strategy, if safety profiles are tolerable. However, a bet-hedging strategy of simultaneously targeting multiple upstream input nodes is worth further investigation. A potential advantage of a low-dose multitarget approach would be divided toxicity and reduced activation of bypass mechanisms, as seen with a multidrug strategy to overcome variations in MAPK network topology^46^. Finally, because the efficacy of any differentiating therapy will be limited by nonCSC dedifferentiation, therapeutics that induce CSC differentiation and simultaneously block nonCSC plasticity would be optimal. Our dynamic reporter with its ability to report on both processes should facilitate the quest for such agents.

## Methods

### Ethics statement

Our research complies with all relevant ethical regulations. All animal procedures were performed by National Cancer Institute – Center for Cancer Research (NCI-CCR) affiliated staff, approved by the NCI Animal Care and Use Committee and in accordance with federal regulatory requirements and standards. All components of the intramural National Institutes of Health Animal Care and Use Committee (NIH ACUC) programme are accredited by the Association for Assessment and Accreditation of Laboratory Animal Care (AAALAC) International.

### Cell lines and cell culture

The origin, providers and culture media for breast cancer cell lines used in this study were as follows: MDAMB231 (human TNBC); American Type Culture Collection; cultured in Dulbecco’s modified Eagle medium (DMEM)/10% fetal bovine serum (FBS)); MDAMB231-LM2^16^ (lung-tropic variant of parental MDAMB231; Dr Joan Massague; DMEM/10% FBS); BT549 (human papillary TNBC; Dr Patricia Steeg; RPMI1640/10% FBS/1% penicillin–streptomycin/0.023 U ml^−1^ insulin); SUM159M1a^47^ (human anaplastic TNBC; Dr Yibin Kang; Ham’s F-12/10% FBS); SUM149PT^48^ (human BRCA1-mutant inflammatory TNBC; Dr Esta Sterneck; Ham’s F-12/10%FBS, 5 μg ml^−1^ insulin, 1 μg ml^−1^ hydrocortisone); MCF10Ca1h^49^ (tumorigenic Ras-transformed human mammary epithelial cell; Dr Fred Miller, DMEM/F-12/5% horse serum); 4T1^17^ (mouse TNBC; Dr Fred Miller; DMEM, 10% FBS). Cells were cultured in a humidified incubator at 37 °C with 5% CO_2_. All human cell lines that were not obtained directly from the American Type Culture Collection underwent authentication via short tandem repeat profiling (TransnetYX) between 2015 and 2023, and were used from the same freeze-down as the authenticated sample. The 4T1 mouse line was obtained directly from its originator and used at low passage after initial expansion for freeze-down. Mycoplasma testing of all cell lines was performed regularly using the Lookout Mycoplasma PCR Detection Kit (Sigma-Aldrich).

### Pharmacologic inhibitors

The pharmacologic inhibitor source, dose range and vehicle used for in vitro studies were as follows: TRULI (Selleckchem, E1061, 1–10 µM, dimethyl sulfoxide (DMSO)); defactinib (Selleckchem, S7654, 0.1–10 µM, DMSO); cytochalasin D (Selleckchem, S8184, 0.05–1 µM, DMSO); BMS986020 (Selleckchem, S3572, 1–10 µM, DMSO); Y27632 (Selleckchem, 1–10 µM, DMSO); H1152 (Selleckchem, 0.5–5 µM, S6214, DMSO); torkinib (Selleckchem, S2218, 50–500 nM, DMSO); MK3903 (Selleckchem, S8803, 0.5–10 µM, DMSO); GNE7883 (MedChem Express, HY-147214, 0.5–5 µM, DMSO); paclitaxel (Selleckchem, S1150, 1–25 nM, DMSO).

### Reporter constructs and lentiviral transduction

All cell lines were transduced with a constitutively expressed nuclear GFP marker (EF1alpha>nucGFP lentivirus, cat. no. 4624 Essen Bioscience) and selected by FACS. The SORE6>dsmCherry construct (18065-M02-412) and minCMV>dsmCherry control (18065-M04-412) pro-lentiviral constructs were modifications of our previously described constructs^14,15^, using destabilized mCherry in place of dsCopGFP (Extended Data Fig. 1a). Both have been deposited with Addgene (IDs 225665 and 225667). Titred lentivirus was generated by Applied StemCell. Exponentially growing cells already expressing the constitutive nucGFP were transduced by exposure to virus at a multiplicity of infection of 1 for 24 h without polybrene, then expanded and FACS-sorted for CD19 expression to ensure reporter integration. The low multiplicity of infection is critical to restrict reporter construct integration to one copy per cell. Cells were used within ten passages of reporter transduction. shYAP1 (Addgene #27368) and non-targeting control shRNA (Addgene #8453) lentiviral vectors were obtained from Addgene.

### Animal models and metastasis assays

Virgin female NCI Ath/Nu++ immunodeficient nude mice and immunocompetent BALB/c mice (NCI Charles River Labs) were used at 6–8 weeks of age. Experiments involving imaging markers were performed in the immunodeficient mice to avoid rejection of the fluorescent protein neoantigens. All mice were maintained in individually ventilated polycarbonate cates under specific pathogen-free conditions in a temperature-controlled room (72 ± 4 °F), humidity 30–70%, with a 12:12-h light–dark cycle. Animals had ad libitum access to Inotiv NIH 31 rodent chow and chlorinated water. For MDAMB231-LM2 orthotopic studies, 5 × 10^5^ labelled cells were implanted in the #2 and #7 mammary fat pads. For the pseudo-orthotopic format, 2.5 × 10^5^ unlabelled cells were injected into the #2 mammary fat pad to establish a pre-metastatic niche; after 10–15 days, 4 × 10^5^ labelled cells were injected via tail vein, and lungs were collected at various timepoints. For shYAP/shCON studies, lungs were harvested 28 days after tail vein injection and metastatic foci were counted on histologic sections immunostained for GFP. For 4T1 imaging studies, 4 × 10^4^ labelled cells were surgically implanted in #2 and #7 mammary fat pads of nude mice; primary tumours were resected at day 14 and lungs collected at days 9, 15 and 22 after removal of primary tumour. For immunocompetent ROCK inhibitor/paclitaxel studies, 10^4^ unmodified 4T1 cells were injected via tail vein into BALB/c mice, and lungs collected after 21 days for metastasis quantitation on sections stained with haematoxylin and eosin. The maximal tumour size permitted by the NCI ACUC (20 mm in any dimension) was never exceeded.

### In vivo drug treatment studies

The TEAD inhibitor GNE7883 (Chemieteck) was suspended in sunflower oil (Spectrum Chemical Corp) and dosed at 250 mg kg^−1^ by daily subcutaneous injection on the nape of the neck (4 days on, 2 days off) per Hagenbeek et al.^34^. The ROCK inhibitor Y27632 (SelleckChem, S1049) was prepared in saline and dosed at 10 mg kg^−1^ intraperitoneally twice weekly^50^. Paclitaxel (NCI Division of Veterinary Resources; 6 mg ml^−1^ stock in Cremaphor or castor oil) was diluted to 2.8 mg ml^−1^ in phosphate-buffered saline (PBS) and administered intravenously via tail vein injection at 10 mg kg^−1^ once weekly. The LPAR inhibitor BMS5986020 (SelleckChem S3572) was prepared in 0.5% methylcellulose in water and dosed at 5 mg kg^−1^ daily by oral gavage^51^. Drug schedules are shown in the schematics for each experiment.

### Circulating tumour cells

Mice orthotopically implanted with LM2 or 4T1 cells transduced with the nuclear GFP constitutive marker and the SORE6>dsmCherry CSC reporter were anaesthetized when tumours reached 0.5–1.0 cm diameter. One millilitre of fresh blood was collected via cardiac puncture into EDTA (1.6 mg ml^−1^) tubes and then pooled for four (LM2) or nine (4T1) mice. Samples were processed using Ficoll-Paque PLUS (Cytiva) per manufacturer’s instructions. Cells were cytospun onto slides that were scanned using an EVOS FL Auto 2 (Thermo Fisher Scientific) to quantify GFP^+^ cancer cells and mCherry^+^ CSCs. Higher-resolution images were acquired on a Nikon SoRa spinning disk confocal microscope.

### Tissue preparation for imaging and immunostaining

Primary tumours (~5 mm diameter) were excised after euthanasia, placed in ice-cold PBS, sliced into 1–2-mm slices and mounted onto glass-bottom dishes for confocal imaging. Lungs were inflated with ice-cold PBS and excised, and one lobe was laid flat onto a glass-bottom Ibidi dish for immediate confocal imaging. Remaining lobes were formalin-fixed for histology, or embedded in OCT (Tissue-Tek) and frozen for immunofluorescent staining.

### Confocal imaging and analysis of tissues

Primary tumours were imaged on a Nikon SoRa spinning disk confocal microscope (Photometrics BSI sCMOS camera, 20× plan-apochromat numerical aperture 0.75 objective lens) in tile mode with stitching in NIS-Elements software (v.5.4.2) and processed in Imaris (Oxford Instruments). A randomly selected 500 × 500 µm field was analysed per tumour. Lung metastases were imaged on a Zeiss LSM 880 (GaAsP spectral detector, plan-apochromat 40×/1.3 oil DIC UV-IR M27 objective lens). Then, 25–50 random 40× magnification fields per lung were captured, and data were normalized to fields scanned. ImageJ Fiji software was used to quantify metastatic lesion size and CSC numbers, based on GFP (all tumour cells) and mCherry (CSCs) fluorescence, with thresholds set using minCMV>dsmCherry control tissue.

### Flow cytometry

Subconfluent cultures were collected by trypsinization and analysed on a BD LSRFortessa SORP1 (Becton Dickinson) with FlowJo Software (Tree Star). minCMV>dsmCherry cells served as the gating controls for the SORE6>dsmCherry reporter. LPAR1 antibody was from Alomone Labs LTR (cat. no. ALR-031; 1:50) with secondary antibody anti-rabbit Alexa 670 (Invitrogen, cat. no. A31573; 1:500).

### FACS

Metastasis-bearing mouse lungs (*n* = 6) were excised, rinsed in ice-cold PBS, minced on ice for 5–7 min and passed twice through a 70-μm cell strainer (Falcon) to obtain single-cell suspensions. After centrifugation (300*g*, 5 min, 4 °C), resuspended cell pellets were filtered through 40-μm strainers, washed and sorted on a BD FACSAria Fusion, after staining with APC anti-human CD19 (BioLegend, cat. no. 392504; 5 µl per million cells). Tumour cells were gated based on GFP and/or CD19 positivity. After excluding doublets, dead cells and CD19-negative cells, the top 5% SORE6>dsmCherry-positive and bottom 20% SORE6>dsmCherry-negative cells were collected.

### Incucyte live cell imaging of CSC and nonCSC dynamics

An Incucyte S3 Live-Cell Imaging system (Essen Biosciences; 440/480 and 565/620 filters) was used to monitor CSC and nonCSC dynamics in 24-well plates (Corning, cat. no. 3524) seeded with 25,000 or 50,000 cells per well. Images were captured in the red and green channels every 3–4 h and analysed using the basic analyser in the Incucyte software. All tumour cells were identified by GFP^+^ nuclei. mCherry^+^ cells (above a threshold set using minCMV>dsmCherry controls) were classified as CSCs while mCherry^−^ cells were classified as nonCSCs. In many experiments, analysis definitions were validated by flow cytometry data confirming the correct percentage of CSCs at the endpoint. For ECM studies, dishes were precoated for 20 min at room temperature with fibronectin (10 µg ml^−1^, Gibco, cat. no. F1141-1MG) or collagen (150 µg ml^−1^; Gibco, cat. no. A10483-01) diluted in PBS, 0.12% v/v glacial acetic acid, before washing for immediate use.

### Live cell fate mapping and origin mapping

LM2 cultures were imaged in the Incucyte S3 system every 30 min starting 6 h after plating. SORE6+ CSCs were masked in red at each timepoint. Movies were generated to allow manual forward (fate) or reverse (origin) tracking of individual cells. SORE6+ CSCs were scored as having differentiated if they maintained loss of red signal for ≥3 h of tracking (six consecutive frames). In total, 20–40 SORE6+ cells per well from 3–4 wells per condition (60–110 cells per treatment group) were mapped, while twice as many SORE6− nonCSCs were mapped to capture the rarer dedifferentiation events.

### Matrix stiffness modulation

SoftSlip hydrogel-coated six-well plates (Matrigen) at 1 kPa (cat. no. SS6-EC1-EA) or 12 kPa (cat. no. SS6-EC12-EA) represented soft and stiff ECM environments respectively; standard tissue culture polystyrene (~3 GPa) served as a rigid control. Cells were seeded at 10^5^ cells per well, detached with Accutase (Millipore, cat. no. SCR005) after 4 days, and analysed by flow cytometry.

### Immunofluorescent staining

LM2 cells in Ibidi eight-well chamber glass-bottom slides were fixed in 4% paraformaldehyde, permeabilized with 0.5% Triton X-100 and blocked with 10% bovine serum albumin/PBS. Primary antibodies included YAP (#14074, Cell Signaling Technology; 1:200), GFP (ab6673, Abcam; 1:1,000) and mCherry (M11217, Thermofisher; 1:500). Secondary antibodies (all 1:500, from Thermo Fisher Scientific) included anti-goat-IgG Alexa 488 (#A11055), anti-rat-IgG Alexa 568 (#A11077) and anti-rabbit-IgG Alexa 647 (#A31573).

For Ki67 in metastases, 5-µm frozen sections were processed as above, incubated overnight with anti-Ki67 (Abcam ab16667, 1:1,000), followed by anti-rabbit-IgG Alexa 647 (ThermoFisher #A31573), counterstained with 4,6-diamidino-2-phenylindole (DAPI) and mounted with ProLong Gold Antifade Mountant (Thermo Fisher Scientific). mCherry (CSCs) and GFP (all tumour cells) were detected by endogenous fluorescence. Images were acquired on a Zeiss LSM 880 (40×/1.3 oil DIC UV-IR M27 objective, numerical aperture 1.3), with standardized settings across all samples.

Multiplex YAP/tumour cell staining on formalin-fixed, paraffin-embedded lung sections used a Leica Biosystems Bond RX autostainer (Bond Polymer Refine Kit, citrate antigen retrieval). Sequential rounds of primary antibody incubation used anti-YAP (Cell Signaling Technology #14074,; 1:100, 30 min) or anti-mCherry (Thermofisher #M11217, 1:500, overnight) in the first round; anti-GFP antibody (Abcam #ab183734; 1:1,200, 30 min) in the second round; and anti-Ki67 (Cell Signaling Technologies #9027; 1:200) in the third round. OPAL fluorophore detection (OPAL FL 570 (YAP), FL 690 (mCherry), FL 520 (GFP), FL 620 (Ki67), Akoya Bioscience) and epitope stripping (Bond Epitope Retrieval 24) were followed by DAPI counterstaining. Slides were scanned on an Aperio Scanscope FL and quantified in Halo software.

### Image analysis

#### HALO HighPlex

Ki67^+^ cells in large metastases were quantitated with the HALO (Indica Labs) HighPlex FL version 4.2.14 module. Metastatic lesions were manually delineated as regions of interest (ROIs) based on GFP fluorescence, and cells were segmented into nuclear and cytoplasmic subpopulations for marker quantification.

#### Image J

CSC and nonCSC quantitation during early lung colonization was performed using ImageJ to measure fluorescence intensity and cell counts, with GFP identifying all tumour cells and mCherry identifying CSCs (threshold set using minCMV>dsmCherry controls).

#### YAP quantitation

For YAP-stained tissues and cells, tumour cells were segmented and quantified for YAP expression using a custom script written in Python (v. 3.10). Nuclear and cellular segmentation used the Cellpose deep learning algorithm^52^. DAPI intensity was used to segment nuclei using a custom model that was trained with the NIH HPC Biowulf cluster (http://hpc.nih.gov); cellular segmentation with DAPI and GFP used the pretrained cyto3 model. Cytoplasmic ROIs were derived by subtracting nuclear from cellular ROIs. YAP intensity was quantified in each compartment, along with cellular SORE6>dsmCherry intensity.

### Western blot analysis

Cell lysates in RIPA buffer with protease and phosphatase inhibitors (Thermo Fisher Scientific) were run at 15 µg protein per well on 4–20% Mini-PROTEAN TGX gels (Bio-Rad) under reducing conditions and transferred to polyvinylidene fluoride membranes. Primary antibodies used were (Cell Signaling Technologies): phospho-YAP(S127), #13008S; YAP, #14074S; phospho-TAZ(S89), #59971S; TAZ #83669S; β-actin, #8H10D10; phospho-FAK, (Y397) #3283S; FAK, #3285S. GAPDH antibody was from AbCam (#Ab9484). Signal was detected with SuperSignal West Femto Substrate (Thermo Scientific #34096) and imaged on a Bio-Rad ChemiDoc Imaging System.

### siRNA-mediated knockdown

SMARTpool ON-TARGETplus siRNAs (Horizon Discovery) targeting YAP (L-012200), TAZ (L-016083), KLF5 (L-013571), ID1 (L-005051) and SNAI1 (LZ-010847-01-0005) were transfected into LM2 cells using Lipofectamine RNAiMAX (Thermo Fisher Scientific, #13778100) per the manufacturer’s instructions. Cells were collected 72 h after transfection for confirmation of knockdown and downstream analyses.

### Reverse-transcription qPCR

RNA was isolated with the RNeasy Mini Kit (QIAGEN) and reverse transcribed with SuperScript III RT kit (Thermo Fisher Scientific) per the manufacturer’s instructions. Quantitative polymerase chain reaction (qPCR) used the SoFast qPCR Mastermix (Bio-Rad) on a Bio-Rad CFX Opus96, with all reactions in triplicate, and gene expression calculated using the 2^(−ΔΔCt)^ method normalized to PPIA. Primers were purchased from IDT. ID1 forward: 5′-CCAGAACCGCAAGGTGAG-3′, ID1 reverse: 5′-GGTCCCTGATGTAGTCGATGA-3′; KLF5 forward: 5′-GGAGAAACGACGCATCCACTAC-3′, KLF5 reverse: 5′-GAACCTCCAGTCGCAGCCTTC-3′; CTGF forward: 5′-CTTGCGAAGCTGACCTGGAAGA-3′, CTGF reverse: 5′-CCGTCGGTACATACTCCACAGA-3′; SNAI1 forward: 5′-TGCCCTCAAGATGCACATCCGA-3′, SNAl1 reverse: 5′-GGGACAGGAGAAGGGCTTCTC-3′. PP1A forward: 5′-GTCAACCCCACCGTGTTCTT-3′, PPIA reverse: 5′-CTGCTGTCTTTGGGACCTTGT-3′.

### RNA sequencing and analysis

RNA was prepared from FACS-sorted LM2 CSCs and nonCSCs collected directly into TRIzol reagent (Ambion) and quality-assessed on an Agilent 2100 Bioanalyzer (all RNA integrity numbers (RINs) >9). Libraries were generated with Illumina TruSeq Stranded Total RNA Kit RS-122-2201 and sequenced on Illumina HiSeq with 3000/4000 sequencing chemistry. Reads were trimmed with Cutadapt and aligned to human reference genome hg19. Differential gene expression analysis between CSCs and nonCSCs was performed using DESeq2. Upstream regulator analysis on differentially expressed genes (false discovery rate ≤0.25) used Ingenuity Pathway Analysis (Qiagen build 9.0, version B9584291). Gene set enrichment analysis was performed on the full DESeq2-ranked gene list, with a fetal mammary stem cell signature^53^ added to the curated gene sets (C2).

### ATAC-seq and analysis

Two independent preparations of FACS-sorted LM2 SORE6+ CSCs and SORE6− nonCSCs (recovered in culture overnight; four samples total) were processed using the Active Motif ATAC-Seq Kit (#53150). A total of 100,000 cells were lysed, tagmented at 37 °C for 30 min, purified, PCR-amplified with indexed primers and sequenced on a NovaSeq 6000 SP. All sequence analyses were carried out on NIH Biowulf2 high-performance computing environment using software default parameters, unless specified otherwise. The fastq sequence reads were aligned to the reference genome hg38 using Bowtie2 (2-2.5.3); peaks called with MACS2 (2.2.7.1, --broad --nomodel). Analysis was performed with bamCoverage, computeMatrix and plotHeatmap from deepTools (3.5.5). Transcription factor motif analysis was performed with Homer (5.1; findMotifsGenome.pl and scanMotifGenomeWide.pl). H3K27Ac chromatin immunoprecipitation sequencing (ChIP-seq) data for MDAMB231 cell line were downloaded from the SRA database (SRR949140) and aligned to hg38; peaks were called with MACS2 to define enhancer regions.

### Statistics and reproducibility

No statistical methods were used to predetermine sample sizes, but our sample sizes are similar to those reported in similar studies^15,54^. Age-matched mice were randomized by cage between treatment groups; animal staff were blinded to treatment allocation for dosing. Investigators were blinded to sample identity in analysis of images for CSC quantitation and movies for single cell fate-mapping studies by assigning a code number to each image and unblinding to sample identity at the end of the analysis. For fate mapping, the same samples were analysed by two independent investigators. All enrolled mice were included in the final analysis except: for Fig. 7b–d, two mice developed severe dermatitis that precluded imaging; and for Extended Data Fig. 7i–l, three mice were lost to acute paclitaxel toxicity. See the Source Data for group assignments. Occasional Incucyte timepoints affected by mechanical disturbance of the incubator were excluded across all treatment groups and are flagged in the Source Data. Statistical analyses used GraphPad Prism (v10.1.1), except for contingency tables (https://vassarstats.net/). Non-parametric analyses were used for in vivo data. In vitro data were assumed to be normally distributed, but this was not formally tested. Multiple comparison corrections were applied where appropriate. The significance threshold was *P* < 0.05, and exact *P* values and specific tests are indicated in the figure legends or Source Data. Unless otherwise indicated, results are representative of two or more independent replicate experiments, with original data provided in the Source Data. Due to their expensive and/or labour-intensive nature, individual in vivo experiments and fate or origin mapping experiments were performed only once for a given combination of cell line and intervention.

### Reporting summary

Further information on research design is available in the Nature Portfolio Reporting Summary linked to this article.

## Online content

Any methods, additional references, Nature Portfolio reporting summaries, source data, extended data, supplementary information, acknowledgements, peer review information; details of author contributions and competing interests; and statements of data and code availability are available at 10.1038/s41556-026-02013-8.

## Supplementary information

Reporting Summary

Peer Review File

Supplementary Table 1 Effect of pharmacologic modulation of upstream inputs to YAP/TAZ node on CSC dynamics Pharmacologic agents targeting potential upstream inputs to the YAP/TAZ node were tested at multiple concentrations across three breast cancer models for selective effects on the CSC population, using Incucyte live cell imaging in unsorted cell cultures. Scores reflect the relative effect size for the agent specifically on the CSCs, as assessed by plotting CSCs as a function of total cell number for drug concentrations that showed minimal toxicity and low cytostatic effect for the culture as a whole. Maximum effect size of the intervention was scored as: little or no effect (<25% change), moderate effect (25–50% change) or strong effect (>50% change). ROCK lies downstream of both bioactive lipid signalling and integrin activation by ECM attachment. Source datasets for the summarized results in this table are given in the subsequent tabs in the workbook.

## Source data

Source Data Fig. 1 Statistical source data for Fig. 1.

Source Data Fig. 2 Statistical source data for Fig. 2.

Source Data Fig. 3 Statistical source data for Fig. 3.

Source Data Fig. 4 Statistical source data for Fig. 4.

Source Data Fig. 5 Statistical source data for Fig. 5.

Source Data Fig. 6 Statistical source data for Fig. 6.

Source Data Fig. 7 Statistical source data for Fig. 7.

Source Data Extended Data Fig. 1 Statistical source data for Extended Data Fig. 1.

Source Data Extended Data Fig. 2 Statistical source data for Extended Data Fig. 2.

Source Data Extended Data Fig. 3 Statistical source data for Extended Data Fig. 3.

Source Data Extended Data Fig. 4 Statistical source data for Extended Data Fig. 4.

Source Data Extended Data Fig. 5 Statistical source data for Extended Data Fig. 5.

Source Data Extended Data Fig. 6 Statistical source data for Extended Data Fig. 6.

Source Data Extended Data Fig. 7 Statistical source data for Extended Data Fig. 7.

Source Data Fig. 5, and Extended Data Figs. 3 and 4 Unprocessed western blots for Fig. 5e,f,j,m,n, and Extended Data Figs. 3c and 4e.

## Data Availability

RNA sequencing and ATAC-seq data that support the findings in this study have been deposited in the Gene Expression Omnibus (GEO) database under accession code GSE292567 (superset, consisting of GSE292485, GSE292476 and GSE292539). The previously published H3K27Ac ChIP-seq dataset for MDAMB231 that was reanalysed for this study is available via GEO under accession code GSM1204474, with raw data available in the SRA database (https://www.ncbi.nlm.nih.gov/sra/?term=SRR949140). Source data are provided with this paper. All other data supporting the findings of this study are available from the corresponding author upon reasonable request.

## Code availability

Custom code generated for analysis of YAP immunostained images has been uploaded to GitHub (https://github.com/CCRMicroscopyCore/tangb) and is available via Zenodo at 10.5281/zenodo.15675993 (ref. ^55^).
