# Supplementary material for: Cancer stem cells are hyper-responsive sensors of the tumour microenvironment and regulate metastasis dynamics through YAP/TAZ
Source: Nat Cell Biol. 2026 Jul 27;28(8):1728–41. doi: 10.1038/s41556-026-02013-8 (PMC13457095; doi:10.1038/s41556-026-02013-8)

Tang et al. Fig. 5

Figure 5e

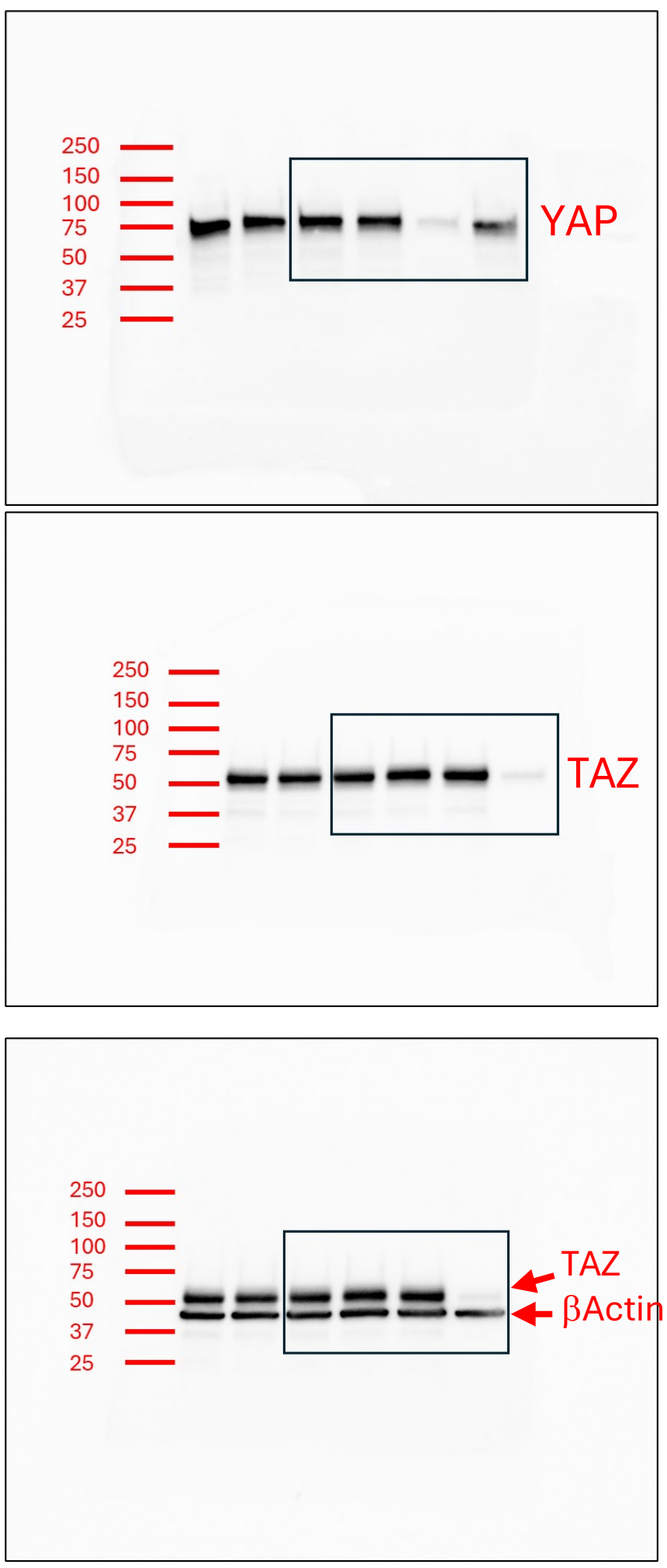

Figure 5f

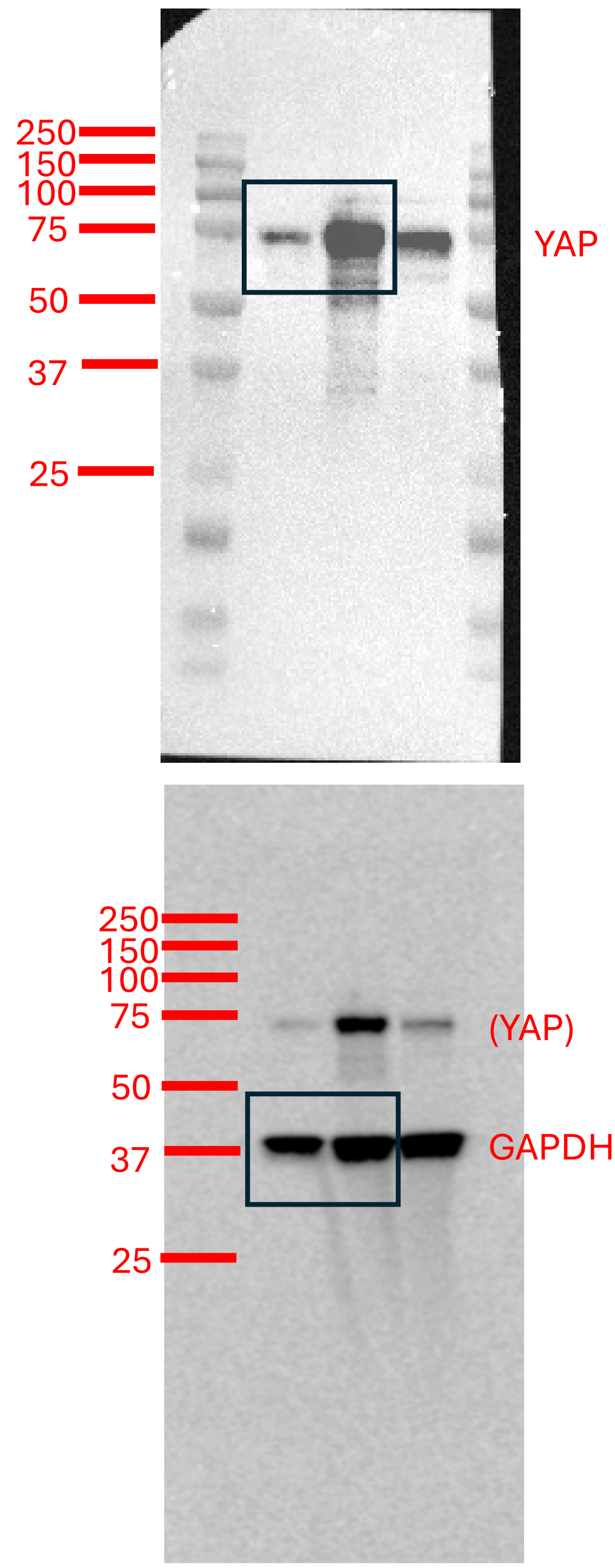

Tang et al. Fig. 5 cont.

Figure 5j

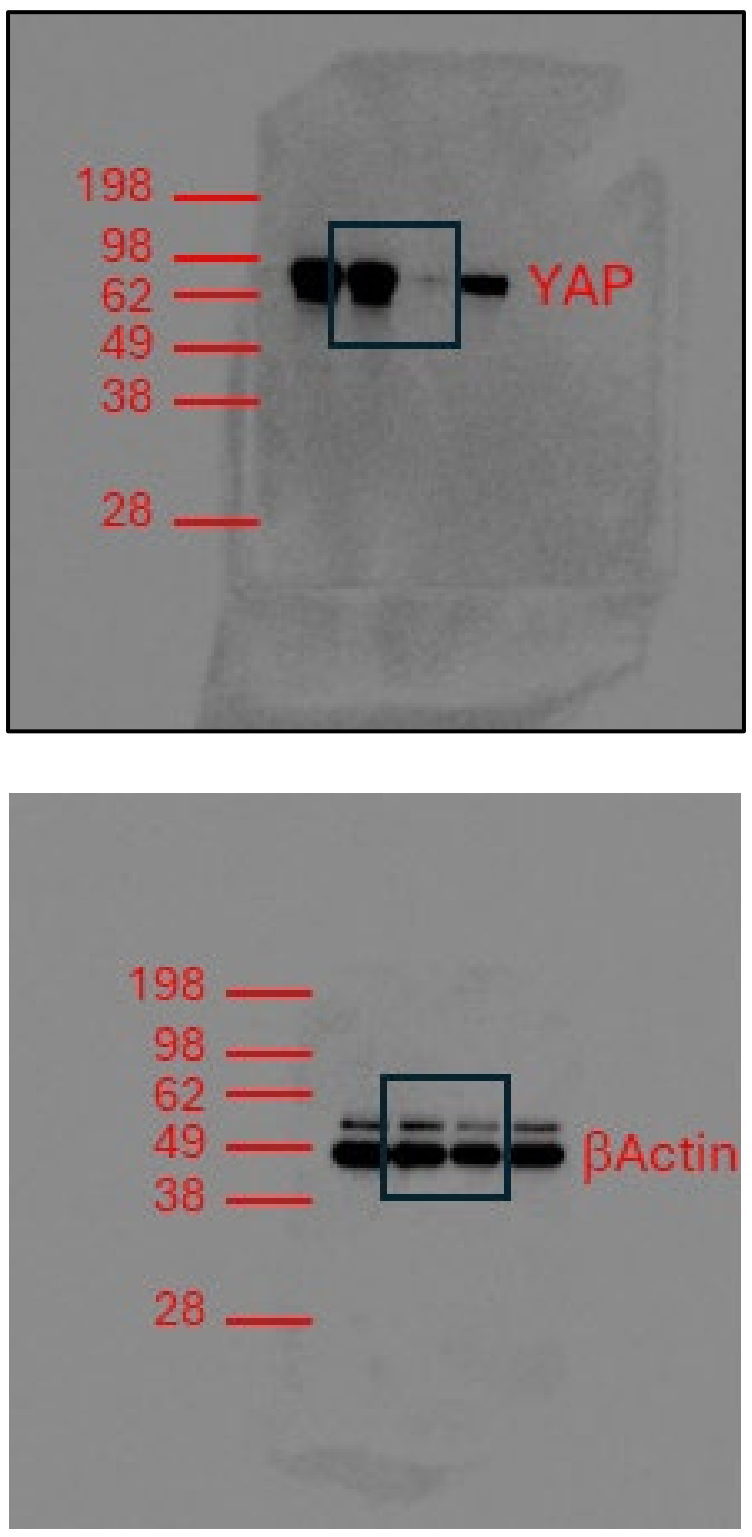

Figure 5m

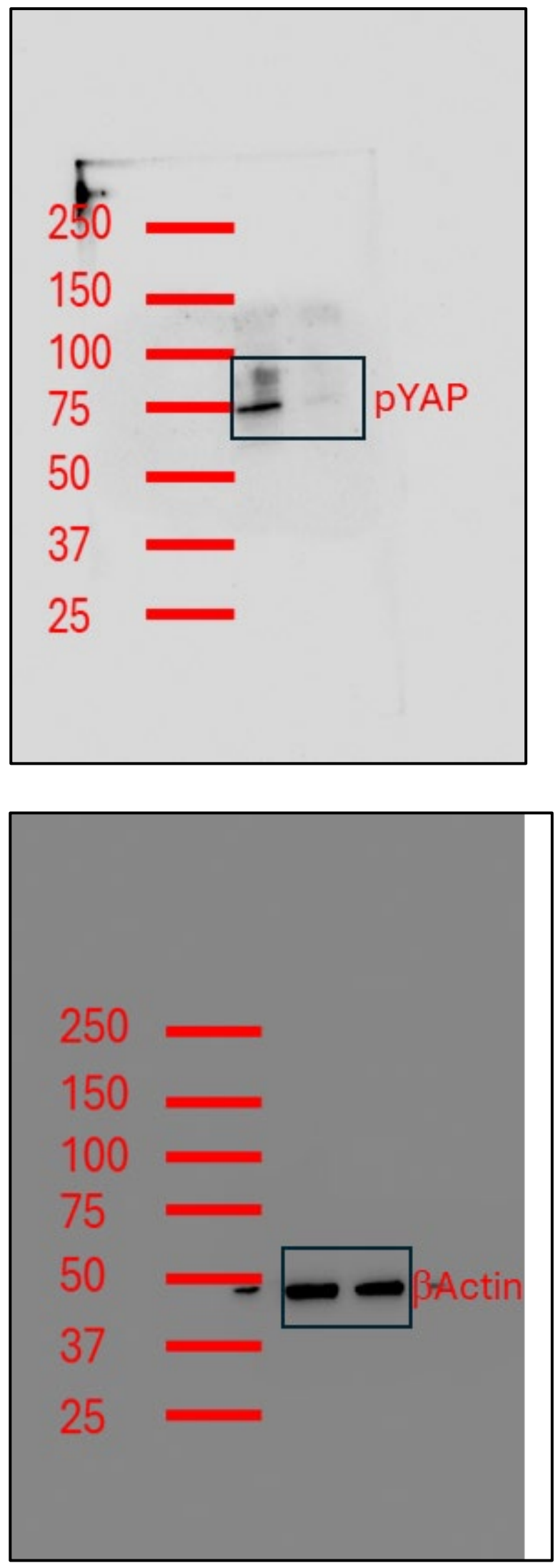

Figure 5n

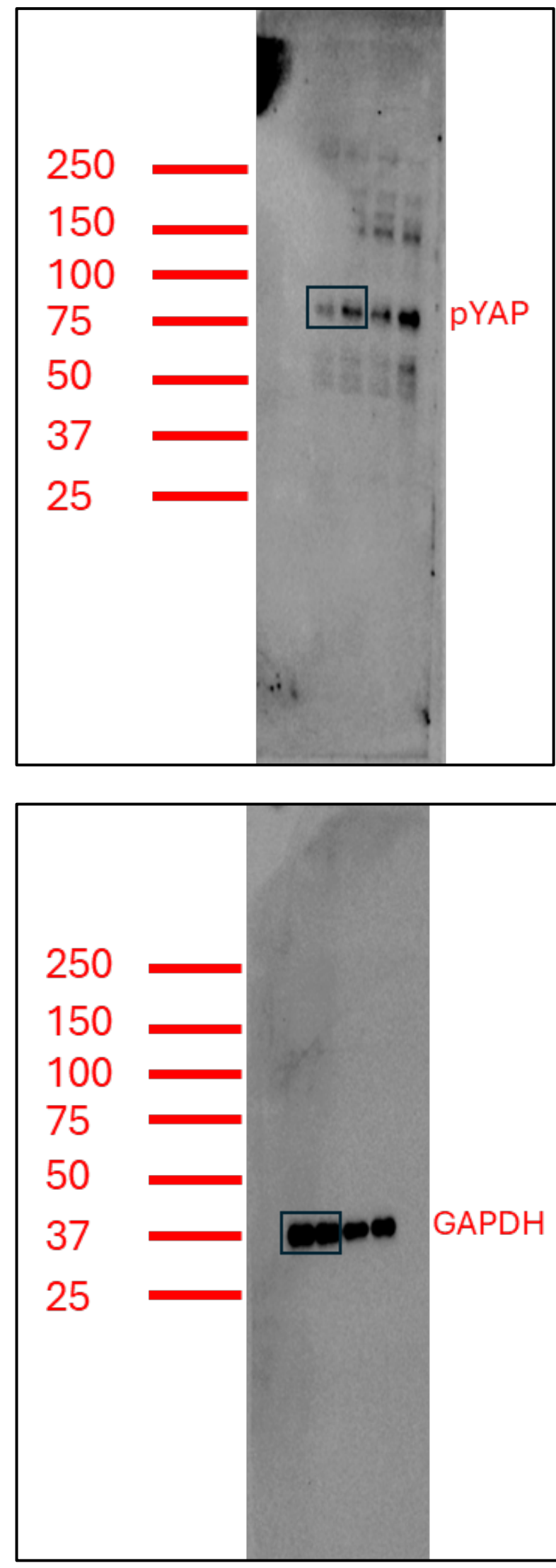

Tang et al. Extended Data Fig. 3

Extended Data Fig. 3c

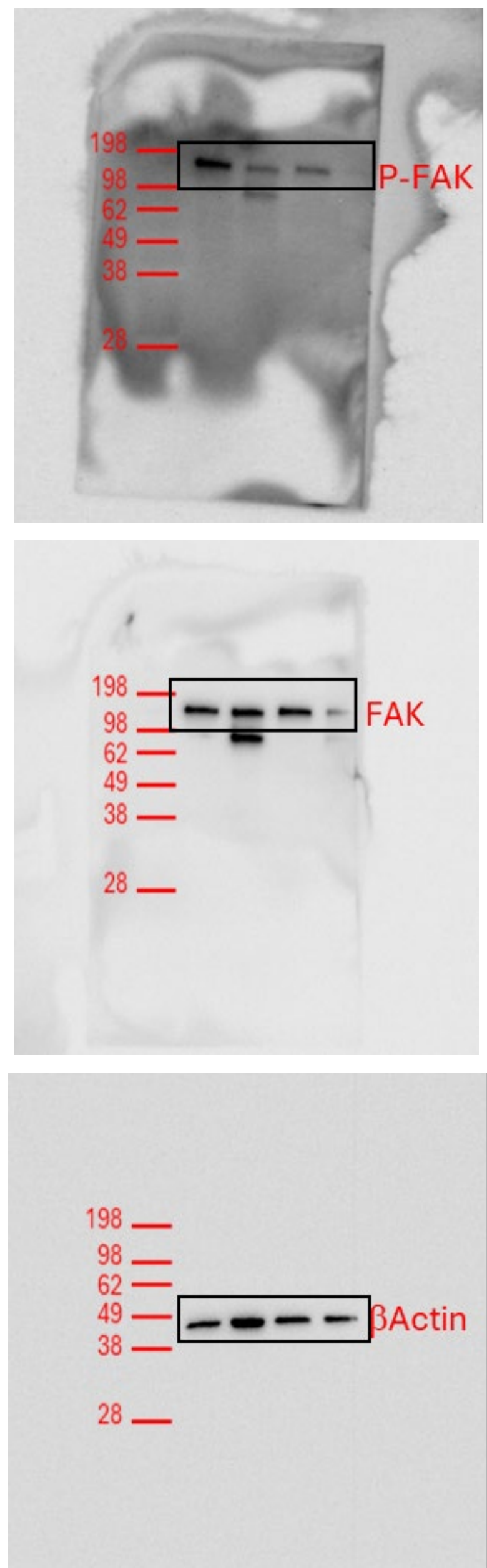

Tang et al. Extended Data Fig. 4

Extended Data Fig. 4e

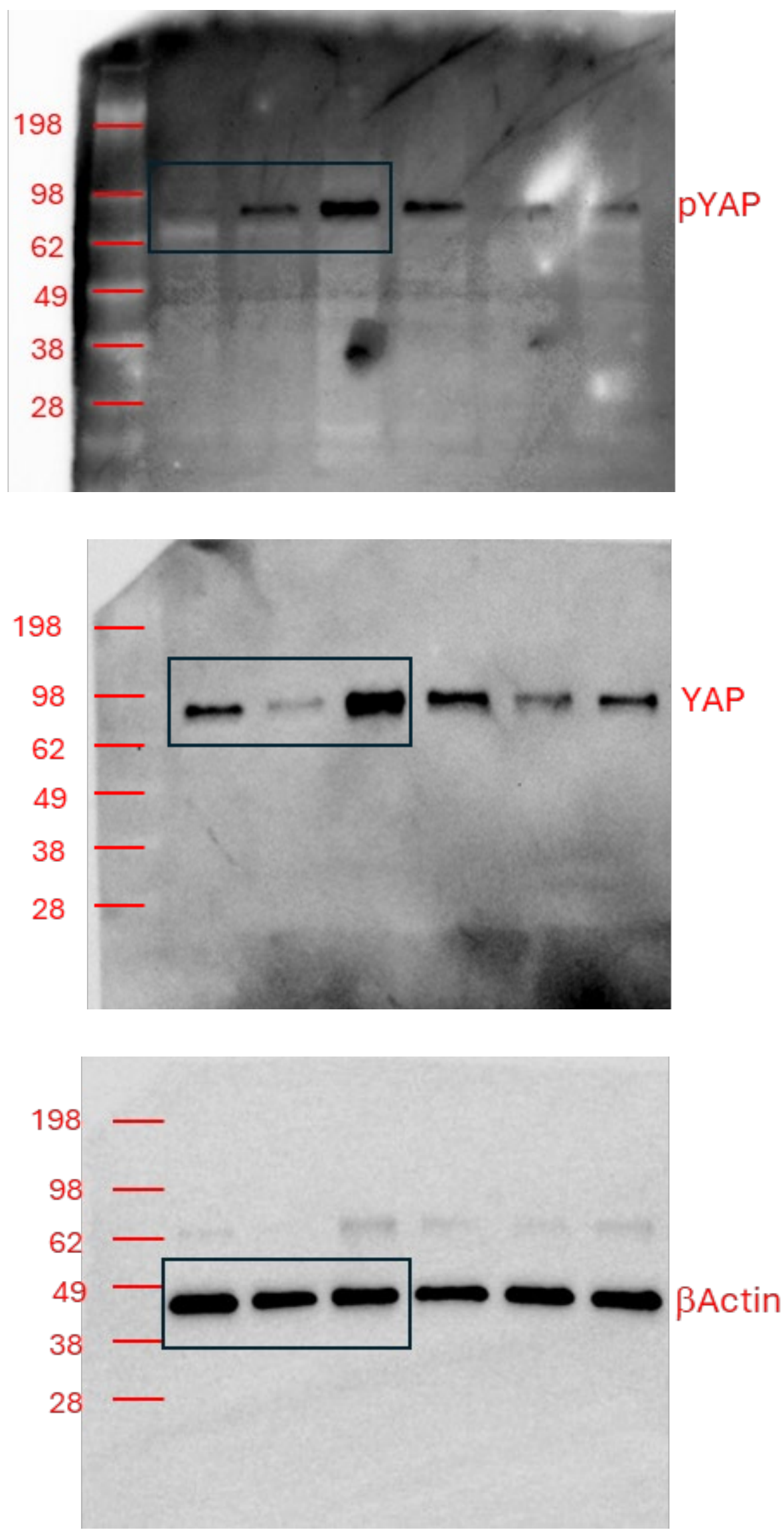

Supplement: Supplementary file 1 [file 41556_2026_2013_MOESM18_ESM.pdf]
