# Supplementary material for: Cancer stem cells are hyper-responsive sensors of the tumour microenvironment and regulate metastasis dynamics through YAP/TAZ
Source: Nat Cell Biol. 2026 Jul 27;28(8):1728–41. doi: 10.1038/s41556-026-02013-8 (PMC13457095; doi:10.1038/s41556-026-02013-8)
Supplement: Supplementary file 2 [file 41556_2026_2013_MOESM1_ESM.pdf]

nature portfolio

Corresponding author(s): Lalage Wakefield

Last updated by author(s): Jun 5, 2026

Reporting Summary

Nature Portfolio wishes to improve the reproducibility of the work that we publish. This form provides structure for consistency and transparency in reporting. For further information on Nature Portfolio policies, see our Editorial Policies and the Editorial Policy Checklist.

Statistics

For all statistical analyses, confirm that the following items are present in the figure legend, table legend, main text, or Methods section.

n/a	Confirmed	
☐	☒	The exact sample size (*n*) for each experimental group/condition, given as a discrete number and unit of measurement
☐	☒	A statement on whether measurements were taken from distinct samples or whether the same sample was measured repeatedly
☐	☒	The statistical test(s) used AND whether they are one- or two-sided<br>*Only common tests should be described solely by name; describe more complex techniques in the Methods section.*
☒	☐	A description of all covariates tested
☐	☒	A description of any assumptions or corrections, such as tests of normality and adjustment for multiple comparisons
☐	☒	A full description of the statistical parameters including central tendency (e.g. means) or other basic estimates (e.g. regression coefficient) AND variation (e.g. standard deviation) or associated estimates of uncertainty (e.g. confidence intervals)
☐	☒	For null hypothesis testing, the test statistic (e.g. *F*, *t*, *r*) with confidence intervals, effect sizes, degrees of freedom and *P* value noted<br>*Give P values as exact values whenever suitable.*
☒	☐	For Bayesian analysis, information on the choice of priors and Markov chain Monte Carlo settings
☒	☐	For hierarchical and complex designs, identification of the appropriate level for tests and full reporting of outcomes
☒	☐	Estimates of effect sizes (e.g. Cohen's *d*, Pearson's *r*), indicating how they were calculated

Our web collection on statistics for biologists contains articles on many of the points above.

Software and code

Policy information about availability of computer code

Data collection	NLS Elements v5.41-v5.42; Zeiss Zen Black v.2.3; Incucyte v2019A-v2025C
Data analysis	FloJo (v10.5-10.10); Imaris (v9.9.0); Halo high-plex FL (v4.2.14); ImageJ Fiji-window-x64. Qiagen Ingenuity Pathway Analysis (build 9.0, version B9584291), Bowtie2 (v2-2.5.3), MACS2 (v 2.2.7.1), DeepTools (v 3.5.5), Homer (v 5.1). Custom Python scripts (v3.10) were used for image analysis (https://doi.org/10.5281/zenodo.15675993). Segmentation used the Cellpose deep-learning algorithm (Stringer, C., Pachitariu, M. Nat Methods 19, 1634–1641, 2022; https://doi.org/10.1038/s41592-022-01663-4). A custom Cellpose model was trained using the NIH HPC Biowulf cluster (http://hpc.nih.gov) and based on a pre-trained cyto3 model.

For manuscripts utilizing custom algorithms or software that are central to the research but not yet described in published literature, software must be made available to editors and reviewers. We strongly encourage code deposition in a community repository (e.g. GitHub). See the Nature Portfolio guidelines for submitting code & software for further information.

Data

Policy information about availability of data

All manuscripts must include a data availability statement. This statement should provide the following information, where applicable:

- Accession codes, unique identifiers, or web links for publicly available datasets
- A description of any restrictions on data availability
- For clinical datasets or third party data, please ensure that the statement adheres to our policy

Transcriptomic and ATAC-Seq data are available from the Gene Expression Omnibus under accession number GSE292567 for the superset. Numerical datasets supporting the current study are available in the source data files or from the corresponding author on reasonable request.

Research involving human participants, their data, or biological material

Policy information about studies with human participants or human data. See also policy information about sex, gender (identity/presentation), and sexual orientation and race, ethnicity and racism.

Reporting on sex and gender	n/a
Reporting on race, ethnicity, or other socially relevant groupings	n/a
Population characteristics	n/a
Recruitment	n/a
Ethics oversight	n/a

Note that full information on the approval of the study protocol must also be provided in the manuscript.

Field-specific reporting

Please select the one below that is the best fit for your research. If you are not sure, read the appropriate sections before making your selection.

☒ Life sciences ☐ Behavioural & social sciences ☐ Ecological, evolutionary & environmental sciences

For a reference copy of the document with all sections, see nature.com/documents/nr-reporting-summary-flat.pdf

Life sciences study design

All studies must disclose on these points even when the disclosure is negative.

Sample size	Sample size was determined based on general standards for biological studies and requirements for statistical analysis, attempting to have a minimum of n=3 biological replicates for in vitro studies. n=3-10 biological replicates were used for in vivo studies depending on the study goal and analysis tools used, so as to strike a balance between high statistical significance and practical issues (eg. imaging throughput). Pharmacologic intervention studies in vivo typically used the larger numbers due to the high intrinsic variability of the metastasis burden endpoint.
Data exclusions	In a small number of instances an enrolled animal was dropped from a study if there was an endpoint-unrelated adverse event (acute paclitaxel toxicity or severe dermatitis precluding reliable imaging). Occasional Incucyte timepoints affected by mechanical disturbance of the incubator were excluded across all treatment groups. All cases of data exclusion are flagged in the Source data files.
Replication	Most in vitro results were replicated in at least 2 independent experiments, either in the same cell line and/or in additional cell lines to demonstrate robustness of the phenomenon as well as reproducibility. Fate mapping experiments were only performed once due to their labor-intensive nature, but the control condition replicated well between independent experiments. In vivo results were shown to be robust by repeating experiments using different model formats (eg tail vein vs orthotopic implantation), different breast cancer models (MDAMB231-LM2 vs 4T1), or different pharmacologic interventions on the same pathway (eg. LPAR inhibition and ROCK inhibition)
Randomization	Mice were randomized to study intervention groups by cage.
Blinding	Investigators were blinded to sample identity in analysis of images for CSC quantitation and movies for single cell fate-mapping studies by assigning a code number to each image and unblinding to sample identity at the end of the analysis. For fate-mapping, the same samples were analysed by two independent investigators.

Reporting for specific materials, systems and methods

We require information from authors about some types of materials, experimental systems and methods used in many studies. Here, indicate whether each material, system or method listed is relevant to your study. If you are not sure if a list item applies to your research, read the appropriate section before selecting a response.

Materials & experimental systems

n/a	Involved in the study
☐	☒ Antibodies
☐	☒ Eukaryotic cell lines
☐	☐ Palaeontology and archaeology
☐	☒ Animals and other organisms
☐	☐ Clinical data
☐	☐ Dual use research of concern
☐	☐ Plants

Methods

n/a	Involved in the study
☐	☐ ChIP-seq
☐	☒ Flow cytometry
☐	☐ MRI-based neuroimaging

Antibodies

Antibodies used

pYAP Ser127 (Cell Signaling Technology, #13008S, 1:200)
YAP (Cell Signaling Technology, #14074S, 1:200 for Westerns and IF on cells, 1:100 for multiplex OPAL IF)
TAZ (Cell Signaling Technology, #83669S, 1:200)
pTAZ (S89) (Cell Signaling Technology, #59971S, 1:200)
Ki67 (Abcam, #ab16667, 1:1000 for IF on frozen sections)
Ki67 (Cell Signaling Technology, #9027, 1:200 for multiplex OPAL IF)
GFP (Abcam, #ab6673, 1:1000)
GFP (Abcam, #ab183737, 1:500 and 1:1200 for multiplex OPAL IF)
mCherry antibody (16D7) (Thermofisher Scientific, #M11217, 1:500)
B-Actin (Cell Signaling Technology, #8H10D10, 1:3000)
GAPDH (Abcam, #ab9484, 1:3000)
FAK (Cell Signaling Technology, # 3285S, 1:200)
pFAK (Y397) (Cell Signaling Technology, #3283S, 1:200)
Anti-Goat-IgG Alexa 488, (Invitrogen, #A11055, 1:500)
Anti-Rabbit-IgG Alexa 647 (Invitrogen, #A31573, 1:500)
Anti-Rat-IgG Alexa 568 (Invitrogen, #A11077, 1:500)
CD19 (Biolegend, #392504, 5ul/million cells)
LPAR1 (Alomone Labs LTR, #ALR-031, 1:50)
Anti-Mouse-IgG HRP (Cell Signaling Technology, #7076, 1:3000)
Anti-Rabbit-IgG HRP (Cell Signaling Technology, #7074, 1:3000)

Validation

All antibodies in this study were commercially available, validated by the manufacturer and widely used in the literature. We also independently validated specificity of YAP and TAZ antibodies in siRNA knockdown experiments, and mCherry and GFP antibodies by using tissue or cell line controls that did not express these foreign proteins.

Eukaryotic cell lines

Policy information about cell lines and Sex and Gender in Research

Cell line source(s)

MDAMB231 (ATCC, HTB-26)
BT549 (ATCC, HTB-122)
MDAMB231 (ATCC, HTB-26)
MDAMB231-LM2 (Dr. Joan Massague, Memorial Sloan Kettering Cancer Center)
SUM159M1a (Dr. Yibin Kang, Princeton University)
SUM149PT (Dr. Esta Sterneck, Frederick National Laboratory for Cancer Research)
MCF10Ca1h (Dr. Fred Miller, Karmanos Cancer Institute)
4T1 (Dr. Fred Miller, Karmanos Cancer Institute)
All cell lines were from female subjects (human and mouse)

Authentication

All human cell lines underwent authentication via short tandem repeat (STR) profiling (TransnetYX Cat # Cell ID GenePrint 10

Mycoplasma contamination

Mycoplasma testing was performed regularly using the Lookout Mycoplasma PCR Detection Kit (Sigma-Aldrich, St.Louis MO).

Commonly misidentified lines
(See ICLAC register)

No commonly misidentified cell lines were used in the study. Cell lines used in the study are not registered in ICLAC.

Palaeontology and Archaeology

Specimen provenance

n/a

Specimen deposition | n/a

Dating methods | n/a

☐ Tick this box to confirm that the raw and calibrated dates are available in the paper or in Supplementary Information.

Ethics oversight | n/a

Note that full information on the approval of the study protocol must also be provided in the manuscript.

Animals and other research organisms

Policy information about studies involving animals; ARRIVE guidelines recommended for reporting animal research, and Sex and Gender in Research

Laboratory animals	Virgin female NCI Athymic Nu++ immunodeficient mice or immunocompetent BALB/c mice aged 6-8 weeks were used for this study
Wild animals	n/a
Reporting on sex	Only female mice were used
Field-collected samples	n/a
Ethics oversight	Animal studies were approved by the Institutional Animal Care and Use Committee of the Center for Cancer Research, National Cancer Institute, NIH

Note that full information on the approval of the study protocol must also be provided in the manuscript.

Clinical data

Policy information about clinical studies

All manuscripts should comply with the ICMJE guidelines for publication of clinical research and a completed CONSORT checklist must be included with all submissions.

Clinical trial registration	n/a
Study protocol	n/a
Data collection	n/a
Outcomes	n/a

Dual use research of concern

Policy information about dual use research of concern

Hazards

Could the accidental, deliberate or reckless misuse of agents or technologies generated in the work, or the application of information presented in the manuscript, pose a threat to:

No	Yes	
☒	☐	Public health
☒	☐	National security
☒	☐	Crops and/or livestock
☒	☐	Ecosystems
☒	☐	Any other significant area

Experiments of concern

Does the work involve any of these experiments of concern:

No	Yes	
☒	☐	Demonstrate how to render a vaccine ineffective
☒	☐	Confer resistance to therapeutically useful antibiotics or antiviral agents
☒	☐	Enhance the virulence of a pathogen or render a nonpathogen virulent
☒	☐	Increase transmissibility of a pathogen
☒	☐	Alter the host range of a pathogen
☒	☐	Enable evasion of diagnostic/detection modalities
☒	☐	Enable the weaponization of a biological agent or toxin
☒	☐	Any other potentially harmful combination of experiments and agents

Plants

Seed stocks	n/a
Novel plant genotypes	n/a
Authentication	n/a

ChIP-seq

Data deposition

☐ Confirm that both raw and final processed data have been deposited in a public database such as GEO.

☐ Confirm that you have deposited or provided access to graph files (e.g. BED files) for the called peaks.

Data access links<br>*May remain private before publication.*	*For "Initial submission" or "Revised version" documents, provide reviewer access links. For your "Final submission" document, provide a link to the deposited data.*
Files in database submission	*Provide a list of all files available in the database submission.*
Genome browser session<br>(e.g. UCSC)	*Provide a link to an anonymized genome browser session for "Initial submission" and "Revised version" documents only, to enable peer review. Write "no longer applicable" for "Final submission" documents.*

Methodology

Replicates	*Describe the experimental replicates, specifying number, type and replicate agreement.*
Sequencing depth	*Describe the sequencing depth for each experiment, providing the total number of reads, uniquely mapped reads, length of reads and whether they were paired- or single-end.*
Antibodies	*Describe the antibodies used for the ChIP-seq experiments; as applicable, provide supplier name, catalog number, clone name, and lot number.*
Peak calling parameters	*Specify the command line program and parameters used for read mapping and peak calling, including the ChIP, control and index files used.*
Data quality	*Describe the methods used to ensure data quality in full detail, including how many peaks are at FDR 5% and above 5-fold enrichment.*
Software	*Describe the software used to collect and analyze the ChIP-seq data. For custom code that has been deposited into a community repository, provide accession details.*

Flow Cytometry

Plots

Confirm that:

☒ The axis labels state the marker and fluorochrome used (e.g. CD4-FITC).

☒ The axis scales are clearly visible. Include numbers along axes only for bottom left plot of group (a 'group' is an analysis of identical markers).

☒ All plots are contour plots with outliers or pseudocolor plots.

☒ A numerical value for number of cells or percentage (with statistics) is provided.

Methodology

Sample preparation	In vitro cell cultures were harvested by trypsinization or cells were recovered from metastasis-bearing lungs by mechanical dissociation and graded filtration. Samples were then stained where applicable with labelled antibodies.
Instrument	Flow cytometry: BD LSRFortessa SORP I (Becton Dickinson); FACS sorting: BD FACSAria Fusion (Becton Dickinson)
Software	FLowJo software (Tree star) v10.5-v10.10
Cell population abundance	A minimum of 20,000 cells/ fraction were recovered from FACS sorts, and purity was assessed by flow cytometry on the sorted population
Gating strategy	FSC/SSC gates were set to isolate live single cells. For antibody staining, isotype control was used to set the gate. For the SORE6>dsmCherry reporter, a parallel sample with the control minCMV>dsmCherry reporter was used to set a gate such that no more than 0.2% of cells appeared in the positive gate in the control sample. This gate was then used for the samples containing the SORE6>dsmCherry reporter.

☒ Tick this box to confirm that a figure exemplifying the gating strategy is provided in the Supplementary Information.

Magnetic resonance imaging

Experimental design

Design type	n/a
Design specifications	n/a
Behavioral performance measures	n/a

Acquisition

Imaging type(s)	n/a
Field strength	n/a
Sequence & imaging parameters	n/a
Area of acquisition	n/a

Diffusion MRI ☐ Used ☐ Not used

Preprocessing

Preprocessing software	n/a
Normalization	n/a
Normalization template	n/a
Noise and artifact removal	n/a
Volume censoring	n/a

Statistical modeling & inference

Model type and settings: n/a

Effect(s) tested: n/a

Specify type of analysis: ☐ Whole brain ☐ ROI-based ☐ Both

Statistic type for inference (See Eklund et al. 2016): n/a

Correction: n/a

Models & analysis

n/a	Involved in the study
☒	☐ Functional and/or effective connectivity
☒	☐ Graph analysis
☒	☐ Multivariate modeling or predictive analysis
