# Supplementary material for: Cancer stem cells are hyper-responsive sensors of the tumour microenvironment and regulate metastasis dynamics through YAP/TAZ
Source: Nat Cell Biol. 2026 Jul 27;28(8):1728–41. doi: 10.1038/s41556-026-02013-8 (PMC13457095; doi:10.1038/s41556-026-02013-8)
Supplement: Supplementary file 3 [file 41556_2026_2013_MOESM2_ESM.pdf]

Peer Review File

Cancer stem cells are hyper-responsive sensors of tumor microenvironment and regulate metastasis dynamics through YAP/TAZ

Corresponding Author: Dr Lalage Wakefield

Version 0:

Decision Letter:

*Please delete the link to your author homepage if you wish to forward this email to co-authors.

Dear Dr Wakefield,

Please first accept our apologies for the long delay getting back to you with a decision due to difficulties in retrieving reviewers' comments.

Your manuscript, "Cancer stem cells are hyper-responsive sensors of tumor microenvironment in orchestrating metastasis dynamics", has now been seen by 2 referees, who are experts in cancer stem cells, breast cancer (referee 1); and cancer stem cells (referee 3). As you will see from their comments (attached below) they find this work of potential interest, but have raised substantial concerns, which in our view would need to be addressed with considerable revisions before we can consider publication in Nature Cell Biology.

We would like to clarify that although we have engaged a third referee with expertise on brest cancer and metastasis on the referee panel, the comments raised by the other two referees were sufficient for us to form a decision in the absence of this expert's feedback, and we felt a further delay would be counterproductive for the authors. We will send you the third report if/when we receive it.

Nature Cell Biology editors discuss the referee reports in detail within the editorial team, including the chief editor, to identify key referee points that should be addressed with priority, and requests that are overruled as being beyond the scope of the current study. To guide the scope of the revisions, I have listed these points below. We are committed to providing a fair and constructive peer-review process, so please feel free to contact me if you would like to discuss any of the referee comments further.

In particular, it would be essential to:

A) Provide proper organoid and in vivo models to further support the conclusions:

Reviewer 1
"All in vitro assays reported are done in 2D cultures. Since 3D tumor spheres enrich for CSC’s and may be more physiologically relevant than 2D cultures, it would be advantageous to examine CSC kinetics in 3D culture."

" The authors demonstrate that knockdown of YAP or TAZ reduced the CSC population in vitro (Fig 5F). To confirm the relevance of this to metastases, they should demonstrate the reduced metastatic capacity of these knockdown cells in the mouse models."

Reviewer 3
"...Targetting YAP TAZ may be useful to address one aspect of the TME, but this is a long way from showing translation to a therapy to reduce met lesions."

"My other main comment was a notable lack of use of organoid models (or similar eg , tumoruids) in this study. There is a complete reliance on animal models and cell lines as far as I can ascertain. Yet, if the goal is to inform development of therapies, one might expect organoid studies are included. What was the rationale for omitting this approach when 3D organoid/spheroid/ tumoroid models can provide good insight to the mechanism of drug effects on cell growth, metabolism and a host of other features?"

"In figure 3, there is a switch to in vitro work, and I found this transition to be hard to follow as someone not overly familiar with this type of direct comparison between in vivo and in vitro.
What or how did the authors justify the validity of comparing the microenvironmental influences on cell growth or differentiation (of CSC or nonCSC) in vivo to microenvironmental factors when cells are grown in a dish or transwell. Referring to my point above, did they attempt to use organoid models to study the influences of the TME?"

B) Strengthen the proposed working mechanism:

Reviewer 1
"The authors describe cell fate choices of CSC but they do not discuss the possibility of CSC generation via de-differentiation of non-CSC into CSC. Do they detect evidence for this occurring in vitro or in vivo particularly after chemotherapy challenge?"

"How does activation of the HIPPO pathway, YAP/TAZ/TEAD regulate the cell fate decisions between symmetrical, asymmetrical and differentiation divisions?"

Reviewer 3
"...There are a multitude of factors in the TME that could influence CSC dynamics and this report focuses mainly on the negative influence of nutrients and cell density/tumor size. What about other parameters such as matrix stiffness in the met?..."

C) All other referee concerns pertaining to strengthening existing data, providing controls, methodological details, clarifications and textual changes as applicable should also be addressed.

D) Finally please pay close attention to our guidelines on statistical and methodological reporting (listed below) as failure to do so may delay the reconsideration of the revised manuscript. In particular please provide:

- a Supplementary Figure including unprocessed images of all gels/blots in the form of a multi-page pdf file. Please ensure that blots/gels are labeled and the sections presented in the figures are clearly indicated.

- a Supplementary Table including all numerical source data in Excel format, with data for different figures provided as different sheets within a single Excel file. The file should include source data giving rise to graphical representations and statistical descriptions in the paper and for all instances where the figures present representative experiments of multiple independent repeats, the source data of all repeats should be provided.

We would be happy to consider a revised manuscript that would satisfactorily address these points, unless a similar paper is published elsewhere, or is accepted for publication in Nature Cell Biology in the meantime.

When revising the manuscript please:

- ensure that it conforms to our format instructions and publication policies (see below and www.nature.com/nature/authors/).

- provide a point-by-point rebuttal to the full referee reports verbatim, as provided at the end of this letter.

- provide the completed Editorial Policy Checklist (found here https://www.nature.com/authors/policies/Policy.pdf), and Reporting Summary (found here https://www.nature.com/authors/policies/ReportingSummary.pdf). This is essential for reconsideration of the manuscript and these documents will be available to editors and referees in the event of peer review. For more information see http://www.nature.com/authors/policies/availability.html or contact me.

Nature Cell Biology is committed to improving transparency in authorship. As part of our efforts in this direction, we are now requesting that all authors identified as ‘corresponding author’ on published papers create and link their Open Researcher and Contributor Identifier (ORCID) with their account on the Manuscript Tracking System (MTS), prior to acceptance. ORCID helps the scientific community achieve unambiguous attribution of all scholarly contributions. You can create and link your ORCID from the home page of the MTS by clicking on ‘Modify my Springer Nature account’. For more information please visit please visit www.springernature.com/orcid.

Please submit the revised manuscript files and the point-by-point rebuttal to the referee comments using this link:

Link Redacted

*This url links to your confidential home page and associated information about manuscripts you may have submitted or be reviewing for us. If you wish to forward this email to co-authors, please delete the link to your homepage.

We would like to receive a revised submission within six months. We would be happy to consider a revision even after this timeframe, however if the resubmission deadline is missed and the paper is eventually published, the submission date will be the date when the revised manuscript was received.

We hope that you will find our referees' comments, and editorial guidance helpful. Please do not hesitate to contact me if there is anything you would like to discuss.

Best wishes,

Zhe Wang

Zhe Wang, PhD
Senior Editor
Nature Cell Biology

Tel: +44 (0) 207 843 4924
email: zhe.wang@nature.com

Reviewers' Comments:

Reviewer #1 (Remarks to the Author):

In this manuscript the authors utilize a fluorescent stemness reporter to monitor cancer stem cell (bcsc's) kinetics in vitro and in experimental mouse models of breast cancer lung metastasis. They demonstrate that bcsc fate decisions are regulated by feedback loops that converge on the hippo pathway YAP/TAZ /TEAD signaling. Importantly, they demonstrate that targeting upstream inputs to this pathway blocked chemotherapy induced enrichment of CSC in lung metastases. In general, the experiments are comprehensive and well reported and provide important novel insights into CSC regulation with important therapeutic implications. Several issues should be addressed to further strengthen the work.
1) The authors describe cell fate choices of CSC but they do not discuss the possibility of CSC generation via de-differentiation of non-CSC into CSC. Do they detect evidence for this occurring in vitro or in vivo particularly after chemotherapy challenge?
2) How does activation of the HIPPO pathway, YAP/TAZ/TEAD regulate the cell fate decisions between symmetrical, asymmetrical and differentiation divisions?
3) All in vitro assays reported are done in 2D cultures. Since 3D tumor spheres enrich for CSC's and may be more physiologically relevant than 2D cultures, it would be advantageous to examine CSC kinetics in 3D culture.
4) The authors demonstrate that knockdown of YAP or TAZ reduced the CSC population in vitro (Fig 5F). To confirm the relevance of this to metastases, they should demonstrate the reduced metastatic capacity of these knockdown cells in the mouse models.
5) The mouse models described include both human tumor cells (LM2) in immunodeficient mice as well as 4T1 mouse mammary tumors in immunocompetent mice. Do the authors detect any effect of the immune system on CSC kinetics? Similarly, do the TEAD or ROCK inhibitors demonstrate similar efficacy in these immunocompetent models?
6) The YAP/TAZ pathway has been shown to induce cell plasticity and EMT cell state conversion. Indeed, fig E5A shows that EMT was one of the major YAP/TAZ regulated pathways both in vitro and in vivo. The authors should include a more detailed discussion of the role of EMT in CSC state transitions.
7) The authors should also expand the discussion on the known metabolic regulatory effects of YAP/TAZ signaling.

Reviewer #3 (Remarks to the Author):

The goal of this work was to gain a better understanding of the dynamics of CSC in metastatic lesions arising from primary breast cancers. The overarching assumption that this would aid our ability to understand mechanisms of CSC resistance to therapy.
The main findings are there is an enrichment of CSC in CTCs from mouse models of breast cancer but that upon colonisation of the lung metastatic site, there is a selective inhibition of CSC vs nCSC coupling the growth potential of the metastatic lesion to availability of tumor resources and increasing cell density.
The data are original but I wonder at its significance, especially to inform therapeutic approaches to prevent or reduce metastases. There are a multitude of factors in the TME that could influence CSC dynamics and this report focuses mainly on the negative influence of nutrients and cell density/tumor size. What about other parameters such as matrix stiffness in the met? A matrix targeting approach has yielded some important insight for solid tumours including breast. Targetting YAP TAZ may be useful to address one aspect of the TME, but this is a long way from showing translation to a therapy to reduce met lesions.
My other main comment was a notable lack of use of organoid models (or similar eg , tumoruids) in this study. There is a complete reliance on animal models and cell lines as far as I can ascertain. Yet, if the goal is to inform development of therapies, one might expect organoid studies are included. What was the rationale for omitting this approach when 3D organoid/spheroid/ tumoroid models can provide good insight to the mechanism of drug effects on cell growth, metabolism and a host of other features?

The data are reasonably well presented and initial data in Fig 1 and 2 lay the foundation for the hypothesis they propose.
However , I find the imprecise use of the word tumor is confusing. Can they refer to primary vs metastatic tumors so that there is no assumption. For example, Fig 1g simply uses the label tumor, that I take to be the primary tumor and gives % CSC and nonCSC. Not surprisingly, there is enrichment of CSC in CTC population, but there is no analysis of CSC and nonCSC in the metastatic tumors. So how do these data show enrichment of CSC on arrival in the lung metastases as stated in the legend, don't we need to show analysis in the lung met as well?

Fig 2b needs orange grey colour bar information. Assume the CSC are orange as per fig 1. Fig c,d,e show a reduction in CSC % as met increases in size. Rest of data in fig 2 are reasonable support for model presented in Fig 2k.
In figure 3, there is a switch to in vitro work, and I found this transition to be hard to follow as someone not overly familiar with this type of direct comparison between in vivo and in vitro.
What or how did the authors justify the validity of comparing the microenvironmental influences on cell growth or differentiation (of CSC or nonCSC) in vivo to microenvironmental factors when cells are grown in a dish or transwell. Referring to my point above, did they attempt to use organoid models to study the influences of the TME?
Overall I found the manuscript not to be a substantive amount of work. The fundamental discovery and reporting of how CSC respond to TME the cues or act as sensors is interesting but not a significant insight that would adjust or advance my current thinking of the reciprocal interactions between CSC and the TME of a metastatic lesion of breast cancer.

GUIDELINES FOR MANUSCRIPT SUBMISSION TO NATURE CELL BIOLOGY

READABILITY OF MANUSCRIPTS – Nature Cell Biology is read by cell biologists from diverse backgrounds, many of whom are not

native English speakers. Authors should aim to communicate their findings clearly, explaining technical jargon that might be unfamiliar to non-specialists, and avoiding non-standard abbreviations. Titles and abstracts should concisely communicate the main findings of the study, and the background, rationale, results and conclusions should be clearly explained in the manuscript in a manner accessible to a broad cell biology audience. Nature Cell Biology uses British spelling.

MANUSCRIPT FORMAT – please follow the guidelines listed in our Guide to Authors regarding manuscript formats at Nature Cell Biology.

TITLE – should be no more than 100 characters including spaces, without punctuation and avoiding technical terms, abbreviations, and active verbs..

AUTHOR NAMES – should be given in full.

AUTHOR AFFILIATIONS – should be denoted with numerical superscripts (not symbols) preceding the names. Full addresses should be included, with US states in full and providing zip/post codes. The corresponding author is denoted by: "Correspondence should be addressed to [initials]."

ABSTRACT AND MAIN TEXT – please follow the guidelines that are specific to the format of your manuscript, as listed in our Guide to Authors (http://www.nature.com/ncb/pdf/ncb_gta.pdf) Briefly, Nature Cell Biology Articles, Resources and Technical Reports have 3500 words, including a 150 word abstract, and the main text is subdivided in Introduction, Results, and Discussion sections. Nature Cell Biology Letters have up to 2500 words, including a 180 word introductory paragraph (abstract), and the text is not subdivided in sections.

ACKNOWLEDGEMENTS – should be kept brief. Professional titles and affiliations are unnecessary. Grant numbers can be listed.

AUTHOR CONTRIBUTIONS – must be included after the Acknowledgements, detailing the contributions of each author to the paper (e.g. experimental work, project planning, data analysis etc.). Each author should be listed by his/her initials.

FINANCIAL AND NON-FINANCIAL COMPETING INTERESTS – the authors must include one of three declarations: (1) that they have no financial and non-financial competing interests; (2) that they have financial and non-financial competing interests; or (3) that they decline to respond, after the Author Contributions section. This statement will be published with the article, and in cases where financial and non-financial competing interests are declared, these will be itemized in a web supplement to the article. For further details please see https://www.nature.com/licenceforms/nrg/competing-interests.pdf.

REFERENCES – are limited to a total of 70 for Articles, Resources, Technical Reports; and 40 for Letters. This includes references in the main text and Methods combined. References must be numbered sequentially as they appear in the main text, tables and figure legends and Methods and must follow the precise style of Nature Cell Biology references. References only cited in the Methods should be numbered consecutively following the last reference cited in the main text. References only associated with Supplementary Information (e.g. in supplementary legends) do not count toward the total reference limit and do not need to be cited in numerical continuity with references in the main text. Only published papers can be cited, and each publication cited should be included in the numbered reference list, which should include the manuscript titles. Footnotes are not permitted.

METHODS – Nature Cell Biology publishes methods online. The methods section should be provided as a separate Word document, which will be copyedited and appended to the manuscript PDF, and incorporated within the HTML format of the paper.

Methods should be written concisely, but should contain all elements necessary to allow interpretation and replication of the results. As a guideline, Methods sections typically do not exceed 3,000 words. The Methods should be divided into subsections listing reagents and techniques. When citing previous methods, accurate references should be provided and any alterations should be noted. Information must be provided about: antibody dilutions, company names, catalogue numbers and clone numbers for monoclonal antibodies; sequences of RNAi and cDNA probes/primers or company names and catalogue numbers if reagents are commercial; cell line names, sources and information on cell line identity and authentication. Animal studies and experiments involving human subjects must be reported in detail, identifying the committees approving the protocols. For studies involving human subjects/samples, a statement must be included confirming that informed consent was obtained. Statistical analyses and information on the reproducibility of experimental results should be provided in a section titled “Statistics and Reproducibility”.

All Nature Cell Biology manuscripts submitted on or after March 21 2016 must include a Data availability statement at the end of the Methods section. For Springer Nature policies on data availability see http://www.nature.com/authors/policies/availability.html; for more information on this particular policy see http://www.nature.com/authors/policies/data/data-availability-statements-data-citations.pdf. The Data availability statement should include:

• Accession codes for primary datasets (generated during the study under consideration and designated as "primary accessions") and secondary datasets (published datasets reanalysed during the study under consideration, designated as "referenced accessions"). For primary accessions data should be made public to coincide with publication of the manuscript. A list of data types for which submission to community-endorsed public repositories is mandated (including sequence, structure, microarray, deep sequencing data) can be found here http://www.nature.com/authors/policies/availability.html#data.

• Unique identifiers (accession codes, DOIs or other unique persistent identifier) and hyperlinks for datasets deposited in an approved repository, but for which data deposition is not mandated (see here for details http://www.nature.com/sdata/data-policies/repositories).

• At a minimum, please include a statement confirming that all relevant data are available from the authors, and/or are included with the manuscript (e.g. as source data or supplementary information), listing which data are included (e.g. by figure panels and data types) and mentioning any restrictions on availability.

• If a dataset has a Digital Object Identifier (DOI) as its unique identifier, we strongly encourage including this in the Reference list and citing the dataset in the Methods.

We recommend that you upload the step-by-step protocols used in this manuscript to protocols.io. More details can found at https://www.protocols.io/help/publish-articles.

DISPLAY ITEMS – main display items are limited to 6-8 main figures and/or main tables for Articles, Resources, Technical Reports; and 5 main figures and/or main tables for Letters. For Supplementary Information see below.

FIGURES – Colour figure publication costs $600 for the first, and $300 for each subsequent colour figure. All panels of a multi-panel figure must be logically connected and arranged as they would appear in the final version. Unnecessary figures and figure panels should be avoided (e.g. data presented in small tables could be stated briefly in the text instead).

All imaging data should be accompanied by scale bars, which should be defined in the legend.
Cropped images of gels/blots are acceptable, but need to be accompanied by size markers, and to retain visible background signal within the linear range (i.e. should not be saturated). The boundaries of panels with low background have to be demarked with black lines. Splicing of panels should only be considered if unavoidable, and must be clearly marked on the figure, and noted in the legend with a statement on whether the samples were obtained and processed simultaneously. Quantitative comparisons between samples on different gels/blots are discouraged; if this is unavoidable, it should only be performed for samples derived from the same experiment with gels/blots were processed in parallel, which needs to be stated in the legend.

Figures should be provided at approximately the size that they are to be printed at (single column is 86 mm, double column is 170 mm) and should not exceed an A4 page (8.5 x 11"). Reduction to the scale that will be used on the page is not necessary, but multi-panel figures should be sized so that the whole figure can be reduced by the same amount at the smallest size at which essential details in each panel are visible. In the interest of our colour-blind readers we ask that you avoid using red and green for contrast in figures. Replacing red with magenta and green with turquoise are two possible colour-safe alternatives. Lines with widths of less than 1 point should be avoided. Sans serif typefaces, such as Helvetica (preferred) or Arial should be used. All text that forms part of a figure should be rewritable and removable.

We accept files from the following graphics packages in either PC or Macintosh format:

- For line art, graphs, charts and schematics we prefer Adobe Illustrator (.AI), Encapsulated PostScript (.EPS) or Portable Document Format (.PDF). Files should be saved or exported as such directly from the application in which they were made, to allow us to restyle them according to our journal house style.

- We accept PowerPoint (.PPT) files if they are fully editable. However, please refrain from adding PowerPoint graphical effects to objects, as this results in them outputting poor quality raster art. Text used for PowerPoint figures should be Helvetica (preferred) or Arial.

- We do not recommend using Adobe Photoshop for designing figures, but we can accept Photoshop generated (.PSD or .TIFF) files only if each element included in the figure (text, labels, pictures, graphs, arrows and scale bars) are on separate layers. All text should be editable in 'type layers' and line-art such as graphs and other simple schematics should be preserved and embedded within 'vector smart objects' - not flattened raster/bitmap graphics.

- Some programs can generate Postscript by 'printing to file' (found in the Print dialogue). If using an application not listed above, save the file in PostScript format or email our Art Editor, Allen Beattie for advice (a.beattie@nature.com).

Regardless of format, all figures must be vector graphic compatible files, not supplied in a flattened raster/bitmap graphics format, but should be fully editable, allowing us to highlight/copy/paste all text and move individual parts of the figures (i.e. arrows, lines, x and y axes, graphs, tick marks, scale bars etc.). The only parts of the figure that should be in pixel raster/bitmap format are photographic images or 3D rendered graphics/complex technical illustrations.

All placed images (i.e. a photo incorporated into a figure) should be on a separate layer and independent from any superimposed scale bars or text. Individual photographic images must be a minimum of 300+ DPI (at actual size) or kept constant from the original picture acquisition and not decreased in resolution post image acquisition. All colour artwork should be RGB format.

FIGURE LEGENDS – must not exceed 350 words for each figure to allow fit on a single printed NCB page together with the figure. They must include a brief title for the whole figure, and short descriptions of each panel with definitions of the symbols used, but without detailing methodology.

TABLES – main tables should be provided as individual Word files, together with a brief title and legend. For supplementary tables see below.

SUPPLEMENTARY INFORMATION – Supplementary information is material directly relevant to the conclusion of a paper, but which cannot be included in the printed version in order to keep the manuscript concise and accessible to the general reader. Supplementary information is an integral part of a Nature Cell Biology publication and should be prepared and presented with as much care as the main display item, but it must not include non-essential data or text, which may be removed at the editor's discretion. All supplementary material is fully peer-reviewed and published online as part of the HTML version of the manuscript. Supplementary Figures and Supplementary Notes are appended at the end of the main PDF of the published manuscript.

Supplementary items should relate to a main text figure, wherever possible, and should be mentioned sequentially in the main manuscript, designated as Supplementary Figure, Table, Video, or Note, and numbered continuously (e.g. Supplementary Figure 1, Supplementary Figure 2, Supplementary Table 1, Supplementary Table 2 etc.).

Unprocessed scans of all key data generated through electrophoretic separation techniques need to be presented in a supplementary

figure that should be labelled and numbered as the final supplementary figure, and should be mentioned in every relevant figure legend. This figure does not count towards the total number of figures and is the only figure that can be displayed over multiple pages, but should be provided as a single file, in PDF or TIFF format. Data in this figure can be displayed in a relatively informal style, but size markers and the figures panels corresponding to the presented data must be indicated.

The total number of Supplementary Figures (not including the "unprocessed scans" Supplementary Figure) should not exceed the number of main display items (figures and/or tables (see our Guide to Authors and March 2012 editorial http://www.nature.com/ncb/authors/submit/index.html#suppinfo; http://www.nature.com/ncb/journal/v14/n3/index.html#ed). No restrictions apply to Supplementary Tables or Videos, but we advise authors to be selective in including supplemental data.

Each Supplementary Figure should be provided as a single page and as an individual file in one of our accepted figure formats and should be presented according to our figure guidelines (see above). Supplementary Tables should be provided as individual Excel files. Supplementary Videos should be provided as .avi or .mov files up to 50 MB in size. Supplementary Figures, Tables and Videos much be accompanied by a separate Word document including titles and legends.

GUIDELINES FOR EXPERIMENTAL AND STATISTICAL REPORTING

REPORTING REQUIREMENTS – To improve the quality of methods and statistics reporting in our papers we have recently revised the reporting checklist we introduced in 2013. We are now asking all life sciences authors to complete two items: an Editorial Policy Checklist (found here https://www.nature.com/authors/policies/Policy.pdf) that verifies compliance with all required editorial policies and a reporting summary (found here https://www.nature.com/authors/policies/ReportingSummary.pdf) that collects information on experimental design and reagents. These documents are available to referees to aid the evaluation of the manuscript. Please note that these forms are dynamic 'smart pdfs' and must therefore be downloaded and completed in Adobe Reader. We will then flatten them for ease of use by the reviewers. If you would like to reference the guidance text as you complete the template, please access these flattened versions at http://www.nature.com/authors/policies/availability.html.

STATISTICS – Wherever statistics have been derived the legend needs to provide the n number (i.e. the sample size used to derive statistics) as a precise value (not a range), and define what this value represents. Error bars need to be defined in the legends (e.g. SD, SEM) together with a measure of centre (e.g. mean, median). Box plots need to be defined in terms of minima, maxima, centre, and percentiles. Ranges are more appropriate than standard errors for small data sets. Wherever statistical significance has been derived, precise p values need to be provided and the statistical test used needs to be stated in the legend. Statistics such as error bars must not be derived from n<3. For sample sizes of n<5 please plot the individual data points rather than providing bar graphs. Deriving statistics from technical replicate samples, rather than biological replicates is strongly discouraged. Wherever statistical significance has been derived, precise p values need to be provided and the statistical test stated in the legend.

Information on how many times each experiment was repeated independently with similar results needs to be provided in the legends and/or Methods for all experiments, and in particular wherever representative experiments are shown.

We strongly recommend the presentation of source data for graphical and statistical analyses as a separate Supplementary Table, and request that source data for all independent repeats are provided when representative experiments of multiple independent repeats, or averages of two independent experiments are presented. This supplementary table should be in Excel format, with data for different figures provided as different sheets within a single Excel file. It should be labelled and numbered as one of the supplementary tables, titled "Statistics Source Data", and mentioned in all relevant figure legends.

--------- Please don't hesitate to contact NCB@nature.com should you have queries about any of the above requirements ---------

Version 1:

Decision Letter:

Our ref: NCB-A58496A

8th April 2026

Dear Dr. Wakefield,

Thank you for submitting your revised manuscript "Cancer stem cells are hyper-responsive sensors of tumor microenvironment in orchestrating metastasis dynamics" (NCB-A58496A). It has now been seen by the original referees and their comments are below. The reviewers find that the paper has improved in revision, and therefore we'll be happy in principle to publish it in Nature Cell Biology, pending minor revisions to satisfy the referees' final requests and to comply with our editorial and formatting guidelines.

If the current version of your manuscript is in a PDF format, please email us a copy of the file in an editable format (Microsoft Word or LaTex)-- we can not proceed with PDFs at this stage.

We are now performing detailed checks on your paper and will send you a checklist detailing our editorial and formatting requirements in about a week. Please do not upload the final materials and make any revisions until you receive this additional information from us.

Meanwhile, you may start to modify the manuscript files and figures according to editorial guidelines as below:

1. Please use the full space of main figures to present key data. Please kindly note that while re-organizing the figures, no peer-reviewed

data should be removed.
2. Please provide all figures separately from the main text, one figure per file;
3. Extended Data Table should be provided as a Supplementary Table item in Excel;

Thank you again for your interest in Nature Cell Biology Please do not hesitate to contact me if you have any questions.

Sincerely,

Zhe Wang, PhD
Senior Editor
Nature Cell Biology

Tel: +44 (0) 207 843 4924
email: zhe.wang@nature.com

Reviewer #1 (Remarks to the Author):

The authors have satisfactorily addressed my concerns.

Reviewer #3 (Remarks to the Author):

The authors have made revisions as requested, but the fact remains that the messaging from this work is that CSCs respond faster and/or more extensively to changing micro-environmental conditions than do nonCSCs and CSC are hyper-responsive to the microenvironment in the tumor. Based on this, they further propose that specifically targeting YAP/TAZ pathway is a concept that can be translated and has "therapeutic implications".

They have added new data in response to my questions and those of other referees.
However, there are two things that bother me about this manuscript.

The fact remains the work presented herein is all based on 2D not 3D models I completely understand that this is an issue for the field but organoids, microfluidic platforms, and 3D bioprinting technologies have been used to generate more biologically and physiologically relevant models. The field agrees that 2D models do not mimic the complex architecture and biological feature of tumors in real life and this is partly because the tumor microenvironment is oversimplified. Now that the FDA has removed the requirement for in vivo model evidence in drug development, it is really crucial that our in vitro models are as good as they can be, to ensure the predictive accuracy.

Another concern is the failure to model tumor heterogeneity, with the 2D models used herein and will continue to contribute to high attrition rates in clinical drug development. Nowadays a key factor in translating any discovery research is the recognition that breast cancer is a complex disease, partly related to the tumor heterogeneity. The selection of drugs is commonly tailored to tumor subtypes and individual patient tumors. A lack of patient-derived breast cancer models is another key limiting factor. Is the concept they developed applicable to any or all types of BC? This knowledge is generally needed to accurately predict individual drug responses or guide therapy selection.

Although they have genuinely attempted to work through some of the major technical issues, the facts remain that the translation of their concepts is slim. I consider the work to be a solid piece of discovery research but incremental, requiring refinement in superior models. For now, it has limited translational potential and therapeutic implications.

Version 2:

Decision Letter:

Dear Dr Wakefield,

I am pleased to inform you that your manuscript, "Cancer stem cells are hyper-responsive sensors of tumor microenvironment and regulate metastasis dynamics through YAP/TAZ", has now been accepted for publication in Nature Cell Biology.

Thank you for sending us the final manuscript files to be processed for print and online production, and for returning the manuscript checklists and other forms. Your manuscript will now be passed to our production team who will be in contact with you if there are any questions with the production quality of supplied figures and text.

Over the next few weeks, your paper will be copyedited to ensure that it conforms to Nature Cell Biology style. Once your paper is typeset, you will receive an email with a link to choose the appropriate publishing options for your paper and our Author Services team will be in touch regarding any additional information that may be required.

After the grant of rights is completed, you will receive a link to your electronic proof via email with a request to make any corrections within 48 hours. If, when you receive your proof, you cannot meet this deadline, please inform us at rjsproduction@springernature.com immediately.

You will not receive your proofs until the publishing agreement has been received through our system.

Due to the importance of these deadlines, we ask that you please let us know now whether you will be difficult to contact over the next month. If this is the case, we ask you provide us with the contact information (email, phone and fax) of someone who will be able to check the proofs on your behalf, and who will be available to address any last-minute problems.

If you have any questions about our publishing options, costs, Open Access requirements, or our legal forms, please contact ASJournals@springernature.com

Once your paper has been scheduled for online publication, the Nature press office will be in touch to confirm the details. An online order form for reprints of your paper is available at https://www.nature.com/reprints/author-reprints.html. All co-authors, authors' institutions and authors' funding agencies can order reprints using the form appropriate to their geographical region.

Publication is conditional on the manuscript not being published elsewhere and on there being no announcement of this work to any media outlet until the online publication date in Nature Cell Biology.

You may wish to make your media relations office aware of your accepted publication, in case they consider it appropriate to organize some internal or external publicity. Once your paper has been scheduled you will receive an email confirming the publication details. This is normally 3-4 working days in advance of publication. If you need additional notice of the date and time of publication, please let the production team know when you receive the proof of your article to ensure there is sufficient time to coordinate. Further information on our embargo policies can be found here: https://www.nature.com/authors/policies/embargo.html

Please note that *Nature Cell Biology* is a Transformative Journal (TJ). Authors may publish their research with us through the traditional subscription access route or make their paper immediately open access through payment of an article-processing charge (APC). Authors will not be required to make a final decision about access to their article until it has been accepted. Find out more about Transformative Journals

Authors may need to take specific actions to achieve compliance with funder and institutional open access mandates. If your research is supported by a funder that requires immediate open access (e.g. according to Plan S principles or the NIH public access policy) then you should select the gold OA route, and we will direct you to the compliant route where possible. Because authors warrant under our subscription licensing terms that they haven't committed to licensing any version of their article under a licence inconsistent with the terms of our agreement – including the applicable embargo period – publication under the subscription model isn't suitable for authors whose funders require no embargo.

To assist our authors in disseminating their research to the broader community, our SharedIt initiative provides you with a unique shareable link that will allow anyone (with or without a subscription) to read the published article. Recipients of the link with a subscription will also be able to download and print the PDF.

If your paper includes color figures, please be aware that in order to help cover some of the additional cost of four-color reproduction, Nature Portfolio charges our authors a fee for the printing of their color figures. Please contact our offices for exact pricing and details.

As soon as your article is published, you will receive an automated email with your shareable link.

If you have not already done so, we strongly recommend that you upload the step-by-step protocols used in this manuscript to protocols.io (https://protocols.io), an open online resource that allows researchers to share their detailed experimental know-how. All uploaded protocols are made freely available and are assigned DOIs for ease of citation. Protocols and Nature Portfolio journal papers in which they are used can be linked to one another, and this link is clearly and prominently visible in the online versions of both. Authors who performed the specific experiments can act as primary authors for the Protocol as they will be best placed to share the methodology details, but the Corresponding Author of the present research paper should be included as one of the authors. By uploading your Protocols onto protocols.io, you are enabling researchers to more readily reproduce or adapt the methodology you use, as well as increasing the visibility of your protocols and papers. You can also establish a dedicated workspace to collect your lab Protocols. Further information can be found at https://www.protocols.io/help/publish-articles.

You can use a single sign-on for all your accounts, view the status of all your manuscript submissions and reviews, access usage statistics for your published articles and download a record of your refereeing activity for the Nature Portfolio.

Nature Cell Biology encourages authors presenting evidence for cell, biological, molecular, and genetic interactions to consider communicating these findings using Biofactoid (https://biofactoid.org/). This tool helps users share a searchable representation of interactions (e.g. binding, gene expression, post-translational modification) between genes, gene products, or chemicals. Information added to Biofactoid, with author attribution, is shared on social media and public databases, such as Pathway Commons, where it can be discovered and analyzed in the context of a large and growing corpus of knowledge.

Please feel free to contact us if you have any questions.

With kind regards,

Zhe Wang, PhD
Senior Editor
Nature Cell Biology

Tel: +44 (0) 207 843 4924
email: zhe.wang@nature.com

Click here if you would like to recommend Nature Cell Biology to your librarian
http://www.nature.com/subscriptions/recommend.html#forms

** Visit the Springer Nature Editorial and Publishing website at www.springernature.com/editorial-and-publishing-jobs for more information about our career opportunities. If you have any questions please click here.**

Open Access This Peer Review File is licensed under a Creative Commons Attribution 4.0 International License, which permits use, sharing, adaptation, distribution and reproduction in any medium or format, as long as you give appropriate credit to the original author(s) and the source, provide a link to the Creative Commons license, and indicate if changes were made.
In cases where reviewers are anonymous, credit should be given to 'Anonymous Referee' and the source.
The images or other third party material in this Peer Review File are included in the article's Creative Commons license, unless indicated otherwise in a credit line to the material. If material is not included in the article's Creative Commons license and your intended use is not permitted by statutory regulation or exceeds the permitted use, you will need to obtain permission directly from the copyright holder.
To view a copy of this license, visit https://creativecommons.org/licenses/by/4.0/

Detailed Response to Reviewers' Comments:

Reviewer #1 (Remarks to the Author):

In this manuscript the authors utilize a fluorescent stemness reporter to monitor cancer stem cell (bcsc's) kinetics in vitro and in experimental mouse models of breast cancer lung metastasis. They demonstrate that bcsc fate decisions are regulated by feedback loops that converge on the hippo pathway YAP/TAZ /TEAD signaling. Importantly, they demonstrate that targeting upstream inputs to this pathway blocked chemotherapy induced enrichment of CSC in lung metastases. In general, the experiments are comprehensive and well reported and provide important novel insights into CSC regulation with important therapeutic implications. Several issues should be addressed to further strengthen the work.

1) The authors describe cell fate choices of CSC but they do not discuss the possibility of CSC generation via de-differentiation of non-CSC into CSC. Do they detect evidence for this occurring in vitro or in vivo particularly after chemotherapy challenge?

Response. This is a good point. We performed fate-mapping of LM2 nonCSCs *in vitro* to address the frequency of de-differentiation and have incorporated the results into the revised manuscript. (With intravital imaging it is difficult to capture enough of these rare events for statistical analysis.)

In untreated LM2 cultures, we find that the frequency of spontaneous dedifferentiation of nonCSCs to CSCs is 2 in 100 cells in 24h (**new Fig 3l**). The spontaneous differentiation frequency of the CSCs in the same cultures is 16 in 100 cells in 24h (**Fig. 3k**). Thus the forward conversion frequency of CSC to nonCSC is ~8x higher than the reverse conversion of nonCSC to CSC. (This aligns well with the 7.8x relative enrichment of tumor initiating activity in CSCs that we find in this breast cancer model (**Extended Fig. 1e**).) Despite this low dedifferentiation frequency, since the nonCSC population is much larger than the CSC population, even a low frequency of dedifferentiation could contribute significantly to the expansion of the CSC pool. We therefore performed origin mapping of CSCs in the same timeframe and showed that 22% of CSCs at t=60h came from de-differentiation of nonCSCs (**new Fig. 3m**). We have added a summary schematic of the results (**new Fig. 3n**) to aid in interpretation of the data, and new text (**lines 172-182**). Thus dedifferentiation of nonCSCs does contribute a small but significant fraction to the CSC pool in the highly aggressive LM2 breast cancer model.

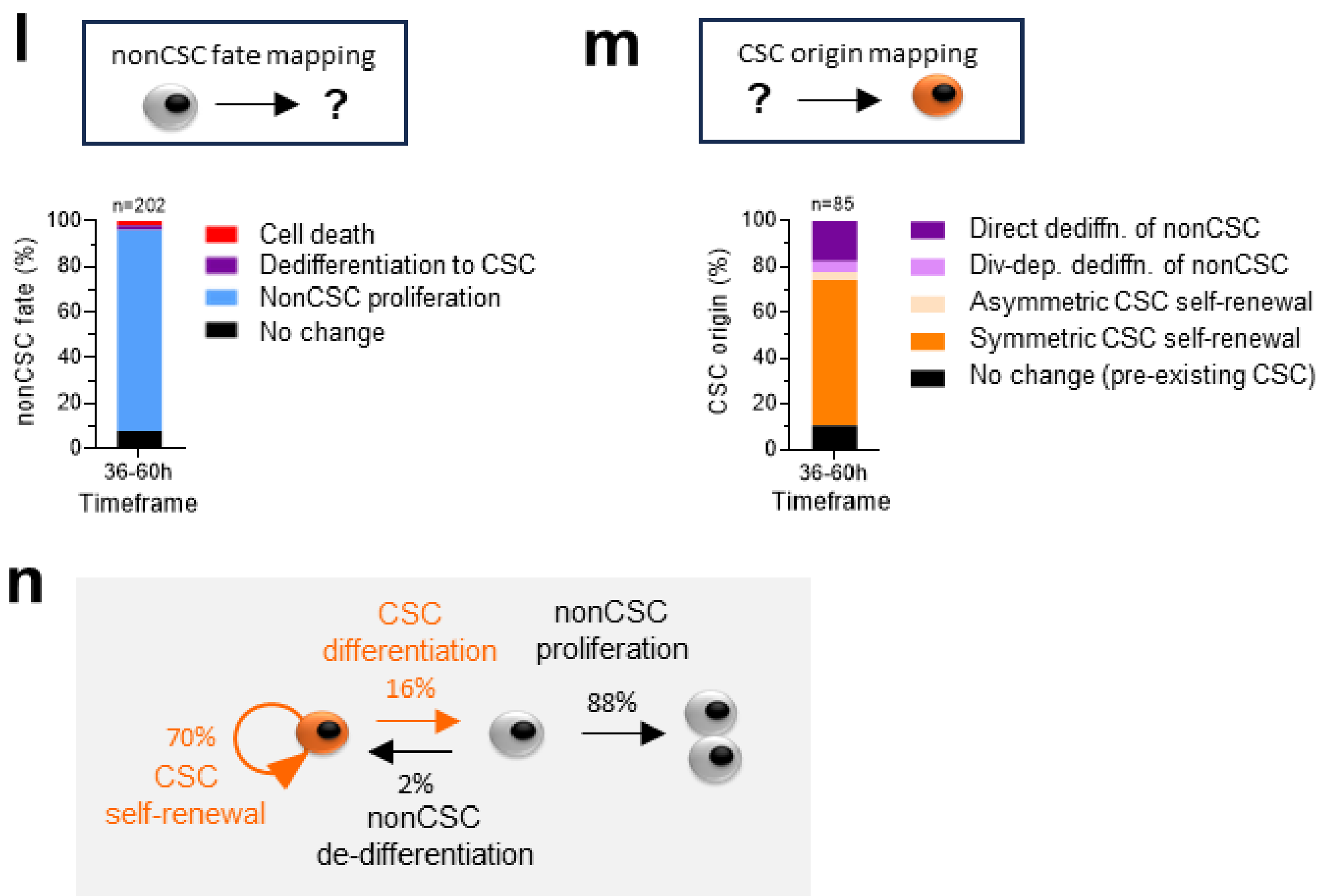

Fig. 3. ***l.*** *NonCSC cell fates in LM2 cultures in early (expansion) phase.* ***m.*** *Origin of CSCs present at t=60h (end of expansion phase) in LM2 cultures. Dediffn: dedifferentiation, Div-dep: division-dependent.* ***n.*** *Schematic for relative frequency of major cell fate changes in LM2 cultures in the 36-60h timeframe (expansion phase). Graphic shows % of mapped CSCs or nonCSCs undergoing the indicated fate change during the 24h period.*

In an independent experiment we addressed the same issue in paclitaxel-treated cultures as requested. We showed that the frequency of dedifferentiation of nonCSCs was still extremely low and not statistically different in paclitaxel-treated cultures (**new Extended Data Fig. 7a,b**).

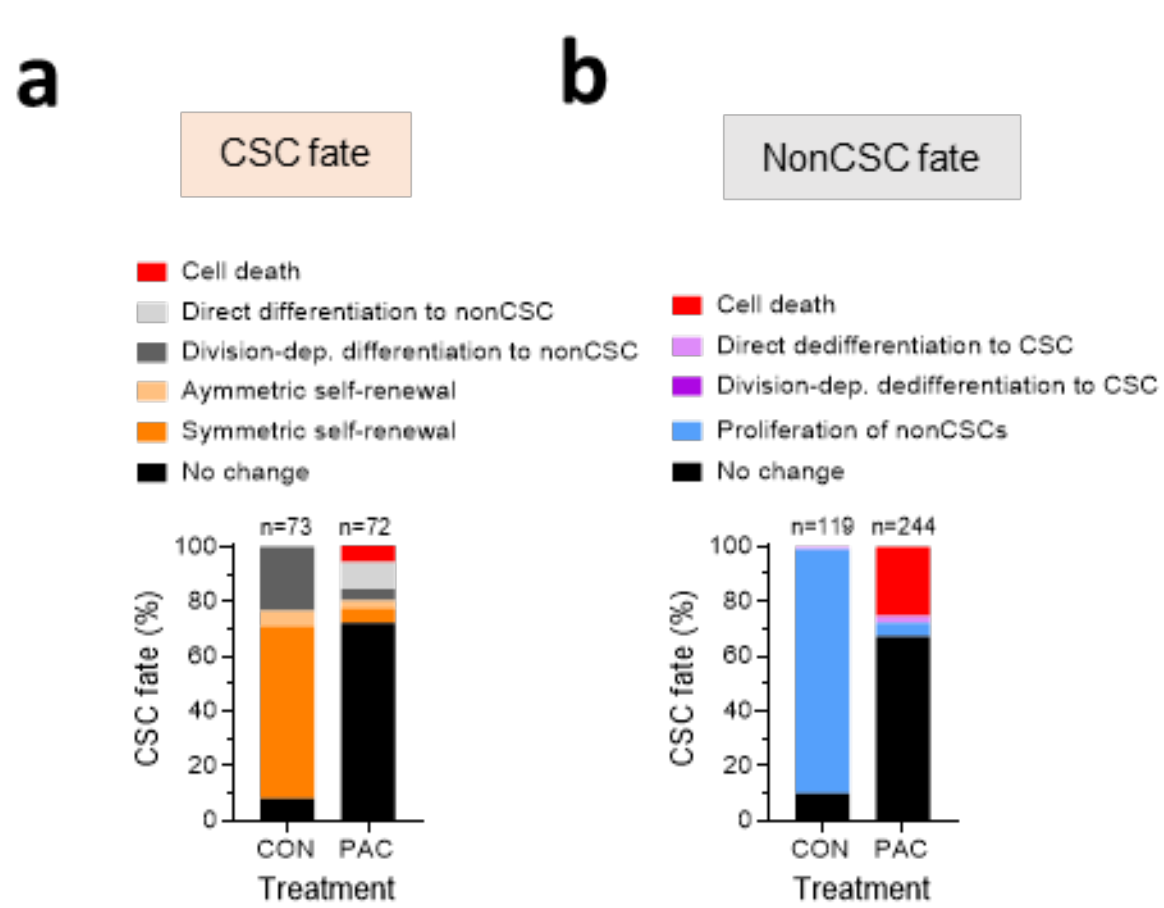

Extended Data Fig. 7. a,b *Fate mapping of CSC* ***(a)*** *and nonCSC* ***(b)*** *responses to paclitaxel in LM2 cells in vitro. Detailed statistics are given in statistical source data files.*

However, this new fate-mapping experiment also yielded the important information that paclitaxel treatment induced much more (~5x) cell death in nonCSCs than CSCs, as well as reducing nonCSC proliferation to a greater extent than that of CSCs (**new Fig. 7e,f**) and **new text lines 352-359**.

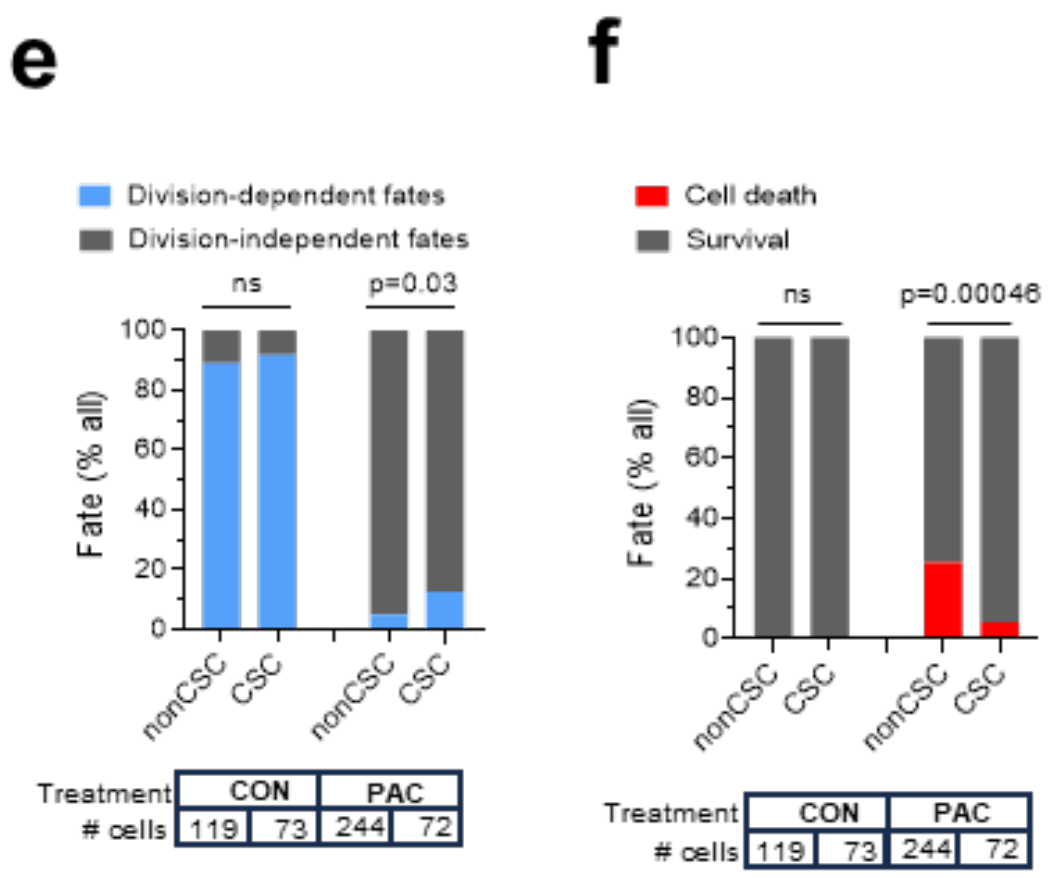

*Fig. 7. e,f. Effect of Paclitaxel on division-dependent fates **(e)** and cell death **(f)** of LM2 cells in culture from single cell fate mapping after 36h of Paclitaxel treatment. Fisher exact test.*

2) How does activation of the HIPPO pathway, YAP/TAZ/TEAD regulate the cell fate decisions between symmetrical, asymmetrical and differentiation divisions?

Response. YAP/TAZ knockdown decreases all cell division frequencies, consistent with the known pro-proliferative activity of this node (**Fig. 5h**). Among the remaining division-dependent events, YAP/TAZ knockdown selectively promotes a 3x increase in the proportion of symmetric differentiating divisions, and there are too few asymmetric mitoses for accurate quantitation (**new Extended Data Fig 4f**).

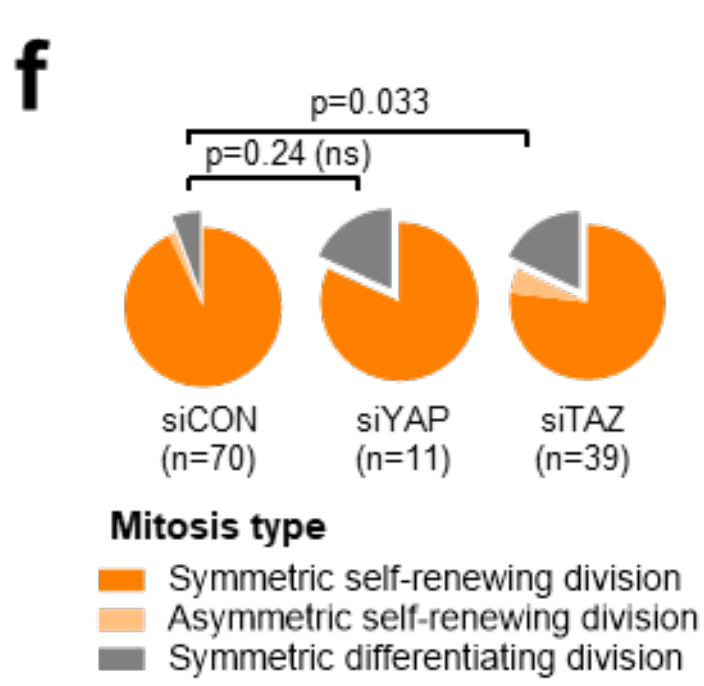

***Extended Data Fig. 4. f.** Proportion of different division-dependent events following YAP/TAZ knockdown in LM2 cells. n=total number of proliferation events tracked. Fisher Exact test for symmetric self-renewing mitoses.*

The biggest effect of YAP/TAZ knockdown on CSCs is a 3-6x increase in direct differentiation (division-independent) (**original Fig. 5h**). Thus YAP/TAZ normally supports expansive (symmetric) self-renewing mitoses in the CSC population and blocks both differentiating divisions and direct differentiation, without having a significant effect on the small number of asymmetric divisions (summarized in **schematic in new Fig. 5i** and discussed in lines **269-271**).

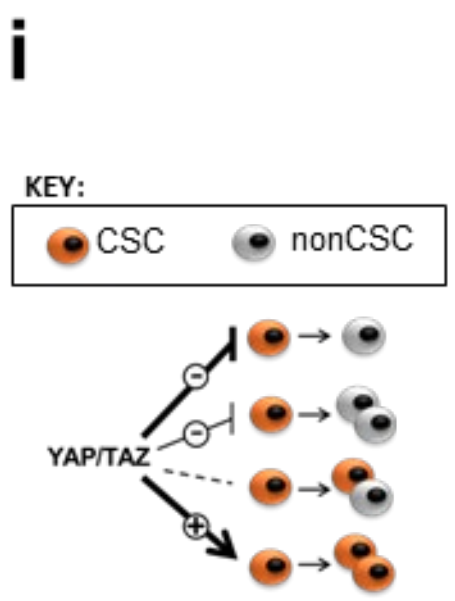

Fig. 5 i. *Schematic summarizing effect of YAP on CSC fates. Dotted line: event numbers too small to ascertain effect*

3) All in vitro assays reported are done in 2D cultures. Since 3D tumor spheres enrich for CSC's and may be more physiologically relevant than 2D cultures, it would be advantageous to examine CSC kinetics in 3D culture.

Response: This is an important point that was raised by both reviewers. We agree with both reviewers that 3D tumoroid/organoid cultures are generally more physiologically relevant than 2D cultures, though we are less dependent than others on the need to enrich for CSCs in our assays as we can directly observe them even when present in small numbers.

We previously spent nearly 2 years unsuccessfully trying to optimize longitudinal imaging over time of CSC and nonCSC populations in 3D culture. When we found unexpectedly that we could replicate in 2D culture on plastic many aspects of the CSC dynamics that we had seen in the metastases *in vivo*, we decided to use the 2D platform for our mechanistic analyses, since the results indicated that a significant fraction of the cues that were important *in vivo* might also be manifest in 2D culture.

However, in response to the editors' and reviewers' comments we tried to revisit the 3D culture approach, incorporating newer technological advances, such as new 3D culture matrices and AI-assisted image segmentation and analysis. Specifically, we tested (a)

classic anchorage-independent tumorsphere formation assays in liquid medium in low attachment wells, (b) semisolid tumorsphere assays using Methocult matrix; (c) 3D culture assays using PepGel PGmatrix, a synthetic peptide-based hydrogel. For the hydrogel experiments, we partnered with the company PepGel-Cellular Biomimicry NC (www.pepgel.com) which has extensive experience in use of these matrices and in 3D imaging.

Unfortunately, we continued to encounter the same significant limitations related to live imaging and quantitative analysis in 3D culture that we had encountered before. In designing fluorescent reporters, there is always a trade-off between rapid on/off kinetics with consequent high selectivity for the phenotype (stemness in this case) vs high sensitivity. We deliberately designed our reporter to maximize our ability to image dynamically, by destabilizing the fluorescent protein. An unavoidable consequence of this design feature is that the reporter is relatively weak. Accurate assessment of CSC self-renewal and differentiation kinetics in 3D requires longitudinal confocal imaging, in which individual spheroids are continuously tracked over multiple days with repeated Z-stack acquisition. Due to our relatively low reporter signal, longitudinal imaging necessitated higher laser power and extended exposure times, leading to rapid signal loss, photobleaching, phototoxicity and reduced spatial resolution, particularly when imaging deeper regions of the matrix. In addition, all the tested matrices exhibited substantial nonspecific background fluorescence, which compounded the sensitivity problem.

Collectively, these factors precluded reliable longitudinal tracking of individual spheroids and robust quantitative analysis of CSC dynamics in 3D culture. We regret that we were unable to achieve this goal. However, we feel that the good concordance of our 2D culture kinetics with our *in vivo* metastasis kinetics, and the fact that the mechanistic insights (regarding the role of the YAP/TAZ/TEAD node and upstream inputs) that we generated in 2D translated to pharmacologic interventions (with TEAD, LPAR and ROCK inhibitors) that had the predicted impact on CSC dynamics and metastasis outcome *in vivo* (**in original Fig. 7 and Extended Data Fig. 7**) leads us to believe that the 2D approach has been productive in generating new conceptual and therapeutic insights, even if the 3D approach would have been better.

4) The authors demonstrate that knockdown of YAP or TAZ reduced the CSC population in vitro (Fig 5F). To confirm the relevance of this to metastases, they should demonstrate the reduced metastatic capacity of these knockdown cells in the mouse models.

Response. We thank the reviewer for this suggestion. We used shRNA to stably knockdown YAP in LM2 cells, showing a relatively selective reduction in CSCs at the

knockdown level that we achieved (**new Extended data Fig. 4g**) and we demonstrated, as expected, that this intervention decreased their metastatic ability (**new Fig. 5j and text lines 271-274).** This result is in agreement with our previous data showing that pharmacologic inhibition of the YAP/TAZ/TEAD node with GNE7883 reduces metastatic burden in combination with Paclitaxel (**Extended Data Fig. 7d**).

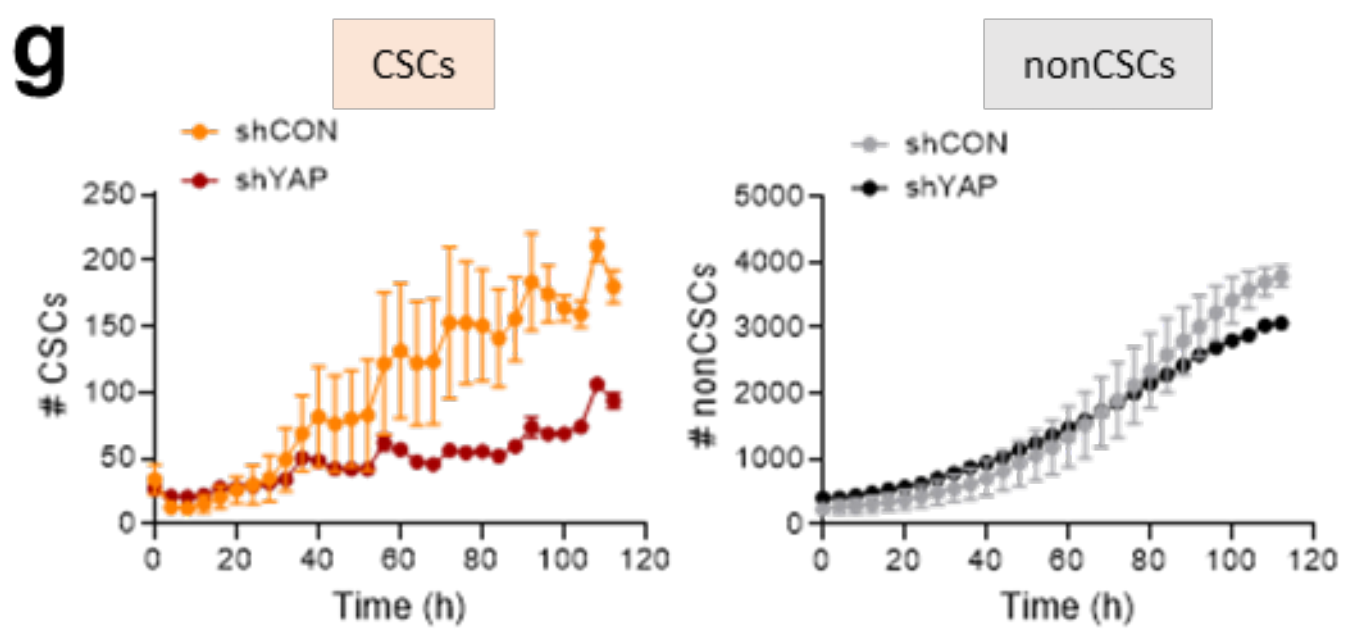

Extended Data Fig. 4g. *CSC and nonCSC expansion kinetics following shYAP knockdown in LM2 cells. Results are mean +/- SD for n=6*

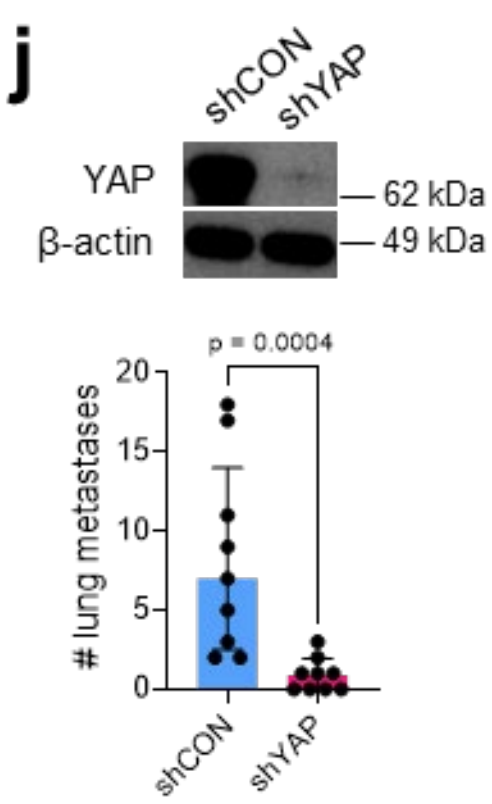

Fig. 5j. *Effect of stable YAP knockdown on metastatic efficiency of LM2 cells. Median +/- interquartile range for n=9 mice/group; Mann-Whitney U-test.*

5) The mouse models described include both human tumor cells (LM2) in immunodeficient mice as well as 4T1 mouse mammary tumors in immunocompetent mice. Do the authors detect any effect of the immune system on CSC kinetics?

Response. The question of how the immune system affects CSC kinetics is a really important and interesting one. In a previous publication, we showed that non-CSCs transiently contacting tumor-associated macrophages could re-acquire a CSC phenotype (https://pubmed.ncbi.nlm.nih.gov/34911937/), and others have indirectly shown the ability of non-lytic CD8 T cells to do so (https://pubmed.ncbi.nlm.nih.gov/30692216/).

Undoubtedly there will be important effects of adaptive immune cells on CSC kinetics but this is difficult for us to address with our current tools. The key stumbling block is that the mCherry fluorescent protein in the CSC reporter is immunogenic (as are all the commonly used fluorescent proteins). Cells expressing fluorescent proteins are generally eliminated in fully immunocompetent mice, and in our experience, single metastatic cells and small metastatic lesions are especially vulnerable to this immune elimination. Consequently we cannot directly observe CSC kinetics in the 4T1 model in immunocompetent mice, and the imaging experiments that we reported with the mouse 4T1 line (**Fig. 1d-g and Extended Fig. 2e-g**) were all done in immunodeficient animals, as was indicated in the Methods section. We have added text in Results **(lines 88-89)** to make clear that the 4T1 model was run in immunodeficient mice.

In the past, we tried to address this question by making a dseGFP version of our reporter and using it with the 4T1 model in the "Glowing Head" mouse https://pubmed.ncbi.nlm.nih.gov/25369133/. The Glowing Head mouse was designed to be tolerized to eGFP by transgenic expression of GFP in the pituitary. However, we still got rejection of the GFP-positive tumor cells, and we have since learned that this has been the experience of others too - likely due to the mouse only having peripheral not central tolerization to the neoantigen. Thus for now, we cannot address this important question directly with our reporter.

Similarly, do the TEAD or ROCK inhibitors demonstrate similar efficacy in these immunocompetent models?

Response. Both ROCK and YAP have context-dependent effects in multiple cell types in the immune system. Thus the integrated effect of ROCK/YAP inhibition on CSCs and immune cells *in vivo* may be an important determinant of therapeutic outcome. To address this issue, we tested efficacy of the ROCK inhibitor Y27632 in combination with Paclitaxel on endpoint metastatic burden in the 4T1 model in immunocompetent BALB/c hosts. (The experiment was run without imaging markers to avoid the immune rejection problem, so CSCs could not be quantitated in this experiment.) While paclitaxel alone was relatively ineffective in the 4T1 model, we found that the combination of paclitaxel with Y27632 showed a small but significant therapeutic benefit in reducing metastatic burden at endpoint in this immunocompetent model (**new Extended Fig. 7m,n**). This result gives hope that the efficacy we saw with CSC-targeting drugs in immunodeficient mice will extend to the immunocompetent setting. Further detailed exploration of this issue will be important but is beyond the scope of this manuscript.

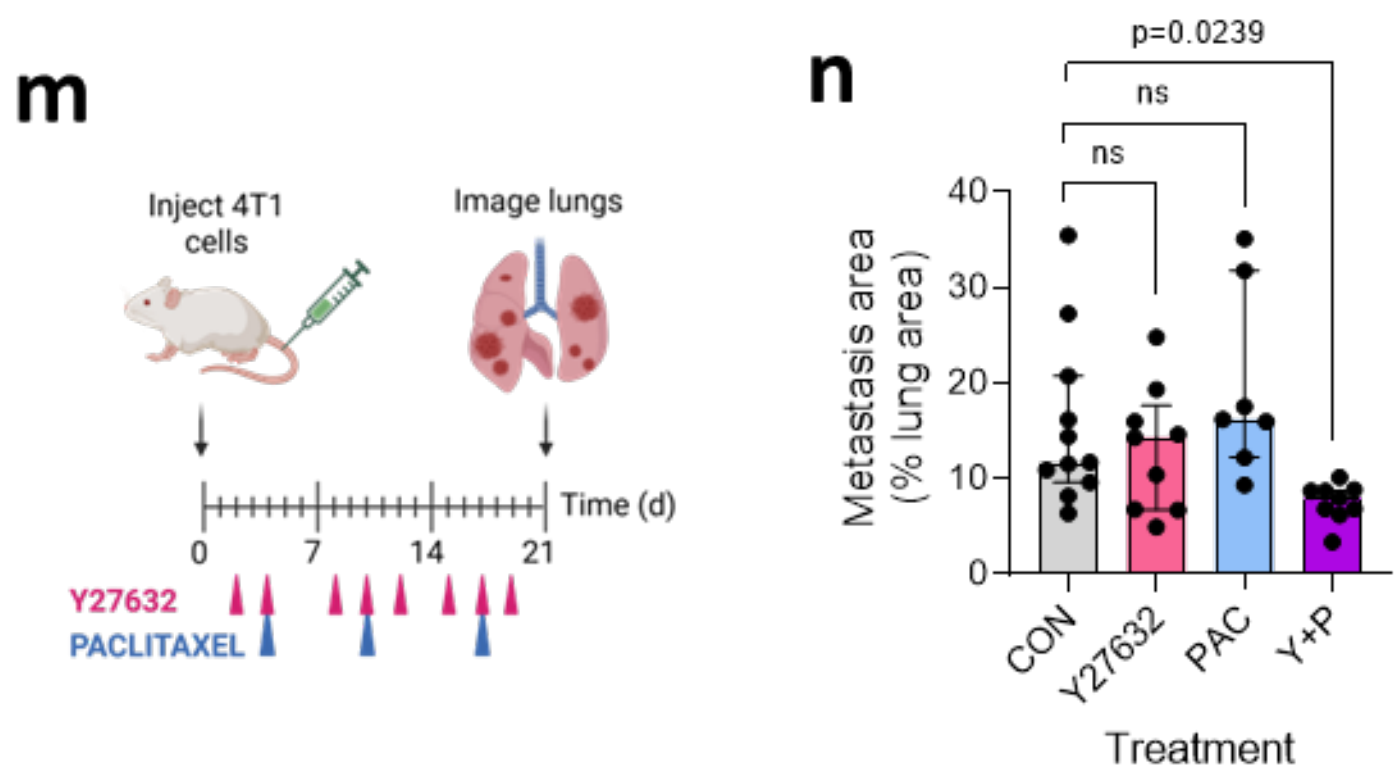

Extended Data Fig. 7m. *Schematic for treatment of 4T1 models with ROCK inhibitor Y27632 and Paclitaxel.* ***n.*** *Effect of therapeutic intervention on lung metastasis in 4T1 model in immunocompetent BALB/c mice. n=7-11 mice/group. Median +/- interquartile range. Dunn's multiple comparisons test for treatment groups vs control.*

6) The YAP/TAZ pathway has been shown to induce cell plasticity and EMT cell state conversion. Indeed, fig E5A shows that EMT was one of the major YAP/TAZ regulated pathways both in vitro and in vivo. The authors should include a more detailed discussion of the role of EMT in CSC state transitions.

In the original geneset enrichment analysis of our transcriptomic datasets, we found highly significant enrichment of a widely used EMT geneset in the CSCs (**original Extended Data Fig. 5a**: SARRIO_EPITHELIAL_MESENCHYMAL_TRANSITION_UP). However, when we examined the leading edge genes that were driving this enrichment, we found that they were all proliferation-related, and furthermore that the Sarrio signature was derived from an experimental setup to generate the "EMT signature" that had proliferation as a confounding variable (https://pubmed.ncbi.nlm.nih.gov/18281472/). Therefore we felt that the apparent enrichment for EMT in our own dataset was misleading, and we have removed these results from the original graph.

Nevertheless, a complex inter-relationship between EMT, YAP/TAZ and stemness is well-documented in the literature, so this is definitely an important question to address. Acquisition of the stemness phenotype and the EMT process overlap in sharing a requirement for phenotypic plasticity, suggesting there may be common mechanistic features. We approached this experimentally in the LM2 model by first looking at expression of the core EMT transcription factors. We saw no enrichment of core EMT-TFs in

LM2 CSCs, although core stem cell transcription factors were clearly enriched (**new Extended Data Fig. 6a**). However, we did find that one of our key TEAD target genes, ID1, had a binding site for the EMT-TF Snail close to the TEAD binding site (**new Extended Data Fig. 6b**). We therefore knocked down Snail and showed that this resulted in a reduction in ID1 expression, and consequently in CSCs (**new Extended Data Fig. 6c-d**). These results suggests that EMT-TFs may cooperate with TEADs to regulate expression of key stemness genes. We have included discussion of the EMT/YAP/stem inter-relationship **(lines 330-340).**

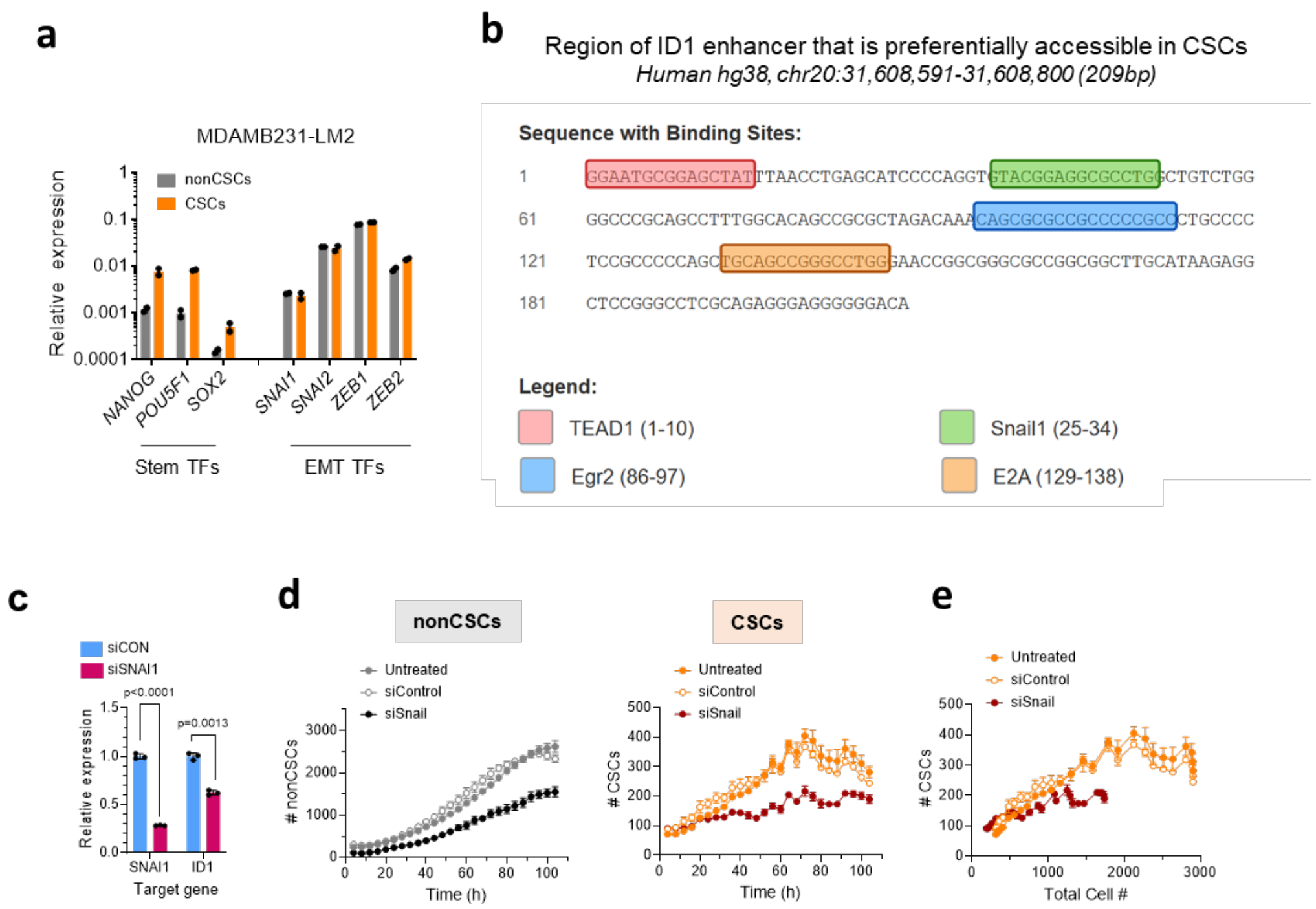

Extended Data Fig. 6. **a.** RTQ-PCR assessment of transcription factor mRNA expression in FACS-sorted LM2 CSCs and nonCSCs. Results are mean of n=2 **b.** Transcription factor binding sites in the preferentially accessible region in the ID1 gene enhancer in CSCs **c**. Effect of Snai1 knockdown on ID1 mRNA in LM2 cells. Mean +/- SD for n=3. **d**. Effect of Snail1 knockdown on LM2 nonCSC and CSC kinetics (from same culture but plotted separately for clarity). Mean +/- SD, n=3. **e**. Effect of Snail1 knockdown on CSC expansion in LM2 cells as a function of culture density. Mean +/- SD, n=3

7) The authors should also expand the discussion on the known metabolic regulatory effects of YAP/TAZ signaling.

We have added a brief discussion of this aspect in the Results **(lines 282-284)** and Discussion (**lines 434-437**), where we have also noted that this is a feature that needs to be

explored further in future studies.

Reviewer #3 (Remarks to the Author):

The goal of this work was to gain a better understanding of the dynamics of CSC in metastatic lesions arising from primary breast cancers. The overarching assumption that this would aid our ability to understand mechanisms of CSC resistance to therapy.
The main findings are there is an enrichment of CSC in CTCs from mouse models of breast cancer but that upon colonisation of the lung metastatic site, there is a selective inhibition of CSC vs nCSC coupling the growth potential of the metastatic lesion to availability of tumor resources and increasing cell density.
The data are original but I wonder at its significance, especially to inform therapeutic approaches to prevent or reduce metastases. There are a multitude of factors in the TME that could influence CSC dynamics and this report focuses mainly on the negative influence of nutrients and cell density/tumor size. What about other parameters such as matrix stiffness in the met? A matrix targeting approach has yielded some important insight for solid tumours including breast.

We absolutely agree with the reviewer that there will be a multitude of factors in the TME that could influence CSC dynamics. In our current study, we addressed some of those factors that we were readily able to model in 2D (trophic factor depletion, energy stress, cell crowding). We believe that with this approach, we were able to uncover an important general principle – namely that CSCs respond faster and/or more extensively to changing microenvironmental conditions than do nonCSCs. These observations led to our overarching concept of the CSC as a hyper-responsive sensor of the microenvironment in the tumor. We believe that this concept will apply to other TME inputs and can be studied in more detail by us and by others in followup work.

In this context, the issue of matrix composition and stiffness that the reviewer raises is a particularly important one that should be pursued in depth in the future. To stimulate such future work, we have added a figure showing that culture on type I collagen or fibronectin selectively increases the CSC population compared with culture on plastic (**new Fig. 4f**), and that matrix stiffness can also increase the CSC population (**new Fig. 4g**) .

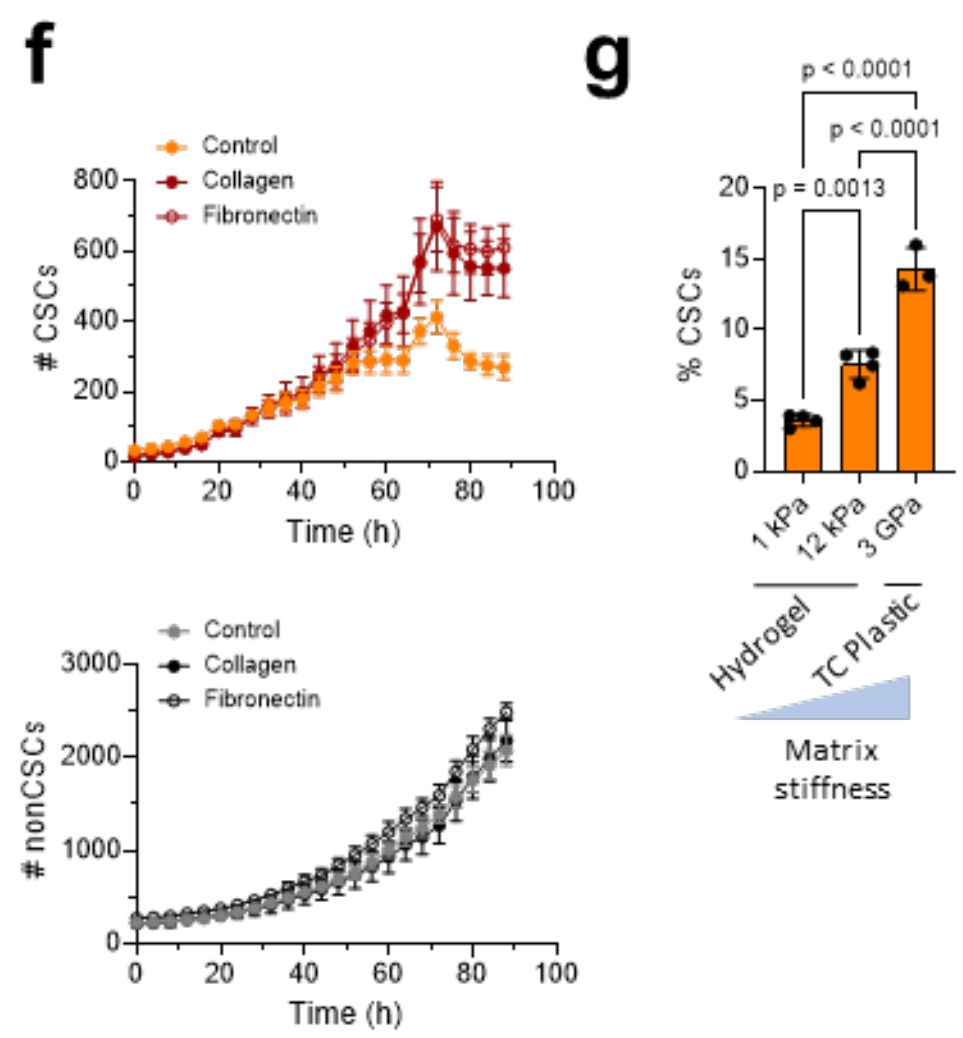

Fig. 4. **f.** Effect collagen or fibronectin coating of well on LM2 CSC and nonCSC dynamics. Mean +/- SD. **g.** Effect of matrix stiffness on relative CSC numbers by flow cytometry. Mean +/- SD, n=3. Tukey's multiple comparisons test.

Since collagen engagement activates focal adhesion kinases, our demonstration that the FAK inhibitor Defactinib selectively reduces CSCs (**Fig. 4e**) is in agreement with the reviewer's point that targeting other actionable inputs from the TME might have therapeutic potential. However, we acknowledge that much remains to be learned about CSC responses to these and other influences of the TME, and we have added discussion to this effect (Discussion **lines 437-438**). We hope that our work and the imaging tool that we have created will inspire others to develop these ideas in more detail.

Targetting YAP TAZ may be useful to address one aspect of the TME, but this is a long way from showing translation to a therapy to reduce met lesions.

Our data on TME inputs showed that most, if not all of the inputs that we studied in depth converged on the YAP/TAZ node. For proof-of-concept, we showed that targeting this node with the TEAD inhibitor GNE7832 could significantly reduce metastatic burden in the LM2 breast cancer model when combined with paclitaxel (**Extended Data Fig. 7c,d**). Furthermore, targeting TME inputs upstream of YAP/TAZ/TEAD with the ROCK inhibitor Y27632 or the LPAR inhibitor BMS986020 in combination with paclitaxel not only reduced metastatic burden, but also reversed the chemotherapy-induced enrichment of CSCs, completely ablating CSCs from a significant fraction of metastatic lesions (**see Fig 7i-l and Extended Fig 7e-l**). We also demonstrated modest but significant efficacy of Paclitaxel in

combination with Y27632 in reducing metastatic burden in the immunocompetent 4T1 model (**new Extended data Fig. 7m,n**). Thus we have now shown therapeutic efficacy ***in vivo*** for four independent model/therapy combinations based on YAP/TAZ pathway targeting plus chemotherapy. The intrinsic resistance of CSCs to most conventional therapies is a major cause of therapeutic failure and disease recurrence. We therefore believe that our results represent an encouraging first step towards identifying combinatorial therapeutic strategies to reduce metastatic burden through more effective targeting of the CSC subpopulation.

My other main comment was a notable lack of use of organoid models (or similar eg , tumoruids) in this study. There is a complete reliance on animal models and cell lines as far as I can ascertain. Yet, if the goal is to inform development of therapies, one might expect organoid studies are included. What was the rationale for omitting this approach when 3D organoid/spheroid/ tumoroid models can provide good insight to the mechanism of drug effects on cell growth, metabolism and a host of other features?

Response. We absolutely agree with the reviewer that 3D cultures will likely give insights into additional mechanisms regulating CSC dynamics that cannot be captured in 2D. We devoted considerable time and effort in the past to trying unsuccessfully to establish reliable methods for longitudinal imaging of CSC kinetics in organoids/tumoroids. We revisited this issue again in response to the reviewers' comments (please see response to reviewer 1 for details) but still were unable to overcome the many technical challenges. We have acknowledged this limitation in the Discussion (**lines 430-433**). However, since the key feature of CSC kinetics that we had seen in metastases in vivo (selective early shutdown of CSC expansion) was replicated in 2D culture, we reasoned that at least some of the important influences on CSC kinetics were being effectively modeled in 2D culture.

The data are reasonably well presented and initial data in Fig 1 and 2 lay the foundation for the hypothesis they propose.
However , I find the imprecise use of the word tumor is confusing. Can they refer to primary vs metastatic tumors so that there is no assumption. For example, Fig 1g simply uses the label tumor, that I take to be the primary tumor and gives % CSC and nonCSC.

Response. We apologize for the confusion and we have changed wording throughout to specify "primary tumor" or "metastasis" to avoid the ambiguity of the word "tumor".

Not surprisingly, there is enrichment of CSC in CTC population, but there is no analysis of CSC and nonCSC in the metastatic tumors. So how do these data show enrichment of CSC

on arrival in the lung metastases as stated in the legend, don't we need to show analysis in the lung met as well?

In relation to the important question of whether CSCs are enriched on arrival in the lung, but prior to the initiation of colonization, we analysed the identity of single cells in the lungs of mice with LM2 or 4T1 primary tumors orthotopically implanted in the mammary gland. Although the number of single disseminated cells in the lung is low in these models, we showed that the proportion of CSCs among the single metastatic tumor cells ("disseminated tumor cells") in the lung is essentially identical to the proportion of CSCs in the circulation. This **new data** has been added to the original **Fig. 1g** and noted in the text (**lines 97-99**).

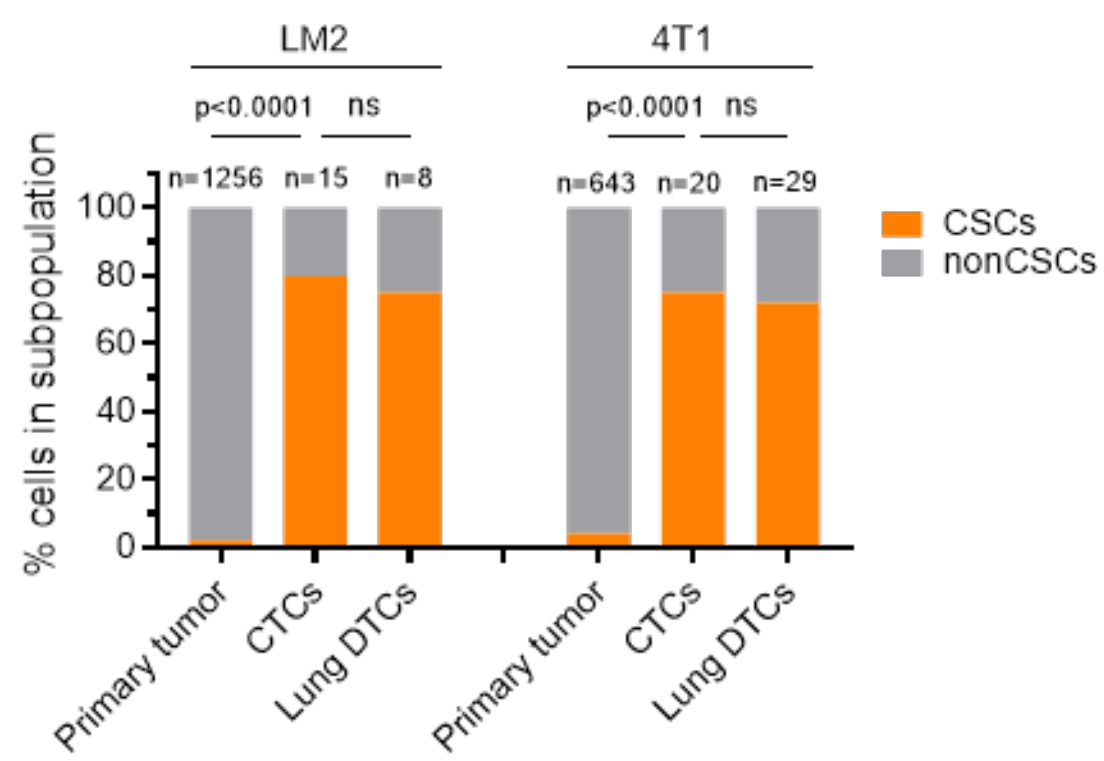

Fig. 1g. *Quantitation of % CSCs in primary tumor, circulating tumor cells (CTCs), and single disseminated tumor cells (DTCs) in lungs, from LM2 or 4T1 orthotopic primary tumors. Fisher Exact test. Number of cells (n) assessed is indicated for each condition, pooled from n=3 mice (LM2 primary tumor), n=4 mice (4T1 primary tumor), n= 4 mice (LM2 CTCs and DTCs) or n=9 mice (4T1 CTCs and DTCs). Source data are provided in the statistical source data file.*

Downstream of this initial seeding step, the data in the original Figure 2 provide a quite detailed analysis of CSCs and nonCSCs during the metastatic colonization process.

Fig 2b needs orange grey colour bar information. Assume the CSC are orange as per fig 1. Fig c,d,e show a reduction in CSC % as met increases in size. Rest of data in fig 2 are reasonable support for model presented in Fig 2k.

Response. We apologize for the omission of the color key in Fig 2b. It has been added back in.

In figure 3, there is a switch to in vitro work, and I found this transition to be hard to follow as someone not overly familiar with this type of direct comparison between in vivo and in

vitro. What or how did the authors justify the validity of comparing the microenvironmental influences on cell growth or differentiation (of CSC or nonCSC) in vivo to microenvironmental factors when cells are grown in a dish or transwell.

Response. Detailed molecular mechanistic analyses are challenging to perform in the *in vivo* setting. Consequently, we asked whether we might be able to model aspects of the *in vivo* CSC kinetics in an *in vitro* setting. We began by trying to use 3D models, but encountered major technical hurdles to using these for detailed kinetic analyses and intervention studies with reliable CSC quantitation (see response to reviewer 1 for details). To our surprise, we were able to model the most striking feature of the *in vivo* behavior– namely the very different population expansion kinetics for the CSC and nonCSC populations and the early CSC plateau - in 2D culture *in vitro*. This suggested that at least some of the key microenvironmental inputs might be present *in vitro*, so we proceeded to analyse these in depth, while recognizing that not all the relevant cues will be present. We have now explicitly noted this limitation in the Discussion (**lines 430-433**).

Referring to my point above, did they attempt to use organoid models to study the influences of the TME?

Response. Please see our response to reviewer #1 on this important issue, which was raised by both reviewers.

Overall I found the manuscript not to be a substantive amount of work. The fundamental discovery and reporting of how CSC respond to TME the cues or act as sensors is interesting but not a significant insight that would adjust or advance my current thinking of the reciprocal interactions between CSC and the TME of a metastatic lesion of breast cancer.

Response. Respectfully we would point out that our findings revealed a previously unrecognized hyper-responsiveness of CSCs to microenvironmental inputs that can be exploited as a therapeutic vulnerability, by inhibiting the YAP/TAZ node in various ways in combination with conventional chemotherapy. This concept is supported by our four proof-of-principle preclinical therapeutic intervention studies in the mouse models.

Additional items fixed from initial submission:

All Figs: exact p-values given

Fig. 3 SUM159 added in Fig. 3f (accidentally omitted in previous version)

Fig. 5 reorganized for better flow with additional data requested by reviewers

Fig. 5g: cell numbers for siYAP and siTAZ fate mapping were accidentally inverted and have been corrected

Fig. 5o: data used in generating this summary bubble plot are given in Extended Data Fig. 5a

Fig. E3a: plotted semi-log for better appreciation of plateau timing

Fig. E3b: images were flipped and have been corrected

Extended Data Table E1: updated with results of additional repeat experiments and made more granular in relation to types of metabolic stress, as there were some differences in the responses between stress types. No change in overall conclusions

Extended Data Table E2 removed and complete annotated gene lists given in Statistical Source Data instead (relating to Fig. 6f)

Response to Reviewers' Comments

Reviewer #1 (Remarks to the Author):

The authors have satisfactorily addressed my concerns.

Reviewer #3 (Remarks to the Author):

The authors have made revisions as requested, but the fact remains that the messaging from this work is that CSCs respond faster and/or more extensively to changing micro-environmental conditions than do nonCSCs and CSC are hyper-responsive to the microenvironment in the tumor. Based on this, they further propose that specifically targeting YAP/TAZ pathway is a concept that can be translated and has "therapeutic implications".

They have added new data in response to my questions and those of other referees. However, there are two things that bother me about this manuscript.

The fact remains the work presented herein is all based on 2D not 3D models I completely understand that this is an issue for the field but organoids, microfluidic platforms, and 3D bioprinting technologies have been used to generate more biologically and physiologically relevant models. The field agrees that 2D models do not mimic the complex architecture and biological feature of tumors in real life and this is partly because the tumor microenvironment is oversimplified. Now that the FDA has removed the requirement for in vivo model evidence in drug development, it is really crucial that our in vitro models are as good as they can be, to ensure the predictive accuracy.

Another concern is the failure to model tumor heterogeneity, with the 2D models used herein and will continue to contribute to high attrition rates in clinical drug development. Nowadays a key factor in translating any discovery research is the recognition that breast cancer is a complex disease, partly related to the tumor heterogeneity. The selection of drugs is commonly tailored to tumor subtypes and individual patient tumors. A lack of patient-derived breast cancer models is another key limiting factor. Is the concept they developed applicable to any or all types of BC? This knowledge is generally needed to accurately predict individual drug responses or guide therapy selection.

Although they have genuinely attempted to work through some of the major technical issues, the facts remain that the translation of their concepts is slim. I consider the work

to be a solid piece of discovery research but incremental, requiring refinement in superior models. For now, it has limited translational potential and therapeutic implications.

Response: The reviewer raises legitimate considerations about the limitations of translational models. We respectfully note that our mechanistic framework was not derived from 2D culture alone, but actually was grounded in and validated by *in vivo* observations in mouse models with microenvironmental cues intact (except for the absence of adaptive immunity in nude mice). Our subsequent mechanistic dissection was performed in 2D culture *in vitro* after we showed that we could reproduce a key aspect of the *in vivo* tumor cell dynamics – namely very different population expansion kinetics for the CSCs and nonCSCs (indicating a unique negative feedback on the CSCs) – in that setting.

It is true that in the context of drug development, 3D *in vitro* models will likely be more predictive than 2D models. This issue will need to be addressed if high throughput screens based on our CSC imaging technology are to be developed. Furthermore, there are undoubtedly additional microenvironmental inputs to the CSC sensor mechanism that we did not identify because of the limitations of the 2D *in vitro* studies. These can also be addressed in future studies. In the interim however, the biological concepts arising from our 2D mechanistic studies have already been useful in nominating molecular targets that we then proved to be efficacious in reducing CSCs when inhibited *in vivo*.

The issue of tumor heterogeneity is also an important one, particularly as it relates to precision medicine. Our study used several different models of TNBC with distinct oncogenic mutation profiles, and demonstrated broad applicability of the central concept of the CSC sensor, but with differences in the details of the dominant input pathways that likely reflect some aspect of inter-tumoral heterogeneity within this disease subtype. It will be important to determine whether the principles we have uncovered in triple negative breast cancer (TNBC) extend to other subtypes of breast cancer, or indeed to other epithelial tumor types.

Our therapeutic intervention studies were designed as proof-of-concept validations of the central biological finding, consistent with the scope of a conceptual discovery paper. Additional work in increasingly sophisticated 3D models will be important for developing the translational potential of the concept, and the incorporation of patient-derived breast cancer models such as PDXs should make future studies more relevant.

We have added the following text to the discussion, to note the reviewer's concerns:

Our discovery of distinct population kinetics for CSCs and nonCSCs was grounded in observations made in tumor-bearing mice *in vivo*. Surprisingly we were able to replicate key features in 2D culture, and establish the overarching principle that microenvironmental inputs impact CSCs more profoundly than nonCSCs. Mechanistic convergence on YAP/TAZ/TEAD was a consistent feature, but the relative contribution of different inputs varied among three TNBC models, likely reflecting the heterogeneous nature of the disease. Addressing this heterogeneity further, it will be important to determine if the CSC sensor concept extends to other breast cancer subtypes, and indeed to other epithelial tumors. The use of more sophisticated *in vitro* 3D models with greater microenvironmental complexity, and the incorporation of patient-derived material, will be important in improving the predictive value of *in vitro* approaches. Such models with their more realistic architecture and biology should also enable delineation of additional microenvironmental inputs beyond the ones we identified here.
